# Supplementary material for: Role and mechanism of NCAPD3 in promoting malignant behaviors in gastric cancer
Source: Front Pharmacol. 2024 Apr 22;15:1341039. doi: 10.3389/fphar.2024.1341039 (PMC11070777; doi:10.3389/fphar.2024.1341039)
Supplement: Supplementary file 11 [file DataSheet2.ZIP › GSEA/immunologic/my_analysis.Gsea.1599462003817/gsea_report_for_WT_1599462003817.html]

Report for WT 1599462003817 [GSEA]

| GS  follow link to MSigDB | GS DETAILS | SIZE | ES | NES | NOM p-val | FDR q-val | FWER p-val | RANK AT MAX | LEADING EDGE || 1 | GSE2770\_TGFB\_AND\_IL4\_VS\_IL4\_TREATED\_ACT\_CD4\_TCELL\_6H\_UP | Details ... | 25 | 0.47 | 2.17 | 0.000 | 0.840 | 0.746 | 658 | tags=88%, list=47%, signal=162% |
| 2 | GSE18791\_CTRL\_VS\_NEWCASTLE\_VIRUS\_DC\_4H\_UP | Details ... | 43 | 0.37 | 2.07 | 0.000 | 1.000 | 0.968 | 665 | tags=79%, list=47%, signal=145% |
| 3 | GSE18791\_CTRL\_VS\_NEWCASTLE\_VIRUS\_DC\_2H\_UP | Details ... | 32 | 0.41 | 2.05 | 0.002 | 0.806 | 0.981 | 661 | tags=84%, list=47%, signal=155% |
| 4 | GSE2706\_UNSTIM\_VS\_2H\_LPS\_DC\_UP | Details ... | 25 | 0.45 | 2.05 | 0.002 | 0.640 | 0.986 | 320 | tags=56%, list=23%, signal=71% |
| 5 | GSE8685\_IL2\_STARVED\_VS\_IL21\_ACT\_IL2\_STARVED\_CD4\_TCELL\_DN | Details ... | 16 | 0.52 | 2.03 | 0.005 | 0.598 | 0.992 | 207 | tags=50%, list=15%, signal=58% |
| 6 | GSE17974\_0H\_VS\_24H\_IN\_VITRO\_ACT\_CD4\_TCELL\_DN | Details ... | 25 | 0.44 | 2.01 | 0.002 | 0.565 | 0.997 | 773 | tags=96%, list=55%, signal=209% |
| 7 | GSE18791\_CTRL\_VS\_NEWCASTLE\_VIRUS\_DC\_8H\_UP | Details ... | 46 | 0.35 | 1.99 | 0.003 | 0.585 | 1.000 | 837 | tags=93%, list=59%, signal=222% |
| 8 | GSE2770\_UNTREATED\_VS\_ACT\_CD4\_TCELL\_48H\_UP | Details ... | 26 | 0.42 | 1.94 | 0.003 | 0.738 | 1.000 | 607 | tags=77%, list=43%, signal=133% |
| 9 | GSE18791\_CTRL\_VS\_NEWCASTLE\_VIRUS\_DC\_6H\_UP | Details ... | 34 | 0.38 | 1.93 | 0.005 | 0.718 | 1.000 | 702 | tags=82%, list=50%, signal=160% |
| 10 | GSE27786\_ERYTHROBLAST\_VS\_NEUTROPHIL\_DN | Details ... | 18 | 0.47 | 1.93 | 0.003 | 0.655 | 1.000 | 486 | tags=72%, list=34%, signal=109% |
| 11 | GSE14000\_UNSTIM\_VS\_4H\_LPS\_DC\_UP | Details ... | 24 | 0.42 | 1.92 | 0.010 | 0.601 | 1.000 | 821 | tags=100%, list=58%, signal=235% |
| 12 | GSE18791\_CTRL\_VS\_NEWCASTLE\_VIRUS\_DC\_10H\_UP | Details ... | 32 | 0.39 | 1.92 | 0.005 | 0.587 | 1.000 | 665 | tags=81%, list=47%, signal=150% |
| 13 | GSE6259\_DEC205\_POS\_DC\_VS\_CD4\_TCELL\_DN | Details ... | 17 | 0.48 | 1.91 | 0.002 | 0.571 | 1.000 | 637 | tags=88%, list=45%, signal=159% |
| 14 | GSE3982\_DC\_VS\_MAC\_DN | Details ... | 15 | 0.51 | 1.91 | 0.007 | 0.534 | 1.000 | 151 | tags=40%, list=11%, signal=44% |
| 15 | GSE5503\_LIVER\_DC\_VS\_SPLEEN\_DC\_ACTIVATED\_ALLOGENIC\_TCELL\_DN | Details ... | 20 | 0.45 | 1.90 | 0.002 | 0.512 | 1.000 | 523 | tags=75%, list=37%, signal=117% |
| 16 | GSE14415\_NATURAL\_TREG\_VS\_FOXP3\_KO\_NATURAL\_TREG\_DN | Details ... | 17 | 0.47 | 1.88 | 0.003 | 0.562 | 1.000 | 159 | tags=41%, list=11%, signal=46% |
| 17 | GSE21063\_3H\_VS\_16H\_ANTI\_IGM\_STIM\_BCELL\_DN | Details ... | 26 | 0.40 | 1.85 | 0.005 | 0.650 | 1.000 | 211 | tags=38%, list=15%, signal=44% |
| 18 | GSE37416\_CTRL\_VS\_3H\_F\_TULARENSIS\_LVS\_NEUTROPHIL\_UP | Details ... | 21 | 0.41 | 1.79 | 0.008 | 0.967 | 1.000 | 463 | tags=62%, list=33%, signal=91% |
| 19 | GSE45365\_WT\_VS\_IFNAR\_KO\_CD11B\_DC\_UP | Details ... | 22 | 0.40 | 1.77 | 0.008 | 0.983 | 1.000 | 292 | tags=50%, list=21%, signal=62% |
| 20 | GSE26030\_UNSTIM\_VS\_RESTIM\_TH17\_DAY15\_POST\_POLARIZATION\_UP | Details ... | 15 | 0.47 | 1.76 | 0.012 | 1.000 | 1.000 | 707 | tags=93%, list=50%, signal=185% |
| 21 | GSE28737\_BCL6\_HET\_VS\_BCL6\_KO\_MARGINAL\_ZONE\_BCELL\_DN |  | 22 | 0.41 | 1.75 | 0.013 | 1.000 | 1.000 | 542 | tags=68%, list=38%, signal=109% |
| 22 | GSE27786\_ERYTHROBLAST\_VS\_MONO\_MAC\_DN |  | 19 | 0.41 | 1.75 | 0.007 | 0.981 | 1.000 | 421 | tags=58%, list=30%, signal=81% |
| 23 | GSE20484\_MCSG\_VS\_CXCL4\_MONOCYTE\_DERIVED\_MACROPHAGE\_DN |  | 33 | 0.35 | 1.74 | 0.019 | 0.991 | 1.000 | 88 | tags=27%, list=6%, signal=28% |
| 24 | GSE5542\_UNTREATED\_VS\_IFNA\_TREATED\_EPITHELIAL\_CELLS\_24H\_DN |  | 16 | 0.44 | 1.73 | 0.009 | 1.000 | 1.000 | 429 | tags=63%, list=30%, signal=89% |
| 25 | GSE46606\_UNSTIM\_VS\_CD40L\_IL2\_IL5\_1DAY\_STIMULATED\_IRF4\_KO\_BCELL\_UP |  | 18 | 0.43 | 1.73 | 0.014 | 0.990 | 1.000 | 623 | tags=83%, list=44%, signal=147% |
| 26 | GSE37534\_GW1929\_VS\_ROSIGLITAZONE\_TREATED\_CD4\_TCELL\_PPARG1\_FOXP3\_TRANSDUCED\_DN |  | 15 | 0.45 | 1.73 | 0.026 | 0.978 | 1.000 | 152 | tags=40%, list=11%, signal=44% |
| 27 | GSE18791\_UNSTIM\_VS\_NEWCATSLE\_VIRUS\_DC\_10H\_DN |  | 21 | 0.39 | 1.72 | 0.015 | 0.978 | 1.000 | 405 | tags=57%, list=29%, signal=79% |
| 28 | GSE5542\_IFNG\_VS\_IFNA\_AND\_IFNG\_TREATED\_EPITHELIAL\_CELLS\_6H\_UP |  | 20 | 0.41 | 1.71 | 0.017 | 0.984 | 1.000 | 415 | tags=60%, list=29%, signal=84% |
| 29 | GSE8685\_IL2\_STARVED\_VS\_IL2\_ACT\_IL2\_STARVED\_CD4\_TCELL\_UP |  | 16 | 0.44 | 1.71 | 0.013 | 0.985 | 1.000 | 78 | tags=31%, list=6%, signal=33% |
| 30 | GSE40666\_STAT1\_KO\_VS\_STAT4\_KO\_CD8\_TCELL\_UP |  | 16 | 0.43 | 1.70 | 0.020 | 0.980 | 1.000 | 704 | tags=94%, list=50%, signal=185% |
| 31 | GSE11961\_MARGINAL\_ZONE\_BCELL\_VS\_MEMORY\_BCELL\_DAY40\_DN |  | 22 | 0.38 | 1.70 | 0.022 | 0.997 | 1.000 | 497 | tags=59%, list=35%, signal=90% |
| 32 | GSE15750\_WT\_VS\_TRAF6KO\_DAY6\_EFF\_CD8\_TCELL\_UP |  | 22 | 0.38 | 1.69 | 0.020 | 1.000 | 1.000 | 436 | tags=59%, list=31%, signal=84% |
| 33 | GSE17322\_CD103\_POS\_VS\_CD11B\_HIGH\_LUNG\_DC\_DN |  | 25 | 0.37 | 1.68 | 0.035 | 0.998 | 1.000 | 729 | tags=84%, list=52%, signal=171% |
| 34 | GSE3982\_EOSINOPHIL\_VS\_TH2\_DN |  | 31 | 0.34 | 1.68 | 0.017 | 0.992 | 1.000 | 868 | tags=97%, list=62%, signal=246% |
| 35 | GSE2706\_UNSTIM\_VS\_2H\_LPS\_AND\_R848\_DC\_UP |  | 25 | 0.37 | 1.68 | 0.013 | 0.975 | 1.000 | 639 | tags=80%, list=45%, signal=144% |
| 36 | GSE32533\_WT\_VS\_MIR17\_KO\_ACT\_CD4\_TCELL\_DN |  | 15 | 0.43 | 1.68 | 0.029 | 0.954 | 1.000 | 490 | tags=67%, list=35%, signal=101% |
| 37 | GSE15735\_CTRL\_VS\_HDAC\_INHIBITOR\_TREATED\_CD4\_TCELL\_12H\_UP |  | 21 | 0.38 | 1.67 | 0.017 | 0.981 | 1.000 | 222 | tags=38%, list=16%, signal=45% |
| 38 | GSE2770\_IL12\_ACT\_VS\_ACT\_CD4\_TCELL\_48H\_DN |  | 20 | 0.39 | 1.67 | 0.028 | 0.969 | 1.000 | 809 | tags=95%, list=57%, signal=220% |
| 39 | GSE14000\_UNSTIM\_VS\_16H\_LPS\_DC\_TRANSLATED\_RNA\_UP |  | 27 | 0.35 | 1.64 | 0.035 | 1.000 | 1.000 | 231 | tags=37%, list=16%, signal=43% |
| 40 | GSE45365\_HEALTHY\_VS\_MCMV\_INFECTION\_CD11B\_DC\_IFNAR\_KO\_UP |  | 23 | 0.37 | 1.62 | 0.042 | 1.000 | 1.000 | 268 | tags=43%, list=19%, signal=53% |
| 41 | GSE17974\_0H\_VS\_48H\_IN\_VITRO\_ACT\_CD4\_TCELL\_DN |  | 27 | 0.34 | 1.62 | 0.044 | 1.000 | 1.000 | 500 | tags=59%, list=35%, signal=90% |
| 42 | GSE46606\_UNSTIM\_VS\_CD40L\_IL2\_IL5\_3DAY\_STIMULATED\_IRF4\_KO\_BCELL\_DN |  | 26 | 0.35 | 1.61 | 0.041 | 1.000 | 1.000 | 625 | tags=69%, list=44%, signal=122% |
| 43 | GSE27786\_LIN\_NEG\_VS\_ERYTHROBLAST\_UP |  | 27 | 0.34 | 1.61 | 0.034 | 1.000 | 1.000 | 777 | tags=85%, list=55%, signal=186% |
| 44 | GSE13485\_CTRL\_VS\_DAY1\_YF17D\_VACCINE\_PBMC\_UP |  | 21 | 0.37 | 1.61 | 0.029 | 1.000 | 1.000 | 297 | tags=48%, list=21%, signal=59% |
| 45 | GSE14415\_INDUCED\_TREG\_VS\_FOXP3\_KO\_INDUCED\_TREG\_UP |  | 18 | 0.39 | 1.61 | 0.035 | 1.000 | 1.000 | 421 | tags=56%, list=30%, signal=78% |
| 46 | GSE29618\_PRE\_VS\_DAY7\_POST\_LAIV\_FLU\_VACCINE\_BCELL\_DN |  | 20 | 0.38 | 1.61 | 0.032 | 1.000 | 1.000 | 105 | tags=30%, list=7%, signal=32% |
| 47 | GSE19825\_NAIVE\_VS\_IL2RAHIGH\_DAY3\_EFF\_CD8\_TCELL\_DN |  | 17 | 0.41 | 1.60 | 0.052 | 1.000 | 1.000 | 127 | tags=35%, list=9%, signal=38% |
| 48 | GSE5542\_UNTREATED\_VS\_IFNG\_TREATED\_EPITHELIAL\_CELLS\_24H\_DN |  | 21 | 0.36 | 1.60 | 0.042 | 1.000 | 1.000 | 613 | tags=71%, list=43%, signal=124% |
| 49 | GSE9601\_NFKB\_INHIBITOR\_VS\_PI3K\_INHIBITOR\_TREATED\_HCMV\_INF\_MONOCYTE\_UP |  | 16 | 0.41 | 1.60 | 0.043 | 1.000 | 1.000 | 640 | tags=81%, list=45%, signal=147% |
| 50 | GSE24492\_LYVE\_NEG\_VS\_POS\_MACROPHAGE\_UP |  | 16 | 0.41 | 1.59 | 0.056 | 1.000 | 1.000 | 268 | tags=50%, list=19%, signal=61% |
| 51 | GSE26890\_CXCR1\_NEG\_VS\_POS\_EFFECTOR\_CD8\_TCELL\_UP |  | 23 | 0.35 | 1.59 | 0.057 | 1.000 | 1.000 | 282 | tags=43%, list=20%, signal=53% |
| 52 | GSE41867\_NAIVE\_VS\_EFFECTOR\_CD8\_TCELL\_DN |  | 21 | 0.36 | 1.59 | 0.046 | 1.000 | 1.000 | 763 | tags=90%, list=54%, signal=194% |
| 53 | GSE16450\_CTRL\_VS\_IFNA\_6H\_STIM\_IMMATURE\_NEURON\_CELL\_LINE\_UP |  | 21 | 0.36 | 1.58 | 0.045 | 1.000 | 1.000 | 324 | tags=48%, list=23%, signal=61% |
| 54 | GSE13485\_DAY1\_VS\_DAY21\_YF17D\_VACCINE\_PBMC\_DN |  | 18 | 0.39 | 1.58 | 0.040 | 1.000 | 1.000 | 600 | tags=72%, list=43%, signal=124% |
| 55 | GSE14908\_ATOPIC\_VS\_NONATOPIC\_PATIENT\_HDM\_STIM\_CD4\_TCELL\_UP |  | 18 | 0.39 | 1.58 | 0.040 | 1.000 | 1.000 | 299 | tags=44%, list=21%, signal=56% |
| 56 | GSE5503\_LIVER\_DC\_VS\_MLN\_DC\_ACTIVATED\_ALLOGENIC\_TCELL\_DN |  | 18 | 0.39 | 1.58 | 0.049 | 1.000 | 1.000 | 507 | tags=61%, list=36%, signal=94% |
| 57 | GSE27786\_LIN\_NEG\_VS\_NKCELL\_DN |  | 15 | 0.41 | 1.58 | 0.043 | 1.000 | 1.000 | 542 | tags=73%, list=38%, signal=118% |
| 58 | GSE16450\_CTRL\_VS\_IFNA\_12H\_STIM\_MATURE\_NEURON\_CELL\_LINE\_DN |  | 17 | 0.40 | 1.58 | 0.051 | 1.000 | 1.000 | 140 | tags=35%, list=10%, signal=39% |
| 59 | GSE14000\_TRANSLATED\_RNA\_VS\_MRNA\_DC\_UP |  | 23 | 0.36 | 1.57 | 0.036 | 1.000 | 1.000 | 616 | tags=78%, list=44%, signal=137% |
| 60 | GSE2770\_IL12\_ACT\_VS\_ACT\_CD4\_TCELL\_2H\_DN |  | 30 | 0.33 | 1.56 | 0.045 | 1.000 | 1.000 | 254 | tags=37%, list=18%, signal=44% |
| 61 | GSE41867\_NAIVE\_VS\_DAY8\_LCMV\_ARMSTRONG\_EFFECTOR\_CD8\_TCELL\_UP |  | 15 | 0.42 | 1.56 | 0.049 | 1.000 | 1.000 | 282 | tags=53%, list=20%, signal=66% |
| 62 | GSE20727\_DNFB\_ALLERGEN\_VS\_ROS\_INH\_AND\_DNFB\_ALLERGEN\_TREATED\_DC\_UP |  | 16 | 0.40 | 1.55 | 0.058 | 1.000 | 1.000 | 160 | tags=38%, list=11%, signal=42% |
| 63 | GSE14769\_UNSTIM\_VS\_120MIN\_LPS\_BMDM\_UP |  | 26 | 0.34 | 1.55 | 0.057 | 1.000 | 1.000 | 691 | tags=81%, list=49%, signal=155% |
| 64 | GSE12963\_ENV\_NEF\_VS\_ENV\_NEF\_AND\_VPR\_DEFICIENT\_HIV1\_INF\_CD4\_TCELL\_UP |  | 17 | 0.38 | 1.54 | 0.039 | 1.000 | 1.000 | 229 | tags=41%, list=16%, signal=49% |
| 65 | GSE18203\_CTRL\_VS\_INTRATUMORAL\_CPG\_INJ\_MC38\_TUMOR\_UP |  | 15 | 0.40 | 1.54 | 0.067 | 1.000 | 1.000 | 265 | tags=53%, list=19%, signal=65% |
| 66 | GSE27786\_LIN\_NEG\_VS\_MONO\_MAC\_UP |  | 27 | 0.33 | 1.54 | 0.047 | 1.000 | 1.000 | 695 | tags=78%, list=49%, signal=150% |
| 67 | GSE17721\_LPS\_VS\_PAM3CSK4\_6H\_BMDC\_DN |  | 18 | 0.38 | 1.53 | 0.054 | 1.000 | 1.000 | 561 | tags=72%, list=40%, signal=118% |
| 68 | GSE15659\_CD45RA\_NEG\_CD4\_TCELL\_VS\_RESTING\_TREG\_DN |  | 18 | 0.37 | 1.53 | 0.047 | 1.000 | 1.000 | 465 | tags=61%, list=33%, signal=90% |
| 69 | GSE41867\_NAIVE\_VS\_DAY30\_LCMV\_ARMSTRONG\_MEMORY\_CD8\_TCELL\_DN |  | 24 | 0.34 | 1.53 | 0.062 | 1.000 | 1.000 | 818 | tags=92%, list=58%, signal=214% |
| 70 | GSE2405\_0H\_VS\_9H\_A\_PHAGOCYTOPHILUM\_STIM\_NEUTROPHIL\_DN |  | 16 | 0.40 | 1.53 | 0.064 | 1.000 | 1.000 | 215 | tags=31%, list=15%, signal=36% |
| 71 | GSE40685\_NAIVE\_CD4\_TCELL\_VS\_TREG\_UP |  | 18 | 0.37 | 1.52 | 0.060 | 1.000 | 1.000 | 728 | tags=89%, list=52%, signal=181% |
| 72 | GSE13411\_NAIVE\_VS\_MEMORY\_BCELL\_DN |  | 19 | 0.35 | 1.52 | 0.072 | 1.000 | 1.000 | 494 | tags=58%, list=35%, signal=88% |
| 73 | GSE41867\_DAY8\_EFFECTOR\_VS\_DAY30\_MEMORY\_CD8\_TCELL\_LCMV\_ARMSTRONG\_UP |  | 26 | 0.33 | 1.51 | 0.069 | 1.000 | 1.000 | 812 | tags=92%, list=58%, signal=213% |
| 74 | GSE7509\_UNSTIM\_VS\_TNFA\_IL1B\_IL6\_PGE\_STIM\_DC\_UP |  | 23 | 0.34 | 1.51 | 0.055 | 1.000 | 1.000 | 798 | tags=91%, list=57%, signal=207% |
| 75 | GSE6092\_IFNG\_VS\_IFNG\_AND\_B\_BURGDORFERI\_INF\_ENDOTHELIAL\_CELL\_DN |  | 16 | 0.39 | 1.51 | 0.078 | 1.000 | 1.000 | 161 | tags=38%, list=11%, signal=42% |
| 76 | GSE37532\_TREG\_VS\_TCONV\_CD4\_TCELL\_FROM\_VISCERAL\_ADIPOSE\_TISSUE\_DN |  | 19 | 0.37 | 1.50 | 0.058 | 1.000 | 1.000 | 666 | tags=79%, list=47%, signal=148% |
| 77 | GSE8921\_UNSTIM\_0H\_VS\_TLR1\_2\_STIM\_MONOCYTE\_24H\_DN |  | 20 | 0.35 | 1.50 | 0.071 | 1.000 | 1.000 | 207 | tags=40%, list=15%, signal=46% |
| 78 | GSE13485\_DAY1\_VS\_DAY3\_YF17D\_VACCINE\_PBMC\_DN |  | 29 | 0.31 | 1.50 | 0.068 | 1.000 | 1.000 | 378 | tags=48%, list=27%, signal=65% |
| 79 | GSE21678\_WT\_VS\_FOXO1\_FOXO3\_KO\_TREG\_DN |  | 28 | 0.32 | 1.49 | 0.080 | 1.000 | 1.000 | 648 | tags=71%, list=46%, signal=129% |
| 80 | GSE3982\_NEUTROPHIL\_VS\_NKCELL\_DN |  | 20 | 0.36 | 1.49 | 0.083 | 1.000 | 1.000 | 810 | tags=90%, list=57%, signal=208% |
| 81 | GSE6259\_FLT3L\_INDUCED\_33D1\_POS\_DC\_VS\_BCELL\_UP |  | 22 | 0.34 | 1.49 | 0.075 | 1.000 | 1.000 | 815 | tags=91%, list=58%, signal=212% |
| 82 | GSE21546\_WT\_VS\_SAP1A\_KO\_ANTI\_CD3\_STIM\_DP\_THYMOCYTES\_DN |  | 30 | 0.30 | 1.49 | 0.069 | 1.000 | 1.000 | 498 | tags=57%, list=35%, signal=86% |
| 83 | GSE15930\_STIM\_VS\_STIM\_AND\_IFNAB\_48H\_CD8\_T\_CELL\_DN |  | 22 | 0.34 | 1.49 | 0.083 | 1.000 | 1.000 | 289 | tags=36%, list=20%, signal=45% |
| 84 | GSE29618\_LAIV\_VS\_TIV\_FLU\_VACCINE\_DAY7\_BCELL\_DN |  | 19 | 0.36 | 1.49 | 0.065 | 1.000 | 1.000 | 555 | tags=68%, list=39%, signal=111% |
| 85 | GSE21927\_SPLENIC\_C26GM\_TUMOROUS\_VS\_4T1\_TUMOR\_MONOCYTES\_DN |  | 26 | 0.32 | 1.49 | 0.067 | 1.000 | 1.000 | 382 | tags=50%, list=27%, signal=67% |
| 86 | GSE18893\_TCONV\_VS\_TREG\_2H\_CULTURE\_UP |  | 20 | 0.34 | 1.49 | 0.088 | 1.000 | 1.000 | 476 | tags=60%, list=34%, signal=89% |
| 87 | GSE43863\_NAIVE\_VS\_LY6C\_INT\_CXCR5POS\_CD4\_EFF\_TCELL\_D6\_LCMV\_UP |  | 15 | 0.39 | 1.48 | 0.070 | 1.000 | 1.000 | 305 | tags=53%, list=22%, signal=67% |
| 88 | GSE35825\_UNTREATED\_VS\_IFNA\_STIM\_MACROPHAGE\_DN |  | 17 | 0.36 | 1.48 | 0.095 | 1.000 | 1.000 | 124 | tags=29%, list=9%, signal=32% |
| 89 | GSE14308\_TH2\_VS\_NAIVE\_CD4\_TCELL\_DN |  | 19 | 0.35 | 1.48 | 0.066 | 1.000 | 1.000 | 618 | tags=79%, list=44%, signal=139% |
| 90 | GSE7852\_THYMUS\_VS\_FAT\_TCONV\_UP |  | 17 | 0.37 | 1.47 | 0.086 | 1.000 | 1.000 | 407 | tags=53%, list=29%, signal=74% |
| 91 | GSE12198\_LOW\_IL2\_STIM\_NK\_CELL\_VS\_HIGH\_IL2\_STIM\_NK\_CELL\_UP |  | 17 | 0.36 | 1.46 | 0.088 | 1.000 | 1.000 | 421 | tags=53%, list=30%, signal=75% |
| 92 | GSE27786\_LSK\_VS\_NEUTROPHIL\_UP |  | 22 | 0.33 | 1.46 | 0.090 | 1.000 | 1.000 | 724 | tags=77%, list=51%, signal=156% |
| 93 | GSE15330\_GRANULOCYTE\_MONOCYTE\_PROGENITOR\_VS\_PRO\_BCELL\_UP |  | 15 | 0.39 | 1.45 | 0.087 | 1.000 | 1.000 | 403 | tags=53%, list=29%, signal=74% |
| 94 | GSE3982\_EOSINOPHIL\_VS\_MAST\_CELL\_UP |  | 24 | 0.31 | 1.45 | 0.076 | 1.000 | 1.000 | 439 | tags=54%, list=31%, signal=77% |
| 95 | GSE11864\_CSF1\_VS\_CSF1\_IFNG\_PAM3CYS\_IN\_MAC\_UP |  | 16 | 0.37 | 1.45 | 0.080 | 1.000 | 1.000 | 50 | tags=25%, list=4%, signal=26% |
| 96 | GSE15750\_WT\_VS\_TRAF6KO\_DAY6\_EFF\_CD8\_TCELL\_DN |  | 15 | 0.39 | 1.45 | 0.089 | 1.000 | 1.000 | 91 | tags=27%, list=6%, signal=28% |
| 97 | GSE36078\_UNTREATED\_VS\_AD5\_T425A\_HEXON\_INF\_MOUSE\_LUNG\_DC\_DN |  | 21 | 0.33 | 1.45 | 0.091 | 1.000 | 1.000 | 389 | tags=48%, list=28%, signal=65% |
| 98 | GSE17721\_CTRL\_VS\_LPS\_2H\_BMDC\_UP |  | 22 | 0.34 | 1.44 | 0.104 | 1.000 | 1.000 | 717 | tags=82%, list=51%, signal=164% |
| 99 | GSE29618\_LAIV\_VS\_TIV\_FLU\_VACCINE\_DAY7\_MONOCYTE\_UP |  | 25 | 0.31 | 1.44 | 0.095 | 1.000 | 1.000 | 588 | tags=68%, list=42%, signal=115% |
| 100 | GSE20500\_CTRL\_VS\_RETINOIC\_ACID\_TREATED\_CD4\_TCELL\_UP |  | 17 | 0.36 | 1.44 | 0.105 | 1.000 | 1.000 | 810 | tags=94%, list=57%, signal=218% |
| 101 | GSE16385\_IFNG\_TNF\_VS\_ROSIGLITAZONE\_STIM\_MACROPHAGE\_UP |  | 16 | 0.36 | 1.44 | 0.096 | 1.000 | 1.000 | 556 | tags=75%, list=39%, signal=122% |
| 102 | GSE45365\_HEALTHY\_VS\_MCMV\_INFECTION\_CD11B\_DC\_UP |  | 22 | 0.32 | 1.44 | 0.095 | 1.000 | 1.000 | 535 | tags=64%, list=38%, signal=101% |
| 103 | GSE24726\_WT\_VS\_E2\_2\_KO\_PDC\_DAY6\_POST\_DELETION\_UP |  | 19 | 0.35 | 1.44 | 0.093 | 1.000 | 1.000 | 254 | tags=37%, list=18%, signal=44% |
| 104 | GSE2770\_IL12\_ACT\_VS\_ACT\_CD4\_TCELL\_6H\_DN |  | 17 | 0.36 | 1.43 | 0.108 | 1.000 | 1.000 | 715 | tags=82%, list=51%, signal=165% |
| 105 | GSE37416\_CTRL\_VS\_48H\_F\_TULARENSIS\_LVS\_NEUTROPHIL\_UP |  | 21 | 0.34 | 1.43 | 0.088 | 1.000 | 1.000 | 644 | tags=76%, list=46%, signal=138% |
| 106 | GSE22432\_MULTIPOTENT\_VS\_COMMON\_DC\_PROGENITOR\_UNTREATED\_DN |  | 34 | 0.28 | 1.43 | 0.082 | 1.000 | 1.000 | 847 | tags=85%, list=60%, signal=208% |
| 107 | GSE27786\_BCELL\_VS\_ERYTHROBLAST\_UP |  | 23 | 0.33 | 1.43 | 0.098 | 1.000 | 1.000 | 28 | tags=17%, list=2%, signal=17% |
| 108 | GSE21546\_ELK1\_KO\_VS\_SAP1A\_KO\_AND\_ELK1\_KO\_ANTI\_CD3\_STIM\_DP\_THYMOCYTES\_UP |  | 20 | 0.34 | 1.42 | 0.090 | 1.000 | 1.000 | 705 | tags=80%, list=50%, signal=158% |
| 109 | GSE22886\_NAIVE\_TCELL\_VS\_NEUTROPHIL\_UP |  | 22 | 0.32 | 1.42 | 0.096 | 1.000 | 1.000 | 389 | tags=45%, list=28%, signal=62% |
| 110 | GSE25146\_UNSTIM\_VS\_HELIOBACTER\_PYLORI\_LPS\_STIM\_AGS\_CELL\_UP |  | 17 | 0.35 | 1.42 | 0.115 | 1.000 | 1.000 | 755 | tags=88%, list=54%, signal=188% |
| 111 | GSE10273\_LOW\_IL7\_VS\_HIGH\_IL7\_AND\_IRF4\_IN\_IRF4\_8\_NULL\_PRE\_BCELL\_DN |  | 19 | 0.35 | 1.42 | 0.106 | 1.000 | 1.000 | 415 | tags=53%, list=29%, signal=74% |
| 112 | GSE28449\_WT\_VS\_LRF\_KO\_GERMINAL\_CENTER\_BCELL\_DN |  | 17 | 0.35 | 1.42 | 0.091 | 1.000 | 1.000 | 405 | tags=53%, list=29%, signal=73% |
| 113 | GSE1460\_INTRATHYMIC\_T\_PROGENITOR\_VS\_THYMIC\_STROMAL\_CELL\_UP |  | 19 | 0.34 | 1.42 | 0.115 | 1.000 | 1.000 | 556 | tags=63%, list=39%, signal=103% |
| 114 | GSE24574\_NAIVE\_VS\_TCONV\_CD4\_TCELL\_DN |  | 32 | 0.29 | 1.42 | 0.093 | 1.000 | 1.000 | 878 | tags=91%, list=62%, signal=234% |
| 115 | GSE44649\_NAIVE\_VS\_ACTIVATED\_CD8\_TCELL\_MIR155\_KO\_DN |  | 25 | 0.31 | 1.41 | 0.102 | 1.000 | 1.000 | 379 | tags=48%, list=27%, signal=64% |
| 116 | GSE21546\_UNSTIM\_VS\_ANTI\_CD3\_STIM\_DP\_THYMOCYTES\_DN |  | 26 | 0.31 | 1.41 | 0.086 | 1.000 | 1.000 | 818 | tags=88%, list=58%, signal=207% |
| 117 | GSE14769\_UNSTIM\_VS\_120MIN\_LPS\_BMDM\_DN |  | 32 | 0.29 | 1.41 | 0.076 | 1.000 | 1.000 | 23 | tags=16%, list=2%, signal=16% |
| 118 | GSE22886\_IGA\_VS\_IGM\_MEMORY\_BCELL\_DN |  | 21 | 0.33 | 1.41 | 0.120 | 1.000 | 1.000 | 337 | tags=48%, list=24%, signal=62% |
| 119 | GSE18791\_UNSTIM\_VS\_NEWCATSLE\_VIRUS\_DC\_10H\_UP |  | 27 | 0.30 | 1.40 | 0.106 | 1.000 | 1.000 | 681 | tags=74%, list=48%, signal=140% |
| 120 | GSE7509\_UNSTIM\_VS\_IFNA\_STIM\_IMMATURE\_DC\_UP |  | 17 | 0.35 | 1.40 | 0.116 | 1.000 | 1.000 | 587 | tags=65%, list=42%, signal=109% |
| 121 | GSE369\_IFNG\_KO\_VS\_WT\_LIVER\_DN |  | 25 | 0.30 | 1.40 | 0.108 | 1.000 | 1.000 | 815 | tags=88%, list=58%, signal=205% |
| 122 | GSE40274\_HELIOS\_VS\_FOXP3\_AND\_HELIOS\_TRANSDUCED\_ACTIVATED\_CD4\_TCELL\_DN |  | 17 | 0.35 | 1.40 | 0.115 | 1.000 | 1.000 | 295 | tags=47%, list=21%, signal=59% |
| 123 | GSE20366\_CD103\_POS\_VS\_CD103\_KLRG1\_DP\_TREG\_UP |  | 21 | 0.32 | 1.40 | 0.107 | 1.000 | 1.000 | 690 | tags=76%, list=49%, signal=147% |
| 124 | GSE7852\_THYMUS\_VS\_FAT\_TREG\_UP |  | 19 | 0.34 | 1.40 | 0.108 | 1.000 | 1.000 | 513 | tags=63%, list=36%, signal=98% |
| 125 | GSE25123\_ROSIGLITAZONE\_VS\_IL4\_AND\_ROSIGLITAZONE\_STIM\_MACROPHAGE\_DAY10\_UP |  | 21 | 0.32 | 1.39 | 0.101 | 1.000 | 1.000 | 362 | tags=48%, list=26%, signal=63% |
| 126 | GSE8621\_LPS\_PRIMED\_UNSTIM\_VS\_LPS\_PRIMED\_AND\_LPS\_STIM\_MACROPHAGE\_DN |  | 24 | 0.31 | 1.39 | 0.109 | 1.000 | 1.000 | 429 | tags=54%, list=30%, signal=77% |
| 127 | GSE17721\_LPS\_VS\_GARDIQUIMOD\_4H\_BMDC\_DN |  | 16 | 0.36 | 1.39 | 0.120 | 1.000 | 1.000 | 476 | tags=63%, list=34%, signal=93% |
| 128 | GSE3982\_EOSINOPHIL\_VS\_MAST\_CELL\_DN |  | 24 | 0.31 | 1.39 | 0.113 | 1.000 | 1.000 | 626 | tags=71%, list=44%, signal=125% |
| 129 | GSE22886\_NAIVE\_TCELL\_VS\_DC\_DN |  | 18 | 0.34 | 1.39 | 0.115 | 1.000 | 1.000 | 327 | tags=44%, list=23%, signal=57% |
| 130 | GSE21063\_CTRL\_VS\_ANTI\_IGM\_STIM\_BCELL\_16H\_DN |  | 20 | 0.33 | 1.39 | 0.113 | 1.000 | 1.000 | 554 | tags=65%, list=39%, signal=106% |
| 131 | GSE44649\_NAIVE\_VS\_ACTIVATED\_CD8\_TCELL\_DN |  | 22 | 0.32 | 1.38 | 0.128 | 1.000 | 1.000 | 285 | tags=41%, list=20%, signal=50% |
| 132 | GSE21379\_TFH\_VS\_NON\_TFH\_CD4\_TCELL\_UP |  | 20 | 0.32 | 1.38 | 0.098 | 1.000 | 1.000 | 93 | tags=25%, list=7%, signal=26% |
| 133 | GSE2128\_CTRL\_VS\_MIMETOPE\_NEGATIVE\_SELECTION\_DP\_THYMOCYTE\_C57BL6\_DN |  | 24 | 0.31 | 1.38 | 0.101 | 1.000 | 1.000 | 222 | tags=33%, list=16%, signal=39% |
| 134 | GSE20727\_CTRL\_VS\_H2O2\_TREATED\_DC\_DN |  | 23 | 0.30 | 1.38 | 0.115 | 1.000 | 1.000 | 140 | tags=26%, list=10%, signal=28% |
| 135 | GSE10147\_IL3\_AND\_HIVP17\_VS\_IL3\_AND\_CPG\_STIM\_PDC\_DN |  | 19 | 0.33 | 1.38 | 0.141 | 1.000 | 1.000 | 53 | tags=21%, list=4%, signal=22% |
| 136 | GSE3982\_CTRL\_VS\_IGE\_STIM\_MAST\_CELL\_UP |  | 17 | 0.34 | 1.38 | 0.120 | 1.000 | 1.000 | 604 | tags=71%, list=43%, signal=122% |
| 137 | GSE5542\_IFNG\_VS\_IFNA\_TREATED\_EPITHELIAL\_CELLS\_6H\_UP |  | 21 | 0.32 | 1.38 | 0.122 | 1.000 | 1.000 | 636 | tags=71%, list=45%, signal=128% |
| 138 | GSE17721\_LPS\_VS\_POLYIC\_4H\_BMDC\_UP |  | 20 | 0.32 | 1.38 | 0.123 | 1.000 | 1.000 | 425 | tags=55%, list=30%, signal=78% |
| 139 | GSE4590\_PRE\_BCELL\_VS\_VPREB\_POS\_LARGE\_PRE\_BCELL\_DN |  | 18 | 0.33 | 1.38 | 0.123 | 1.000 | 1.000 | 543 | tags=61%, list=38%, signal=98% |
| 140 | GSE21546\_WT\_VS\_ELK1\_KO\_ANTI\_CD3\_STIM\_DP\_THYMOCYTES\_DN |  | 23 | 0.31 | 1.37 | 0.112 | 1.000 | 1.000 | 553 | tags=65%, list=39%, signal=106% |
| 141 | GSE17721\_CTRL\_VS\_LPS\_24H\_BMDC\_DN |  | 22 | 0.31 | 1.37 | 0.123 | 1.000 | 1.000 | 715 | tags=73%, list=51%, signal=145% |
| 142 | GSE3982\_NEUTROPHIL\_VS\_TH1\_DN |  | 21 | 0.32 | 1.37 | 0.124 | 1.000 | 1.000 | 870 | tags=95%, list=62%, signal=245% |
| 143 | GSE7764\_IL15\_NK\_CELL\_24H\_VS\_SPLENOCYTE\_UP |  | 22 | 0.31 | 1.37 | 0.123 | 1.000 | 1.000 | 362 | tags=45%, list=26%, signal=60% |
| 144 | GSE46606\_IRF4\_KO\_VS\_WT\_CD40L\_IL2\_IL5\_3DAY\_STIMULATED\_BCELL\_DN |  | 16 | 0.35 | 1.37 | 0.131 | 1.000 | 1.000 | 705 | tags=88%, list=50%, signal=173% |
| 145 | GSE2770\_UNTREATED\_VS\_IL12\_TREATED\_ACT\_CD4\_TCELL\_48H\_DN |  | 19 | 0.33 | 1.37 | 0.141 | 1.000 | 1.000 | 957 | tags=100%, list=68%, signal=307% |
| 146 | GSE40666\_STAT1\_KO\_VS\_STAT4\_KO\_CD8\_TCELL\_WITH\_IFNA\_STIM\_90MIN\_UP |  | 15 | 0.36 | 1.37 | 0.131 | 1.000 | 1.000 | 274 | tags=40%, list=19%, signal=49% |
| 147 | GSE3982\_DC\_VS\_NEUTROPHIL\_LPS\_STIM\_UP |  | 19 | 0.33 | 1.36 | 0.140 | 1.000 | 1.000 | 749 | tags=84%, list=53%, signal=177% |
| 148 | GSE17721\_CTRL\_VS\_CPG\_12H\_BMDC\_DN |  | 20 | 0.33 | 1.36 | 0.125 | 1.000 | 1.000 | 95 | tags=25%, list=7%, signal=26% |
| 149 | GSE23321\_CD8\_STEM\_CELL\_MEMORY\_VS\_CENTRAL\_MEMORY\_CD8\_TCELL\_DN |  | 19 | 0.32 | 1.36 | 0.124 | 1.000 | 1.000 | 211 | tags=32%, list=15%, signal=37% |
| 150 | GSE6674\_UNSTIM\_VS\_ANTI\_IGM\_STIM\_BCELL\_UP |  | 16 | 0.35 | 1.36 | 0.130 | 1.000 | 1.000 | 245 | tags=38%, list=17%, signal=45% |
| 151 | GSE37532\_WT\_VS\_PPARG\_KO\_LN\_TCONV\_DN |  | 16 | 0.35 | 1.36 | 0.129 | 1.000 | 1.000 | 232 | tags=38%, list=16%, signal=44% |
| 152 | GSE7218\_UNSTIM\_VS\_ANTIGEN\_STIM\_THROUGH\_IGG\_BCELL\_UP |  | 18 | 0.33 | 1.35 | 0.130 | 1.000 | 1.000 | 696 | tags=78%, list=49%, signal=152% |
| 153 | GSE29615\_CTRL\_VS\_DAY3\_LAIV\_IFLU\_VACCINE\_PBMC\_DN |  | 18 | 0.33 | 1.35 | 0.125 | 1.000 | 1.000 | 613 | tags=72%, list=43%, signal=126% |
| 154 | GSE13547\_WT\_VS\_ZFX\_KO\_BCELL\_ANTI\_IGM\_STIM\_2H\_UP |  | 22 | 0.31 | 1.35 | 0.144 | 1.000 | 1.000 | 372 | tags=45%, list=26%, signal=61% |
| 155 | GSE37533\_PPARG1\_FOXP3\_VS\_PPARG2\_FOXP3\_TRANSDUCED\_CD4\_TCELL\_UP |  | 21 | 0.32 | 1.35 | 0.135 | 1.000 | 1.000 | 631 | tags=71%, list=45%, signal=127% |
| 156 | GSE32423\_MEMORY\_VS\_NAIVE\_CD8\_TCELL\_IL7\_DN |  | 23 | 0.30 | 1.35 | 0.131 | 1.000 | 1.000 | 720 | tags=74%, list=51%, signal=148% |
| 157 | GSE34205\_HEALTHY\_VS\_RSV\_INF\_INFANT\_PBMC\_UP |  | 17 | 0.33 | 1.35 | 0.142 | 1.000 | 1.000 | 293 | tags=41%, list=21%, signal=51% |
| 158 | GSE18791\_CTRL\_VS\_NEWCASTLE\_VIRUS\_DC\_14H\_UP |  | 35 | 0.26 | 1.35 | 0.130 | 1.000 | 1.000 | 701 | tags=74%, list=50%, signal=144% |
| 159 | GSE27786\_CD8\_TCELL\_VS\_ERYTHROBLAST\_UP |  | 19 | 0.32 | 1.35 | 0.150 | 1.000 | 1.000 | 486 | tags=53%, list=34%, signal=79% |
| 160 | GSE14908\_RESTING\_VS\_HDM\_STIM\_CD4\_TCELL\_ATOPIC\_PATIENT\_DN |  | 18 | 0.32 | 1.35 | 0.139 | 1.000 | 1.000 | 162 | tags=28%, list=11%, signal=31% |
| 161 | GSE27786\_LSK\_VS\_ERYTHROBLAST\_UP |  | 19 | 0.32 | 1.35 | 0.131 | 1.000 | 1.000 | 715 | tags=79%, list=51%, signal=158% |
| 162 | GSE4142\_NAIVE\_VS\_GC\_BCELL\_DN |  | 25 | 0.30 | 1.35 | 0.133 | 1.000 | 1.000 | 269 | tags=36%, list=19%, signal=44% |
| 163 | GSE11924\_TH1\_VS\_TH2\_CD4\_TCELL\_UP |  | 15 | 0.36 | 1.34 | 0.138 | 1.000 | 1.000 | 910 | tags=100%, list=64%, signal=279% |
| 164 | GSE17721\_CPG\_VS\_GARDIQUIMOD\_8H\_BMDC\_DN |  | 21 | 0.31 | 1.34 | 0.123 | 1.000 | 1.000 | 455 | tags=57%, list=32%, signal=83% |
| 165 | GSE21033\_CTRL\_VS\_POLYIC\_STIM\_DC\_3H\_DN |  | 20 | 0.32 | 1.34 | 0.151 | 1.000 | 1.000 | 744 | tags=80%, list=53%, signal=167% |
| 166 | GSE2770\_TGFB\_AND\_IL4\_ACT\_VS\_ACT\_CD4\_TCELL\_6H\_UP |  | 16 | 0.35 | 1.34 | 0.146 | 1.000 | 1.000 | 164 | tags=31%, list=12%, signal=35% |
| 167 | GSE12963\_UNINF\_VS\_ENV\_AND\_NEF\_AND\_VPR\_DEFICIENT\_HIV1\_INF\_CD4\_TCELL\_UP |  | 16 | 0.35 | 1.33 | 0.148 | 1.000 | 1.000 | 600 | tags=69%, list=43%, signal=118% |
| 168 | GSE7764\_NKCELL\_VS\_SPLENOCYTE\_UP |  | 19 | 0.32 | 1.33 | 0.151 | 1.000 | 1.000 | 170 | tags=32%, list=12%, signal=35% |
| 169 | GSE36888\_UNTREATED\_VS\_IL2\_TREATED\_TCELL\_6H\_DN |  | 19 | 0.32 | 1.33 | 0.144 | 1.000 | 1.000 | 230 | tags=37%, list=16%, signal=43% |
| 170 | GSE18791\_CTRL\_VS\_NEWCASTLE\_VIRUS\_DC\_18H\_UP |  | 33 | 0.26 | 1.33 | 0.144 | 1.000 | 1.000 | 733 | tags=76%, list=52%, signal=154% |
| 171 | GSE7400\_CTRL\_VS\_CSF3\_IN\_VIVO\_TREATED\_PBMC\_UP |  | 29 | 0.28 | 1.33 | 0.151 | 1.000 | 1.000 | 620 | tags=66%, list=44%, signal=114% |
| 172 | GSE24634\_TREG\_VS\_TCONV\_POST\_DAY5\_IL4\_CONVERSION\_UP |  | 30 | 0.27 | 1.33 | 0.144 | 1.000 | 1.000 | 646 | tags=63%, list=46%, signal=114% |
| 173 | GSE43863\_TH1\_VS\_LY6C\_LOW\_CXCR5NEG\_EFFECTOR\_CD4\_TCELL\_UP |  | 16 | 0.34 | 1.33 | 0.158 | 1.000 | 1.000 | 230 | tags=31%, list=16%, signal=37% |
| 174 | GSE29614\_DAY3\_VS\_DAY7\_TIV\_FLU\_VACCINE\_PBMC\_DN |  | 15 | 0.34 | 1.33 | 0.170 | 1.000 | 1.000 | 13 | tags=20%, list=1%, signal=20% |
| 175 | GSE6092\_CTRL\_VS\_BORRELIA\_BIRGDOFERI\_INF\_ENDOTHELIAL\_CELL\_UP |  | 15 | 0.35 | 1.33 | 0.155 | 1.000 | 1.000 | 747 | tags=87%, list=53%, signal=182% |
| 176 | GSE29618\_PRE\_VS\_DAY7\_FLU\_VACCINE\_PDC\_DN |  | 26 | 0.28 | 1.32 | 0.154 | 1.000 | 1.000 | 20 | tags=15%, list=1%, signal=15% |
| 177 | GSE411\_UNSTIM\_VS\_400MIN\_IL6\_STIM\_SOCS3\_KO\_MACROPHAGE\_UP |  | 15 | 0.35 | 1.32 | 0.151 | 1.000 | 1.000 | 297 | tags=40%, list=21%, signal=50% |
| 178 | GSE40225\_WT\_VS\_RIP\_B7X\_DIABETIC\_MOUSE\_PANCREATIC\_CD8\_TCELL\_UP |  | 20 | 0.31 | 1.32 | 0.159 | 1.000 | 1.000 | 395 | tags=50%, list=28%, signal=68% |
| 179 | GSE15330\_HSC\_VS\_GRANULOCYTE\_MONOCYTE\_PROGENITOR\_UP |  | 28 | 0.27 | 1.32 | 0.143 | 1.000 | 1.000 | 403 | tags=46%, list=29%, signal=64% |
| 180 | GSE1791\_CTRL\_VS\_NEUROMEDINU\_IN\_T\_CELL\_LINE\_6H\_DN |  | 21 | 0.31 | 1.32 | 0.151 | 1.000 | 1.000 | 349 | tags=43%, list=25%, signal=56% |
| 181 | GSE11864\_UNTREATED\_VS\_CSF1\_IN\_MAC\_DN |  | 19 | 0.32 | 1.32 | 0.154 | 1.000 | 1.000 | 344 | tags=47%, list=24%, signal=62% |
| 182 | GSE41867\_NAIVE\_VS\_DAY15\_LCMV\_EFFECTOR\_CD8\_TCELL\_UP |  | 22 | 0.31 | 1.32 | 0.159 | 1.000 | 1.000 | 593 | tags=68%, list=42%, signal=116% |
| 183 | GSE3982\_CTRL\_VS\_IGE\_STIM\_MAST\_CELL\_DN |  | 19 | 0.32 | 1.32 | 0.160 | 1.000 | 1.000 | 76 | tags=21%, list=5%, signal=22% |
| 184 | GSE22611\_UNSTIM\_VS\_6H\_MDP\_STIM\_MUTANT\_NOD2\_TRANSDUCED\_HEK293T\_CELL\_DN |  | 15 | 0.35 | 1.31 | 0.173 | 1.000 | 1.000 | 439 | tags=53%, list=31%, signal=77% |
| 185 | GSE41867\_DAY8\_EFFECTOR\_VS\_DAY30\_MEMORY\_CD8\_TCELL\_LCMV\_ARMSTRONG\_DN |  | 20 | 0.31 | 1.31 | 0.157 | 1.000 | 1.000 | 611 | tags=75%, list=43%, signal=130% |
| 186 | GSE19374\_UNINF\_VS\_LISTERIA\_INFECTED\_MACROPHAGE\_UP |  | 16 | 0.34 | 1.31 | 0.170 | 1.000 | 1.000 | 439 | tags=50%, list=31%, signal=72% |
| 187 | GSE9006\_HEALTHY\_VS\_TYPE\_1\_DIABETES\_PBMC\_4MONTH\_POST\_DX\_DN |  | 20 | 0.31 | 1.31 | 0.164 | 1.000 | 1.000 | 695 | tags=75%, list=49%, signal=146% |
| 188 | GSE41867\_NAIVE\_VS\_DAY15\_LCMV\_ARMSTRONG\_EFFECTOR\_CD8\_TCELL\_UP |  | 21 | 0.30 | 1.31 | 0.162 | 1.000 | 1.000 | 473 | tags=57%, list=34%, signal=85% |
| 189 | GSE6259\_DEC205\_POS\_DC\_VS\_CD8\_TCELL\_UP |  | 15 | 0.34 | 1.31 | 0.179 | 1.000 | 1.000 | 67 | tags=20%, list=5%, signal=21% |
| 190 | GSE29617\_CTRL\_VS\_TIV\_FLU\_VACCINE\_PBMC\_2008\_DN |  | 28 | 0.27 | 1.31 | 0.162 | 1.000 | 1.000 | 576 | tags=57%, list=41%, signal=95% |
| 191 | GSE37532\_WT\_VS\_PPARG\_KO\_VISCERAL\_ADIPOSE\_TISSUE\_TREG\_UP |  | 16 | 0.34 | 1.31 | 0.175 | 1.000 | 1.000 | 727 | tags=81%, list=52%, signal=166% |
| 192 | GSE37416\_0H\_VS\_3H\_F\_TULARENSIS\_LVS\_NEUTROPHIL\_UP |  | 18 | 0.31 | 1.30 | 0.179 | 1.000 | 1.000 | 181 | tags=33%, list=13%, signal=38% |
| 193 | GSE11386\_NAIVE\_VS\_MEMORY\_BCELL\_DN |  | 23 | 0.29 | 1.30 | 0.155 | 1.000 | 1.000 | 73 | tags=22%, list=5%, signal=23% |
| 194 | GSE2770\_UNTREATED\_VS\_TGFB\_AND\_IL4\_TREATED\_ACT\_CD4\_TCELL\_2H\_UP |  | 20 | 0.30 | 1.29 | 0.184 | 1.000 | 1.000 | 68 | tags=20%, list=5%, signal=21% |
| 195 | GSE19888\_ADENOSINE\_A3R\_INH\_PRETREAT\_AND\_ACT\_BY\_A3R\_VS\_TCELL\_MEMBRANES\_ACT\_MAST\_CELL\_DN |  | 26 | 0.28 | 1.29 | 0.164 | 1.000 | 1.000 | 331 | tags=38%, list=23%, signal=49% |
| 196 | GSE1460\_DP\_THYMOCYTE\_VS\_NAIVE\_CD4\_TCELL\_ADULT\_BLOOD\_UP |  | 15 | 0.34 | 1.29 | 0.170 | 1.000 | 1.000 | 841 | tags=93%, list=60%, signal=229% |
| 197 | GSE18791\_CTRL\_VS\_NEWCASTLE\_VIRUS\_DC\_12H\_UP |  | 33 | 0.26 | 1.29 | 0.164 | 1.000 | 1.000 | 563 | tags=61%, list=40%, signal=98% |
| 198 | GSE33424\_CD161\_INT\_VS\_NEG\_CD8\_TCELL\_UP |  | 22 | 0.29 | 1.29 | 0.173 | 1.000 | 1.000 | 263 | tags=36%, list=19%, signal=44% |
| 199 | GSE17721\_PAM3CSK4\_VS\_CPG\_4H\_BMDC\_UP |  | 18 | 0.32 | 1.29 | 0.166 | 1.000 | 1.000 | 243 | tags=33%, list=17%, signal=40% |
| 200 | GSE7852\_LN\_VS\_FAT\_TREG\_UP |  | 23 | 0.28 | 1.29 | 0.186 | 1.000 | 1.000 | 515 | tags=52%, list=36%, signal=81% |
| 201 | GSE17721\_POLYIC\_VS\_CPG\_2H\_BMDC\_UP |  | 16 | 0.33 | 1.28 | 0.176 | 1.000 | 1.000 | 265 | tags=38%, list=19%, signal=46% |
| 202 | GSE5542\_IFNG\_VS\_IFNA\_TREATED\_EPITHELIAL\_CELLS\_24H\_UP |  | 18 | 0.31 | 1.28 | 0.176 | 1.000 | 1.000 | 274 | tags=39%, list=19%, signal=48% |
| 203 | GSE24634\_TREG\_VS\_TCONV\_POST\_DAY3\_IL4\_CONVERSION\_DN |  | 19 | 0.31 | 1.28 | 0.167 | 1.000 | 1.000 | 327 | tags=47%, list=23%, signal=61% |
| 204 | GSE27786\_NKTCELL\_VS\_NEUTROPHIL\_UP |  | 28 | 0.28 | 1.28 | 0.171 | 1.000 | 1.000 | 622 | tags=68%, list=44%, signal=119% |
| 205 | GSE27786\_CD4\_VS\_CD8\_TCELL\_DN |  | 28 | 0.27 | 1.28 | 0.176 | 1.000 | 1.000 | 837 | tags=86%, list=59%, signal=207% |
| 206 | GSE18281\_PERIMEDULLARY\_CORTICAL\_REGION\_VS\_WHOLE\_MEDULLA\_THYMUS\_UP |  | 15 | 0.34 | 1.28 | 0.172 | 1.000 | 1.000 | 212 | tags=33%, list=15%, signal=39% |
| 207 | GSE4142\_NAIVE\_VS\_MEMORY\_BCELL\_UP |  | 15 | 0.34 | 1.28 | 0.169 | 1.000 | 1.000 | 42 | tags=20%, list=3%, signal=20% |
| 208 | GSE29617\_CTRL\_VS\_DAY3\_TIV\_FLU\_VACCINE\_PBMC\_2008\_DN |  | 20 | 0.31 | 1.28 | 0.173 | 1.000 | 1.000 | 600 | tags=65%, list=43%, signal=111% |
| 209 | GSE21670\_UNTREATED\_VS\_TGFB\_TREATED\_STAT3\_KO\_CD4\_TCELL\_UP |  | 15 | 0.34 | 1.28 | 0.185 | 1.000 | 1.000 | 181 | tags=33%, list=13%, signal=38% |
| 210 | GSE16450\_IMMATURE\_VS\_MATURE\_NEURON\_CELL\_LINE\_DN |  | 24 | 0.28 | 1.28 | 0.193 | 1.000 | 1.000 | 697 | tags=71%, list=49%, signal=138% |
| 211 | GSE13411\_NAIVE\_VS\_SWITCHED\_MEMORY\_BCELL\_DN |  | 21 | 0.30 | 1.28 | 0.179 | 1.000 | 1.000 | 323 | tags=43%, list=23%, signal=55% |
| 212 | GSE25088\_CTRL\_VS\_IL4\_AND\_ROSIGLITAZONE\_STIM\_MACROPHAGE\_DN |  | 18 | 0.31 | 1.28 | 0.184 | 1.000 | 1.000 | 532 | tags=61%, list=38%, signal=97% |
| 213 | GSE17721\_CTRL\_VS\_POLYIC\_12H\_BMDC\_DN |  | 16 | 0.33 | 1.28 | 0.185 | 1.000 | 1.000 | 83 | tags=25%, list=6%, signal=26% |
| 214 | GSE17721\_POLYIC\_VS\_PAM3CSK4\_2H\_BMDC\_UP |  | 18 | 0.30 | 1.27 | 0.199 | 1.000 | 1.000 | 745 | tags=83%, list=53%, signal=174% |
| 215 | GSE6875\_TCONV\_VS\_FOXP3\_KO\_TREG\_DN |  | 16 | 0.32 | 1.27 | 0.198 | 1.000 | 1.000 | 747 | tags=81%, list=53%, signal=171% |
| 216 | GSE3982\_MAC\_VS\_NEUTROPHIL\_LPS\_STIM\_UP |  | 21 | 0.29 | 1.27 | 0.193 | 1.000 | 1.000 | 858 | tags=90%, list=61%, signal=227% |
| 217 | GSE411\_WT\_VS\_SOCS3\_KO\_MACROPHAGE\_IL6\_STIM\_100MIN\_DN |  | 26 | 0.27 | 1.27 | 0.175 | 1.000 | 1.000 | 279 | tags=35%, list=20%, signal=42% |
| 218 | GSE411\_UNSTIM\_VS\_400MIN\_IL6\_STIM\_MACROPHAGE\_UP |  | 26 | 0.28 | 1.27 | 0.174 | 1.000 | 1.000 | 771 | tags=81%, list=55%, signal=175% |
| 219 | GSE34156\_NOD2\_LIGAND\_VS\_TLR1\_TLR2\_LIGAND\_24H\_TREATED\_MONOCYTE\_DN |  | 18 | 0.31 | 1.27 | 0.201 | 1.000 | 1.000 | 733 | tags=83%, list=52%, signal=171% |
| 220 | GSE37301\_PRO\_BCELL\_VS\_RAG2\_KO\_NK\_CELL\_DN |  | 20 | 0.30 | 1.27 | 0.193 | 1.000 | 1.000 | 359 | tags=45%, list=25%, signal=60% |
| 221 | GSE40274\_CTRL\_VS\_FOXP3\_AND\_LEF1\_TRANSDUCED\_ACTIVATED\_CD4\_TCELL\_DN |  | 15 | 0.33 | 1.27 | 0.185 | 1.000 | 1.000 | 662 | tags=73%, list=47%, signal=137% |
| 222 | GSE32986\_UNSTIM\_VS\_GMCSF\_STIM\_DC\_UP |  | 21 | 0.29 | 1.27 | 0.199 | 1.000 | 1.000 | 495 | tags=57%, list=35%, signal=87% |
| 223 | GSE22886\_NAIVE\_CD4\_TCELL\_VS\_12H\_ACT\_TH1\_DN |  | 16 | 0.32 | 1.26 | 0.187 | 1.000 | 1.000 | 871 | tags=94%, list=62%, signal=242% |
| 224 | GSE34156\_NOD2\_LIGAND\_VS\_NOD2\_AND\_TLR1\_TLR2\_LIGAND\_6H\_TREATED\_MONOCYTE\_UP |  | 16 | 0.33 | 1.26 | 0.208 | 1.000 | 1.000 | 594 | tags=75%, list=42%, signal=128% |
| 225 | GSE9037\_WT\_VS\_IRAK4\_KO\_BMDM\_DN |  | 19 | 0.31 | 1.26 | 0.186 | 1.000 | 1.000 | 82 | tags=21%, list=6%, signal=22% |
| 226 | GSE7764\_IL15\_TREATED\_VS\_CTRL\_NK\_CELL\_24H\_UP |  | 27 | 0.27 | 1.26 | 0.194 | 1.000 | 1.000 | 911 | tags=93%, list=65%, signal=256% |
| 227 | GSE18281\_CORTICAL\_THYMOCYTE\_VS\_WHOLE\_CORTEX\_THYMUS\_DN |  | 16 | 0.32 | 1.26 | 0.203 | 1.000 | 1.000 | 313 | tags=44%, list=22%, signal=56% |
| 228 | GSE24671\_CTRL\_VS\_BAKIMULC\_INFECTED\_MOUSE\_SPLENOCYTES\_DN |  | 17 | 0.31 | 1.26 | 0.202 | 1.000 | 1.000 | 99 | tags=24%, list=7%, signal=25% |
| 229 | GSE12392\_WT\_VS\_IFNAR\_KO\_CD8A\_NEG\_SPLEEN\_DC\_DN |  | 28 | 0.27 | 1.26 | 0.187 | 1.000 | 1.000 | 728 | tags=79%, list=52%, signal=159% |
| 230 | GSE5463\_CTRL\_VS\_DEXAMETHASONE\_TREATED\_THYMOCYTE\_DN |  | 22 | 0.28 | 1.26 | 0.200 | 1.000 | 1.000 | 126 | tags=23%, list=9%, signal=25% |
| 231 | GSE28726\_ACT\_CD4\_TCELL\_VS\_ACT\_VA24NEG\_NKTCELL\_UP |  | 23 | 0.29 | 1.25 | 0.199 | 1.000 | 1.000 | 865 | tags=91%, list=61%, signal=232% |
| 232 | GSE360\_CTRL\_VS\_B\_MALAYI\_LOW\_DOSE\_MAC\_UP |  | 17 | 0.32 | 1.25 | 0.211 | 1.000 | 1.000 | 293 | tags=41%, list=21%, signal=51% |
| 233 | GSE9037\_WT\_VS\_IRAK4\_KO\_LPS\_1H\_STIM\_BMDM\_UP |  | 21 | 0.29 | 1.25 | 0.194 | 1.000 | 1.000 | 288 | tags=38%, list=20%, signal=47% |
| 234 | GSE5589\_UNSTIM\_VS\_45MIN\_LPS\_AND\_IL6\_STIM\_MACROPHAGE\_DN |  | 25 | 0.27 | 1.25 | 0.197 | 1.000 | 1.000 | 278 | tags=36%, list=20%, signal=44% |
| 235 | GSE28726\_NAIVE\_VS\_ACTIVATED\_VA24NEG\_NKTCELL\_UP |  | 30 | 0.26 | 1.25 | 0.191 | 1.000 | 1.000 | 921 | tags=93%, list=65%, signal=263% |
| 236 | GSE21670\_TGFB\_VS\_IL6\_TREATED\_STAT3\_KO\_CD4\_TCELL\_UP |  | 22 | 0.28 | 1.25 | 0.223 | 1.000 | 1.000 | 315 | tags=41%, list=22%, signal=52% |
| 237 | GSE29618\_PRE\_VS\_DAY7\_FLU\_VACCINE\_MONOCYTE\_DN |  | 20 | 0.29 | 1.25 | 0.197 | 1.000 | 1.000 | 265 | tags=35%, list=19%, signal=42% |
| 238 | GSE25085\_FETAL\_BM\_VS\_ADULT\_BM\_SP4\_THYMIC\_IMPLANT\_UP |  | 29 | 0.26 | 1.25 | 0.212 | 1.000 | 1.000 | 142 | tags=24%, list=10%, signal=26% |
| 239 | GSE2770\_IL12\_VS\_IL4\_TREATED\_ACT\_CD4\_TCELL\_2H\_UP |  | 15 | 0.33 | 1.25 | 0.216 | 1.000 | 1.000 | 668 | tags=73%, list=47%, signal=138% |
| 240 | GSE22432\_MULTIPOTENT\_VS\_COMMON\_DC\_PROGENITOR\_UNTREATED\_UP |  | 36 | 0.24 | 1.24 | 0.193 | 1.000 | 1.000 | 798 | tags=81%, list=57%, signal=181% |
| 241 | GSE2405\_HEAT\_KILLED\_LYSATE\_VS\_LIVE\_A\_PHAGOCYTOPHILUM\_STIM\_NEUTROPHIL\_24H\_DN |  | 21 | 0.29 | 1.24 | 0.193 | 1.000 | 1.000 | 397 | tags=48%, list=28%, signal=65% |
| 242 | GSE17186\_NAIVE\_VS\_CD21LOW\_TRANSITIONAL\_BCELL\_DN |  | 16 | 0.31 | 1.24 | 0.225 | 1.000 | 1.000 | 659 | tags=75%, list=47%, signal=139% |
| 243 | GSE14000\_UNSTIM\_VS\_4H\_LPS\_DC\_TRANSLATED\_RNA\_UP |  | 28 | 0.26 | 1.24 | 0.203 | 1.000 | 1.000 | 773 | tags=86%, list=55%, signal=186% |
| 244 | GSE44955\_MCSF\_VS\_MCSF\_AND\_IL27\_STIM\_MACROPHAGE\_UP |  | 24 | 0.28 | 1.24 | 0.191 | 1.000 | 1.000 | 69 | tags=21%, list=5%, signal=22% |
| 245 | GSE17974\_1.5H\_VS\_72H\_IL4\_AND\_ANTI\_IL12\_ACT\_CD4\_TCELL\_DN |  | 18 | 0.30 | 1.24 | 0.220 | 1.000 | 1.000 | 530 | tags=61%, list=38%, signal=97% |
| 246 | GSE6269\_FLU\_VS\_STAPH\_AUREUS\_INF\_PBMC\_UP |  | 17 | 0.32 | 1.24 | 0.219 | 1.000 | 1.000 | 631 | tags=71%, list=45%, signal=126% |
| 247 | GSE8685\_IL2\_STARVED\_VS\_IL15\_ACT\_IL2\_STARVED\_CD4\_TCELL\_UP |  | 24 | 0.27 | 1.23 | 0.224 | 1.000 | 1.000 | 461 | tags=50%, list=33%, signal=73% |
| 248 | GSE18893\_CTRL\_VS\_TNF\_TREATED\_TCONV\_2H\_DN |  | 28 | 0.26 | 1.23 | 0.212 | 1.000 | 1.000 | 643 | tags=68%, list=46%, signal=122% |
| 249 | GSE26928\_NAIVE\_VS\_CXCR5\_POS\_CD4\_TCELL\_DN |  | 22 | 0.28 | 1.23 | 0.213 | 1.000 | 1.000 | 61 | tags=18%, list=4%, signal=19% |
| 250 | GSE27786\_LSK\_VS\_MONO\_MAC\_UP |  | 26 | 0.27 | 1.23 | 0.200 | 1.000 | 1.000 | 646 | tags=65%, list=46%, signal=118% |
| 251 | GSE4142\_PLASMA\_CELL\_VS\_GC\_BCELL\_DN |  | 17 | 0.31 | 1.23 | 0.217 | 1.000 | 1.000 | 361 | tags=47%, list=26%, signal=62% |
| 252 | GSE11961\_MARGINAL\_ZONE\_BCELL\_VS\_PLASMA\_CELL\_DAY7\_DN |  | 19 | 0.29 | 1.23 | 0.243 | 1.000 | 1.000 | 353 | tags=42%, list=25%, signal=55% |
| 253 | GSE21927\_SPLEEN\_VS\_C26GM\_TUMOR\_MONOCYTE\_BALBC\_DN |  | 17 | 0.31 | 1.22 | 0.232 | 1.000 | 1.000 | 390 | tags=53%, list=28%, signal=72% |
| 254 | GSE17721\_0.5H\_VS\_24H\_POLYIC\_BMDC\_UP |  | 19 | 0.30 | 1.22 | 0.246 | 1.000 | 1.000 | 925 | tags=95%, list=66%, signal=271% |
| 255 | GSE17721\_0.5H\_VS\_4H\_GARDIQUIMOD\_BMDC\_DN |  | 17 | 0.30 | 1.22 | 0.233 | 1.000 | 1.000 | 165 | tags=29%, list=12%, signal=33% |
| 256 | GSE45365\_HEALTHY\_VS\_MCMV\_INFECTION\_CD8A\_DC\_IFNAR\_KO\_DN |  | 25 | 0.26 | 1.22 | 0.235 | 1.000 | 1.000 | 240 | tags=32%, list=17%, signal=38% |
| 257 | GSE19888\_NO\_PRETREAT\_VS\_ADENOSINE\_A3R\_INHIBITOR\_PRETREATED\_MAST\_CELL\_TCELL\_MEMBRANES\_ACT\_UP |  | 20 | 0.29 | 1.22 | 0.225 | 1.000 | 1.000 | 780 | tags=85%, list=55%, signal=187% |
| 258 | GSE3982\_EOSINOPHIL\_VS\_DC\_UP |  | 27 | 0.26 | 1.22 | 0.203 | 1.000 | 1.000 | 62 | tags=19%, list=4%, signal=19% |
| 259 | GSE21033\_CTRL\_VS\_POLYIC\_STIM\_DC\_3H\_UP |  | 18 | 0.30 | 1.22 | 0.235 | 1.000 | 1.000 | 570 | tags=67%, list=40%, signal=110% |
| 260 | GSE360\_CTRL\_VS\_L\_DONOVANI\_MAC\_UP |  | 21 | 0.28 | 1.22 | 0.240 | 1.000 | 1.000 | 742 | tags=76%, list=53%, signal=158% |
| 261 | GSE17974\_CTRL\_VS\_ACT\_IL4\_AND\_ANTI\_IL12\_1H\_CD4\_TCELL\_DN |  | 20 | 0.29 | 1.22 | 0.229 | 1.000 | 1.000 | 201 | tags=30%, list=14%, signal=34% |
| 262 | GSE20715\_WT\_VS\_TLR4\_KO\_LUNG\_UP |  | 15 | 0.31 | 1.21 | 0.241 | 1.000 | 1.000 | 5 | tags=13%, list=0%, signal=13% |
| 263 | GSE22611\_UNSTIM\_VS\_6H\_MDP\_STIM\_NOD2\_TRANSDUCED\_HEK293T\_CELL\_UP |  | 17 | 0.30 | 1.21 | 0.229 | 1.000 | 1.000 | 83 | tags=24%, list=6%, signal=25% |
| 264 | GSE25088\_CTRL\_VS\_ROSIGLITAZONE\_STIM\_STAT6\_KO\_MACROPHAGE\_DN |  | 23 | 0.28 | 1.21 | 0.250 | 1.000 | 1.000 | 601 | tags=65%, list=43%, signal=112% |
| 265 | GSE16386\_IL4\_VS\_IL4\_AND\_ROSIGLITAZONE\_STIM\_MACROPHAGE\_6H\_UP |  | 18 | 0.30 | 1.21 | 0.249 | 1.000 | 1.000 | 681 | tags=78%, list=48%, signal=148% |
| 266 | GSE360\_L\_MAJOR\_VS\_T\_GONDII\_DC\_DN |  | 15 | 0.32 | 1.21 | 0.239 | 1.000 | 1.000 | 596 | tags=73%, list=42%, signal=126% |
| 267 | GSE17721\_POLYIC\_VS\_CPG\_12H\_BMDC\_DN |  | 18 | 0.30 | 1.20 | 0.241 | 1.000 | 1.000 | 319 | tags=39%, list=23%, signal=50% |
| 268 | GSE16385\_UNTREATED\_VS\_12H\_IFNG\_TNF\_TREATED\_MACROPHAGE\_DN |  | 17 | 0.30 | 1.20 | 0.245 | 1.000 | 1.000 | 250 | tags=35%, list=18%, signal=42% |
| 269 | GSE27786\_BCELL\_VS\_MONO\_MAC\_UP |  | 25 | 0.26 | 1.20 | 0.227 | 1.000 | 1.000 | 837 | tags=84%, list=59%, signal=203% |
| 270 | GSE37605\_C57BL6\_VS\_NOD\_FOXP3\_FUSION\_GFP\_TCONV\_DN |  | 23 | 0.27 | 1.20 | 0.245 | 1.000 | 1.000 | 213 | tags=30%, list=15%, signal=35% |
| 271 | GSE26290\_WT\_VS\_PDK1\_KO\_ANTI\_CD3\_AND\_IL2\_STIM\_CD8\_TCELL\_UP |  | 19 | 0.29 | 1.20 | 0.248 | 1.000 | 1.000 | 70 | tags=21%, list=5%, signal=22% |
| 272 | GSE21927\_HEALTHY\_VS\_TUMOROUS\_BALBC\_MOUSE\_MONOCYTE\_UP |  | 23 | 0.27 | 1.19 | 0.257 | 1.000 | 1.000 | 330 | tags=43%, list=23%, signal=56% |
| 273 | GSE11864\_CSF1\_IFNG\_VS\_CSF1\_PAM3CYS\_IN\_MAC\_UP |  | 25 | 0.26 | 1.19 | 0.247 | 1.000 | 1.000 | 421 | tags=44%, list=30%, signal=62% |
| 274 | GSE5542\_IFNG\_VS\_IFNA\_AND\_IFNG\_TREATED\_EPITHELIAL\_CELLS\_24H\_UP |  | 24 | 0.26 | 1.19 | 0.247 | 1.000 | 1.000 | 129 | tags=21%, list=9%, signal=23% |
| 275 | GSE5589\_UNSTIM\_VS\_180MIN\_LPS\_STIM\_MACROPHAGE\_UP |  | 25 | 0.26 | 1.19 | 0.244 | 1.000 | 1.000 | 278 | tags=36%, list=20%, signal=44% |
| 276 | GSE21927\_EL4\_VS\_MCA203\_TUMOR\_MONOCYTES\_DN |  | 17 | 0.30 | 1.19 | 0.231 | 1.000 | 1.000 | 418 | tags=53%, list=30%, signal=74% |
| 277 | GSE39820\_TGFBETA1\_VS\_TGFBETA3\_IN\_IL6\_IL23A\_TREATED\_CD4\_TCELL\_UP |  | 21 | 0.27 | 1.19 | 0.259 | 1.000 | 1.000 | 663 | tags=76%, list=47%, signal=142% |
| 278 | GSE24671\_BAKIMULC\_VS\_SENDAI\_VIRUS\_INFECTED\_MOUSE\_SPLENOCYTES\_DN |  | 20 | 0.28 | 1.19 | 0.247 | 1.000 | 1.000 | 686 | tags=75%, list=49%, signal=144% |
| 279 | GSE34156\_UNTREATED\_VS\_6H\_TLR1\_TLR2\_LIGAND\_TREATED\_MONOCYTE\_UP |  | 19 | 0.28 | 1.19 | 0.258 | 1.000 | 1.000 | 254 | tags=37%, list=18%, signal=44% |
| 280 | GSE36078\_UNTREATED\_VS\_AD5\_INF\_IL1R\_KO\_MOUSE\_LUNG\_DC\_DN |  | 20 | 0.28 | 1.18 | 0.267 | 1.000 | 1.000 | 224 | tags=30%, list=16%, signal=35% |
| 281 | GSE27786\_NKCELL\_VS\_MONO\_MAC\_UP |  | 15 | 0.31 | 1.18 | 0.262 | 1.000 | 1.000 | 801 | tags=87%, list=57%, signal=198% |
| 282 | GSE27786\_NKTCELL\_VS\_NEUTROPHIL\_DN |  | 22 | 0.27 | 1.18 | 0.264 | 1.000 | 1.000 | 170 | tags=27%, list=12%, signal=31% |
| 283 | GSE16385\_MONOCYTE\_VS\_12H\_ROSIGLITAZONE\_TREATED\_MACROPHAGE\_DN |  | 19 | 0.28 | 1.18 | 0.269 | 1.000 | 1.000 | 360 | tags=42%, list=26%, signal=56% |
| 284 | GSE3982\_MAC\_VS\_EFF\_MEMORY\_CD4\_TCELL\_UP |  | 26 | 0.25 | 1.18 | 0.261 | 1.000 | 1.000 | 289 | tags=31%, list=20%, signal=38% |
| 285 | GSE369\_IFNG\_KO\_VS\_WT\_LIVER\_UP |  | 27 | 0.25 | 1.18 | 0.256 | 1.000 | 1.000 | 26 | tags=15%, list=2%, signal=15% |
| 286 | GSE3982\_MAC\_VS\_NEUTROPHIL\_UP |  | 16 | 0.31 | 1.18 | 0.263 | 1.000 | 1.000 | 490 | tags=56%, list=35%, signal=85% |
| 287 | GSE4984\_LPS\_VS\_VEHICLE\_CTRL\_TREATED\_DC\_UP |  | 21 | 0.26 | 1.18 | 0.252 | 1.000 | 1.000 | 251 | tags=33%, list=18%, signal=40% |
| 288 | GSE13411\_NAIVE\_VS\_IGM\_MEMORY\_BCELL\_DN |  | 16 | 0.30 | 1.18 | 0.262 | 1.000 | 1.000 | 325 | tags=44%, list=23%, signal=56% |
| 289 | GSE17721\_POLYIC\_VS\_PAM3CSK4\_16H\_BMDC\_DN |  | 21 | 0.27 | 1.17 | 0.255 | 1.000 | 1.000 | 728 | tags=76%, list=52%, signal=155% |
| 290 | GSE9509\_LPS\_VS\_LPS\_AND\_IL10\_STIM\_IL10\_KO\_MACROPHAGE\_10MIN\_DN |  | 15 | 0.31 | 1.17 | 0.256 | 1.000 | 1.000 | 522 | tags=67%, list=37%, signal=105% |
| 291 | GSE2405\_S\_AUREUS\_VS\_UNTREATED\_NEUTROPHIL\_UP |  | 16 | 0.30 | 1.17 | 0.287 | 1.000 | 1.000 | 902 | tags=94%, list=64%, signal=257% |
| 292 | GSE14350\_IL2RB\_KO\_VS\_WT\_TEFF\_UP |  | 15 | 0.32 | 1.17 | 0.263 | 1.000 | 1.000 | 34 | tags=20%, list=2%, signal=20% |
| 293 | GSE17721\_LPS\_VS\_PAM3CSK4\_4H\_BMDC\_DN |  | 21 | 0.27 | 1.17 | 0.276 | 1.000 | 1.000 | 265 | tags=33%, list=19%, signal=40% |
| 294 | GSE29949\_CD8\_POS\_DC\_SPLEEN\_VS\_DC\_BRAIN\_UP |  | 24 | 0.26 | 1.17 | 0.269 | 1.000 | 1.000 | 127 | tags=21%, list=9%, signal=23% |
| 295 | GSE6259\_FLT3L\_INDUCED\_33D1\_POS\_DC\_VS\_BCELL\_DN |  | 16 | 0.30 | 1.17 | 0.262 | 1.000 | 1.000 | 536 | tags=56%, list=38%, signal=90% |
| 296 | GSE18148\_CBFB\_KO\_VS\_WT\_TREG\_UP |  | 16 | 0.30 | 1.17 | 0.279 | 1.000 | 1.000 | 522 | tags=63%, list=37%, signal=98% |
| 297 | GSE1460\_CD4\_THYMOCYTE\_VS\_NAIVE\_CD4\_TCELL\_CORD\_BLOOD\_UP |  | 17 | 0.29 | 1.17 | 0.276 | 1.000 | 1.000 | 508 | tags=53%, list=36%, signal=82% |
| 298 | GSE46606\_IRF4HIGH\_VS\_WT\_CD40L\_IL2\_IL5\_DAY1\_STIMULATED\_BCELL\_UP |  | 17 | 0.30 | 1.17 | 0.284 | 1.000 | 1.000 | 603 | tags=65%, list=43%, signal=112% |
| 299 | GSE17721\_0.5H\_VS\_24H\_LPS\_BMDC\_DN |  | 18 | 0.29 | 1.16 | 0.274 | 1.000 | 1.000 | 74 | tags=22%, list=5%, signal=23% |
| 300 | GSE2197\_CPG\_DNA\_VS\_UNTREATED\_IN\_DC\_UP |  | 22 | 0.27 | 1.16 | 0.275 | 1.000 | 1.000 | 165 | tags=27%, list=12%, signal=30% |
| 301 | GSE20484\_MCSG\_VS\_CXCL4\_MONOCYTE\_DERIVED\_MACROPHAGE\_UP |  | 23 | 0.26 | 1.16 | 0.272 | 1.000 | 1.000 | 243 | tags=30%, list=17%, signal=36% |
| 302 | GSE20727\_CTRL\_VS\_ROS\_INH\_AND\_DNFB\_ALLERGEN\_TREATED\_DC\_DN |  | 18 | 0.29 | 1.16 | 0.285 | 1.000 | 1.000 | 353 | tags=39%, list=25%, signal=51% |
| 303 | GSE19888\_ADENOSINE\_A3R\_ACT\_VS\_A3R\_ACT\_WITH\_A3R\_INH\_PRETREATMENT\_IN\_MAST\_CELL\_UP |  | 24 | 0.25 | 1.16 | 0.288 | 1.000 | 1.000 | 921 | tags=92%, list=65%, signal=259% |
| 304 | GSE39820\_TGFBETA1\_VS\_TGFBETA3\_IN\_IL6\_TREATED\_CD4\_TCELL\_UP |  | 23 | 0.26 | 1.16 | 0.280 | 1.000 | 1.000 | 227 | tags=30%, list=16%, signal=36% |
| 305 | GSE23695\_CD57\_POS\_VS\_NEG\_NK\_CELL\_UP |  | 25 | 0.25 | 1.16 | 0.275 | 1.000 | 1.000 | 736 | tags=68%, list=52%, signal=140% |
| 306 | GSE17721\_0.5H\_VS\_4H\_CPG\_BMDC\_DN |  | 20 | 0.28 | 1.16 | 0.277 | 1.000 | 1.000 | 93 | tags=20%, list=7%, signal=21% |
| 307 | GSE45365\_HEALTHY\_VS\_MCMV\_INFECTION\_BCELL\_IFNAR\_KO\_DN |  | 23 | 0.26 | 1.15 | 0.287 | 1.000 | 1.000 | 68 | tags=17%, list=5%, signal=18% |
| 308 | GSE13485\_DAY3\_VS\_DAY21\_YF17D\_VACCINE\_PBMC\_UP |  | 28 | 0.24 | 1.15 | 0.279 | 1.000 | 1.000 | 130 | tags=21%, list=9%, signal=23% |
| 309 | GSE14000\_TRANSLATED\_RNA\_VS\_MRNA\_4H\_LPS\_DC\_UP |  | 20 | 0.27 | 1.15 | 0.303 | 1.000 | 1.000 | 807 | tags=85%, list=57%, signal=196% |
| 310 | GSE21927\_HEALTHY\_VS\_TUMOROUS\_BALBC\_MOUSE\_MONOCYTE\_DN |  | 16 | 0.29 | 1.15 | 0.298 | 1.000 | 1.000 | 360 | tags=44%, list=26%, signal=58% |
| 311 | GSE20152\_SPHK1\_KO\_VS\_HTNFA\_OVEREXPRESS\_ANKLE\_DN |  | 28 | 0.24 | 1.15 | 0.284 | 1.000 | 1.000 | 754 | tags=75%, list=53%, signal=158% |
| 312 | GSE14000\_UNSTIM\_VS\_16H\_LPS\_DC\_UP |  | 25 | 0.25 | 1.15 | 0.295 | 1.000 | 1.000 | 603 | tags=60%, list=43%, signal=103% |
| 313 | GSE7852\_LN\_VS\_FAT\_TCONV\_UP |  | 22 | 0.26 | 1.15 | 0.294 | 1.000 | 1.000 | 367 | tags=41%, list=26%, signal=54% |
| 314 | GSE14769\_40MIN\_VS\_360MIN\_LPS\_BMDM\_UP |  | 20 | 0.27 | 1.15 | 0.307 | 1.000 | 1.000 | 190 | tags=25%, list=13%, signal=28% |
| 315 | GSE17721\_POLYIC\_VS\_GARDIQUIMOD\_12H\_BMDC\_UP |  | 20 | 0.27 | 1.15 | 0.280 | 1.000 | 1.000 | 263 | tags=30%, list=19%, signal=36% |
| 316 | GSE29949\_CD8\_NEG\_DC\_SPLEEN\_VS\_CD8\_POS\_DC\_SPLEEN\_DN |  | 19 | 0.28 | 1.15 | 0.283 | 1.000 | 1.000 | 322 | tags=37%, list=23%, signal=47% |
| 317 | GSE411\_UNSTIM\_VS\_400MIN\_IL6\_STIM\_MACROPHAGE\_DN |  | 16 | 0.30 | 1.15 | 0.310 | 1.000 | 1.000 | 782 | tags=81%, list=55%, signal=180% |
| 318 | GSE37301\_GRANULOCYTE\_MONOCYTE\_PROGENITOR\_VS\_RAG2\_KO\_NK\_CELL\_UP |  | 21 | 0.27 | 1.15 | 0.291 | 1.000 | 1.000 | 511 | tags=48%, list=36%, signal=74% |
| 319 | GSE17721\_CTRL\_VS\_CPG\_4H\_BMDC\_DN |  | 15 | 0.31 | 1.15 | 0.315 | 1.000 | 1.000 | 51 | tags=20%, list=4%, signal=21% |
| 320 | GSE17721\_CTRL\_VS\_GARDIQUIMOD\_24H\_BMDC\_UP |  | 16 | 0.30 | 1.15 | 0.303 | 1.000 | 1.000 | 860 | tags=94%, list=61%, signal=237% |
| 321 | GSE11961\_MARGINAL\_ZONE\_BCELL\_VS\_PLASMA\_CELL\_DAY7\_UP |  | 16 | 0.29 | 1.15 | 0.280 | 1.000 | 1.000 | 669 | tags=75%, list=47%, signal=141% |
| 322 | GSE3982\_CTRL\_VS\_LPS\_4H\_MAC\_UP |  | 25 | 0.25 | 1.15 | 0.283 | 1.000 | 1.000 | 573 | tags=64%, list=41%, signal=106% |
| 323 | GSE3982\_EOSINOPHIL\_VS\_TH2\_UP |  | 29 | 0.23 | 1.14 | 0.288 | 1.000 | 1.000 | 424 | tags=45%, list=30%, signal=63% |
| 324 | GSE27786\_LIN\_NEG\_VS\_NEUTROPHIL\_UP |  | 24 | 0.25 | 1.14 | 0.280 | 1.000 | 1.000 | 797 | tags=79%, list=56%, signal=179% |
| 325 | GSE21546\_WT\_VS\_SAP1A\_KO\_ANTI\_CD3\_STIM\_DP\_THYMOCYTES\_UP |  | 19 | 0.28 | 1.14 | 0.283 | 1.000 | 1.000 | 278 | tags=37%, list=20%, signal=45% |
| 326 | GSE24210\_RESTING\_TREG\_VS\_TCONV\_DN |  | 26 | 0.25 | 1.14 | 0.276 | 1.000 | 1.000 | 847 | tags=85%, list=60%, signal=208% |
| 327 | GSE17721\_POLYIC\_VS\_CPG\_24H\_BMDC\_DN |  | 24 | 0.25 | 1.14 | 0.288 | 1.000 | 1.000 | 92 | tags=21%, list=7%, signal=22% |
| 328 | GSE14308\_TH17\_VS\_INDUCED\_TREG\_DN |  | 16 | 0.29 | 1.14 | 0.299 | 1.000 | 1.000 | 539 | tags=69%, list=38%, signal=110% |
| 329 | GSE2770\_TGFB\_AND\_IL4\_VS\_IL4\_TREATED\_ACT\_CD4\_TCELL\_48H\_UP |  | 26 | 0.25 | 1.14 | 0.320 | 1.000 | 1.000 | 173 | tags=23%, list=12%, signal=26% |
| 330 | GSE32423\_CTRL\_VS\_IL7\_IL4\_MEMORY\_CD8\_TCELL\_DN |  | 21 | 0.26 | 1.14 | 0.328 | 1.000 | 1.000 | 547 | tags=52%, list=39%, signal=84% |
| 331 | GSE14308\_INDUCED\_VS\_NATURAL\_TREG\_UP |  | 20 | 0.27 | 1.14 | 0.277 | 1.000 | 1.000 | 723 | tags=75%, list=51%, signal=152% |
| 332 | GSE17721\_CTRL\_VS\_GARDIQUIMOD\_4H\_BMDC\_DN |  | 17 | 0.29 | 1.14 | 0.283 | 1.000 | 1.000 | 180 | tags=29%, list=13%, signal=33% |
| 333 | GSE24292\_WT\_VS\_PPARG\_KO\_MACROPHAGE\_UP |  | 18 | 0.27 | 1.14 | 0.296 | 1.000 | 1.000 | 335 | tags=39%, list=24%, signal=50% |
| 334 | GSE9988\_ANTI\_TREM1\_VS\_ANTI\_TREM1\_AND\_LPS\_MONOCYTE\_UP |  | 20 | 0.27 | 1.14 | 0.310 | 1.000 | 1.000 | 249 | tags=30%, list=18%, signal=36% |
| 335 | GSE2770\_TGFB\_AND\_IL4\_ACT\_VS\_ACT\_CD4\_TCELL\_6H\_DN |  | 21 | 0.26 | 1.14 | 0.306 | 1.000 | 1.000 | 624 | tags=67%, list=44%, signal=118% |
| 336 | GSE15324\_NAIVE\_VS\_ACTIVATED\_CD8\_TCELL\_DN |  | 17 | 0.28 | 1.14 | 0.290 | 1.000 | 1.000 | 339 | tags=41%, list=24%, signal=54% |
| 337 | GSE13522\_WT\_VS\_IFNAR\_KO\_SKIN\_UP |  | 17 | 0.28 | 1.14 | 0.304 | 1.000 | 1.000 | 261 | tags=35%, list=18%, signal=43% |
| 338 | GSE22589\_SIV\_VS\_HIV\_AND\_SIV\_INFECTED\_DC\_DN |  | 21 | 0.27 | 1.13 | 0.320 | 1.000 | 1.000 | 335 | tags=43%, list=24%, signal=55% |
| 339 | GSE3982\_MEMORY\_CD4\_TCELL\_VS\_TH2\_UP |  | 29 | 0.23 | 1.13 | 0.293 | 1.000 | 1.000 | 92 | tags=17%, list=7%, signal=18% |
| 340 | GSE18791\_CTRL\_VS\_NEWCASTLE\_VIRUS\_DC\_16H\_UP |  | 35 | 0.21 | 1.13 | 0.296 | 1.000 | 1.000 | 847 | tags=86%, list=60%, signal=209% |
| 341 | GSE339\_CD4POS\_VS\_CD4CD8DN\_DC\_IN\_CULTURE\_UP |  | 17 | 0.28 | 1.13 | 0.326 | 1.000 | 1.000 | 688 | tags=76%, list=49%, signal=147% |
| 342 | GSE7460\_CTRL\_VS\_TGFB\_TREATED\_ACT\_FOXP3\_HET\_TCONV\_UP |  | 24 | 0.26 | 1.13 | 0.310 | 1.000 | 1.000 | 213 | tags=29%, list=15%, signal=34% |
| 343 | GSE45365\_CD8A\_DC\_VS\_CD11B\_DC\_IFNAR\_KO\_UP |  | 16 | 0.29 | 1.13 | 0.294 | 1.000 | 1.000 | 156 | tags=25%, list=11%, signal=28% |
| 344 | GSE20198\_IL12\_IL18\_VS\_IFNA\_TREATED\_ACT\_CD4\_TCELL\_UP |  | 22 | 0.26 | 1.13 | 0.324 | 1.000 | 1.000 | 624 | tags=68%, list=44%, signal=120% |
| 345 | GSE37301\_HEMATOPOIETIC\_STEM\_CELL\_VS\_COMMON\_LYMPHOID\_PROGENITOR\_DN |  | 22 | 0.26 | 1.13 | 0.316 | 1.000 | 1.000 | 451 | tags=50%, list=32%, signal=72% |
| 346 | GSE29618\_PRE\_VS\_DAY7\_POST\_TIV\_FLU\_VACCINE\_BCELL\_DN |  | 22 | 0.26 | 1.13 | 0.288 | 1.000 | 1.000 | 187 | tags=27%, list=13%, signal=31% |
| 347 | GSE13306\_LAMINA\_PROPRIA\_VS\_SPLEEN\_TREG\_UP |  | 27 | 0.24 | 1.13 | 0.317 | 1.000 | 1.000 | 37 | tags=15%, list=3%, signal=15% |
| 348 | GSE17721\_CTRL\_VS\_POLYIC\_6H\_BMDC\_DN |  | 18 | 0.27 | 1.12 | 0.319 | 1.000 | 1.000 | 198 | tags=28%, list=14%, signal=32% |
| 349 | GSE9509\_10MIN\_VS\_30MIN\_LPS\_AND\_IL10\_STIM\_IL10\_KO\_MACROPHAGE\_UP |  | 17 | 0.28 | 1.12 | 0.300 | 1.000 | 1.000 | 831 | tags=88%, list=59%, signal=212% |
| 350 | GSE14769\_UNSTIM\_VS\_240MIN\_LPS\_BMDM\_DN |  | 29 | 0.24 | 1.12 | 0.307 | 1.000 | 1.000 | 132 | tags=21%, list=9%, signal=22% |
| 351 | GSE20198\_IL12\_IL18\_VS\_IFNA\_TREATED\_ACT\_CD4\_TCELL\_DN |  | 19 | 0.27 | 1.12 | 0.336 | 1.000 | 1.000 | 49 | tags=16%, list=3%, signal=16% |
| 352 | GSE45365\_CD8A\_DC\_VS\_CD11B\_DC\_IFNAR\_KO\_MCMV\_INFECTION\_UP |  | 18 | 0.28 | 1.12 | 0.325 | 1.000 | 1.000 | 516 | tags=56%, list=37%, signal=86% |
| 353 | GSE12366\_PLASMA\_CELL\_VS\_NAIVE\_BCELL\_DN |  | 22 | 0.25 | 1.12 | 0.312 | 1.000 | 1.000 | 448 | tags=45%, list=32%, signal=66% |
| 354 | GSE32901\_NAIVE\_VS\_TH17\_NEG\_CD4\_TCELL\_UP |  | 17 | 0.28 | 1.12 | 0.301 | 1.000 | 1.000 | 132 | tags=24%, list=9%, signal=26% |
| 355 | GSE10094\_LCMV\_VS\_LISTERIA\_IND\_EFF\_CD4\_TCELL\_UP |  | 19 | 0.27 | 1.12 | 0.314 | 1.000 | 1.000 | 43 | tags=16%, list=3%, signal=16% |
| 356 | GSE360\_L\_MAJOR\_VS\_M\_TUBERCULOSIS\_DC\_DN |  | 16 | 0.29 | 1.12 | 0.309 | 1.000 | 1.000 | 55 | tags=19%, list=4%, signal=19% |
| 357 | GSE3565\_CTRL\_VS\_LPS\_INJECTED\_SPLENOCYTES\_DN |  | 19 | 0.27 | 1.12 | 0.315 | 1.000 | 1.000 | 261 | tags=32%, list=18%, signal=38% |
| 358 | GSE22935\_UNSTIM\_VS\_12H\_MBOVIS\_BCG\_STIM\_MYD88\_KO\_MACROPHAGE\_UP |  | 16 | 0.29 | 1.12 | 0.322 | 1.000 | 1.000 | 95 | tags=25%, list=7%, signal=27% |
| 359 | GSE34156\_NOD2\_LIGAND\_VS\_NOD2\_AND\_TLR1\_TLR2\_LIGAND\_24H\_TREATED\_MONOCYTE\_DN |  | 15 | 0.30 | 1.12 | 0.323 | 1.000 | 1.000 | 449 | tags=53%, list=32%, signal=77% |
| 360 | GSE17974\_CTRL\_VS\_ACT\_IL4\_AND\_ANTI\_IL12\_4H\_CD4\_TCELL\_DN |  | 39 | 0.21 | 1.12 | 0.306 | 1.000 | 1.000 | 261 | tags=28%, list=18%, signal=34% |
| 361 | GSE20366\_TREG\_VS\_NAIVE\_CD4\_TCELL\_HOMEOSTATIC\_CONVERSION\_DN |  | 19 | 0.27 | 1.12 | 0.331 | 1.000 | 1.000 | 305 | tags=37%, list=22%, signal=46% |
| 362 | GSE17721\_CTRL\_VS\_PAM3CSK4\_0.5H\_BMDC\_UP |  | 19 | 0.28 | 1.12 | 0.334 | 1.000 | 1.000 | 432 | tags=47%, list=31%, signal=67% |
| 363 | GSE31082\_DN\_VS\_CD4\_SP\_THYMOCYTE\_DN |  | 16 | 0.28 | 1.12 | 0.303 | 1.000 | 1.000 | 387 | tags=44%, list=27%, signal=60% |
| 364 | GSE43955\_TGFB\_IL6\_VS\_TGFB\_IL6\_IL23\_TH17\_ACT\_CD4\_TCELL\_52H\_DN |  | 29 | 0.23 | 1.11 | 0.323 | 1.000 | 1.000 | 35 | tags=14%, list=2%, signal=14% |
| 365 | GSE13493\_DP\_VS\_CD8POS\_THYMOCYTE\_DN |  | 24 | 0.25 | 1.11 | 0.345 | 1.000 | 1.000 | 69 | tags=17%, list=5%, signal=17% |
| 366 | GSE37416\_12H\_VS\_24H\_F\_TULARENSIS\_LVS\_NEUTROPHIL\_UP |  | 18 | 0.28 | 1.11 | 0.330 | 1.000 | 1.000 | 191 | tags=28%, list=14%, signal=32% |
| 367 | GSE2706\_R848\_VS\_LPS\_2H\_STIM\_DC\_UP |  | 16 | 0.29 | 1.11 | 0.335 | 1.000 | 1.000 | 422 | tags=50%, list=30%, signal=71% |
| 368 | GSE18804\_SPLEEN\_MACROPHAGE\_VS\_BRAIN\_TUMORAL\_MACROPHAGE\_DN |  | 24 | 0.24 | 1.11 | 0.345 | 1.000 | 1.000 | 151 | tags=21%, list=11%, signal=23% |
| 369 | GSE22033\_UNTREATED\_VS\_MRL24\_TREATED\_MEF\_DN |  | 21 | 0.26 | 1.11 | 0.321 | 1.000 | 1.000 | 131 | tags=19%, list=9%, signal=21% |
| 370 | GSE1566\_WT\_VS\_EZH2\_KO\_LN\_TCELL\_DN |  | 18 | 0.27 | 1.11 | 0.329 | 1.000 | 1.000 | 100 | tags=22%, list=7%, signal=24% |
| 371 | GSE13485\_CTRL\_VS\_DAY7\_YF17D\_VACCINE\_PBMC\_UP |  | 16 | 0.28 | 1.11 | 0.327 | 1.000 | 1.000 | 524 | tags=56%, list=37%, signal=88% |
| 372 | GSE16266\_CTRL\_VS\_HEATSHOCK\_AND\_LPS\_STIM\_MEF\_DN |  | 24 | 0.24 | 1.11 | 0.331 | 1.000 | 1.000 | 997 | tags=96%, list=71%, signal=321% |
| 373 | GSE22935\_WT\_VS\_MYD88\_KO\_MACROPHAGE\_12H\_MBOVIS\_BCG\_STIM\_DN |  | 16 | 0.29 | 1.11 | 0.332 | 1.000 | 1.000 | 353 | tags=44%, list=25%, signal=58% |
| 374 | GSE14908\_ATOPIC\_VS\_NONATOPIC\_PATIENT\_HDM\_STIM\_CD4\_TCELL\_DN |  | 28 | 0.23 | 1.10 | 0.334 | 1.000 | 1.000 | 873 | tags=86%, list=62%, signal=220% |
| 375 | GSE31082\_DN\_VS\_CD8\_SP\_THYMOCYTE\_UP |  | 23 | 0.25 | 1.10 | 0.344 | 1.000 | 1.000 | 570 | tags=65%, list=40%, signal=108% |
| 376 | GSE15330\_LYMPHOID\_MULTIPOTENT\_VS\_MEGAKARYOCYTE\_ERYTHROID\_PROGENITOR\_UP |  | 26 | 0.24 | 1.10 | 0.331 | 1.000 | 1.000 | 545 | tags=58%, list=39%, signal=92% |
| 377 | GSE3982\_EOSINOPHIL\_VS\_CENT\_MEMORY\_CD4\_TCELL\_DN |  | 16 | 0.28 | 1.10 | 0.333 | 1.000 | 1.000 | 570 | tags=63%, list=40%, signal=104% |
| 378 | GSE16385\_UNTREATED\_VS\_12H\_IL4\_TREATED\_MACROPHAGE\_UP |  | 15 | 0.29 | 1.10 | 0.326 | 1.000 | 1.000 | 186 | tags=33%, list=13%, signal=38% |
| 379 | GSE2706\_UNSTIM\_VS\_2H\_R848\_DC\_UP |  | 24 | 0.24 | 1.10 | 0.342 | 1.000 | 1.000 | 288 | tags=33%, list=20%, signal=41% |
| 380 | GSE18281\_SUBCAPSULAR\_VS\_CENTRAL\_CORTICAL\_REGION\_OF\_THYMUS\_UP |  | 25 | 0.24 | 1.10 | 0.352 | 1.000 | 1.000 | 250 | tags=28%, list=18%, signal=33% |
| 381 | GSE25085\_FETAL\_BM\_VS\_ADULT\_BM\_SP4\_THYMIC\_IMPLANT\_DN |  | 27 | 0.23 | 1.10 | 0.346 | 1.000 | 1.000 | 57 | tags=15%, list=4%, signal=15% |
| 382 | GSE21927\_SPLEEN\_VS\_BONE\_MARROW\_MONOCYTE\_BALBC\_DN |  | 16 | 0.28 | 1.10 | 0.327 | 1.000 | 1.000 | 206 | tags=31%, list=15%, signal=36% |
| 383 | GSE2770\_UNTREATED\_VS\_ACT\_CD4\_TCELL\_2H\_DN |  | 21 | 0.25 | 1.09 | 0.352 | 1.000 | 1.000 | 362 | tags=38%, list=26%, signal=50% |
| 384 | GSE27786\_LIN\_NEG\_VS\_MONO\_MAC\_DN |  | 22 | 0.25 | 1.09 | 0.363 | 1.000 | 1.000 | 131 | tags=23%, list=9%, signal=25% |
| 385 | GSE45365\_NK\_CELL\_VS\_CD8\_TCELL\_UP |  | 23 | 0.25 | 1.09 | 0.358 | 1.000 | 1.000 | 281 | tags=35%, list=20%, signal=43% |
| 386 | GSE22432\_PDC\_VS\_TGFB1\_TREATEDCOMMON\_DC\_PROGENITOR\_UP |  | 17 | 0.27 | 1.09 | 0.340 | 1.000 | 1.000 | 549 | tags=59%, list=39%, signal=95% |
| 387 | GSE13229\_IMM\_VS\_MATURE\_NKCELL\_DN |  | 20 | 0.26 | 1.09 | 0.359 | 1.000 | 1.000 | 414 | tags=45%, list=29%, signal=63% |
| 388 | GSE2770\_UNTREATED\_VS\_IL4\_TREATED\_ACT\_CD4\_TCELL\_2H\_DN |  | 19 | 0.26 | 1.09 | 0.349 | 1.000 | 1.000 | 818 | tags=84%, list=58%, signal=198% |
| 389 | GSE13411\_IGM\_MEMORY\_BCELL\_VS\_PLASMA\_CELL\_UP |  | 26 | 0.23 | 1.09 | 0.371 | 1.000 | 1.000 | 264 | tags=31%, list=19%, signal=37% |
| 390 | GSE17721\_POLYIC\_VS\_PAM3CSK4\_8H\_BMDC\_DN |  | 19 | 0.26 | 1.08 | 0.359 | 1.000 | 1.000 | 841 | tags=89%, list=60%, signal=219% |
| 391 | GSE23321\_CD8\_STEM\_CELL\_MEMORY\_VS\_NAIVE\_CD8\_TCELL\_DN |  | 26 | 0.24 | 1.08 | 0.351 | 1.000 | 1.000 | 639 | tags=69%, list=45%, signal=124% |
| 392 | GSE30083\_SP1\_VS\_SP4\_THYMOCYTE\_DN |  | 19 | 0.26 | 1.08 | 0.355 | 1.000 | 1.000 | 355 | tags=42%, list=25%, signal=56% |
| 393 | GSE369\_PRE\_VS\_POST\_IL6\_INJECTION\_IFNG\_WT\_LIVER\_UP |  | 16 | 0.27 | 1.08 | 0.362 | 1.000 | 1.000 | 233 | tags=31%, list=17%, signal=37% |
| 394 | GSE40655\_FOXO1\_KO\_VS\_WT\_NTREG\_DN |  | 17 | 0.27 | 1.08 | 0.348 | 1.000 | 1.000 | 230 | tags=35%, list=16%, signal=42% |
| 395 | GSE9960\_GRAM\_NEG\_VS\_GRAM\_POS\_SEPSIS\_PBMC\_DN |  | 22 | 0.25 | 1.08 | 0.359 | 1.000 | 1.000 | 351 | tags=36%, list=25%, signal=48% |
| 396 | GSE41867\_DAY15\_EFFECTOR\_VS\_DAY30\_MEMORY\_CD8\_TCELL\_LCMV\_ARMSTRONG\_UP |  | 26 | 0.23 | 1.08 | 0.365 | 1.000 | 1.000 | 42 | tags=15%, list=3%, signal=16% |
| 397 | GSE17721\_CTRL\_VS\_CPG\_8H\_BMDC\_DN |  | 21 | 0.25 | 1.08 | 0.353 | 1.000 | 1.000 | 93 | tags=19%, list=7%, signal=20% |
| 398 | GSE8685\_IL2\_STARVED\_VS\_IL15\_ACT\_IL2\_STARVED\_CD4\_TCELL\_DN |  | 16 | 0.27 | 1.08 | 0.362 | 1.000 | 1.000 | 732 | tags=81%, list=52%, signal=167% |
| 399 | GSE21927\_SPLEEN\_C57BL6\_VS\_EL4\_TUMOR\_BALBC\_MONOCYTES\_UP |  | 31 | 0.22 | 1.08 | 0.374 | 1.000 | 1.000 | 400 | tags=42%, list=28%, signal=57% |
| 400 | GSE20727\_H2O2\_VS\_ROS\_INHIBITOR\_TREATED\_DC\_UP |  | 22 | 0.25 | 1.08 | 0.357 | 1.000 | 1.000 | 164 | tags=27%, list=12%, signal=30% |
| 401 | GSE17721\_LPS\_VS\_CPG\_1H\_BMDC\_DN |  | 29 | 0.22 | 1.08 | 0.364 | 1.000 | 1.000 | 209 | tags=24%, list=15%, signal=28% |
| 402 | GSE25087\_FETAL\_VS\_ADULT\_TCONV\_UP |  | 32 | 0.22 | 1.07 | 0.371 | 1.000 | 1.000 | 1063 | tags=97%, list=75%, signal=384% |
| 403 | GSE1740\_MCSF\_VS\_MCSF\_AND\_IFNG\_DAY2\_DERIVED\_MACROPHAGE\_WITH\_IFNA\_STIM\_UP |  | 26 | 0.23 | 1.07 | 0.377 | 1.000 | 1.000 | 225 | tags=27%, list=16%, signal=31% |
| 404 | GSE360\_CTRL\_VS\_B\_MALAYI\_HIGH\_DOSE\_MAC\_UP |  | 20 | 0.26 | 1.07 | 0.355 | 1.000 | 1.000 | 423 | tags=45%, list=30%, signal=63% |
| 405 | GSE10240\_CTRL\_VS\_IL17\_STIM\_PRIMARY\_BRONCHIAL\_EPITHELIAL\_CELLS\_UP |  | 22 | 0.24 | 1.07 | 0.354 | 1.000 | 1.000 | 380 | tags=41%, list=27%, signal=55% |
| 406 | GSE14415\_ACT\_VS\_CTRL\_NATURAL\_TREG\_UP |  | 18 | 0.27 | 1.07 | 0.366 | 1.000 | 1.000 | 1039 | tags=100%, list=74%, signal=374% |
| 407 | GSE33513\_TCF7\_KO\_VS\_HET\_EARLY\_THYMIC\_PROGENITOR\_DN |  | 18 | 0.26 | 1.07 | 0.370 | 1.000 | 1.000 | 257 | tags=39%, list=18%, signal=47% |
| 408 | GSE19401\_PLN\_VS\_PEYERS\_PATCH\_FOLLICULAR\_DC\_UP |  | 18 | 0.26 | 1.07 | 0.353 | 1.000 | 1.000 | 46 | tags=17%, list=3%, signal=17% |
| 409 | GSE14769\_20MIN\_VS\_360MIN\_LPS\_BMDM\_UP |  | 19 | 0.26 | 1.07 | 0.359 | 1.000 | 1.000 | 58 | tags=16%, list=4%, signal=16% |
| 410 | GSE20715\_WT\_VS\_TLR4\_KO\_48H\_OZONE\_LUNG\_DN |  | 20 | 0.25 | 1.07 | 0.384 | 1.000 | 1.000 | 305 | tags=35%, list=22%, signal=44% |
| 411 | GSE27786\_BCELL\_VS\_NEUTROPHIL\_UP |  | 27 | 0.23 | 1.07 | 0.383 | 1.000 | 1.000 | 823 | tags=81%, list=58%, signal=192% |
| 412 | GSE21927\_BALBC\_VS\_C57BL6\_MONOCYTE\_SPLEEN\_DN |  | 16 | 0.27 | 1.07 | 0.380 | 1.000 | 1.000 | 5 | tags=13%, list=0%, signal=12% |
| 413 | GSE29618\_LAIV\_VS\_TIV\_FLU\_VACCINE\_DAY7\_MDC\_UP |  | 24 | 0.24 | 1.07 | 0.373 | 1.000 | 1.000 | 650 | tags=67%, list=46%, signal=122% |
| 414 | GSE17721\_CTRL\_VS\_PAM3CSK4\_2H\_BMDC\_UP |  | 19 | 0.26 | 1.07 | 0.380 | 1.000 | 1.000 | 693 | tags=74%, list=49%, signal=143% |
| 415 | GSE15330\_HSC\_VS\_LYMPHOID\_PRIMED\_MULTIPOTENT\_PROGENITOR\_IKAROS\_KO\_DN |  | 24 | 0.24 | 1.07 | 0.351 | 1.000 | 1.000 | 818 | tags=79%, list=58%, signal=185% |
| 416 | GSE25087\_FETAL\_VS\_ADULT\_TREG\_UP |  | 28 | 0.22 | 1.07 | 0.389 | 1.000 | 1.000 | 335 | tags=32%, list=24%, signal=41% |
| 417 | GSE14413\_UNSTIM\_VS\_IFNB\_STIM\_NIH3T3\_CELLS\_DN |  | 19 | 0.26 | 1.07 | 0.381 | 1.000 | 1.000 | 189 | tags=26%, list=13%, signal=30% |
| 418 | GSE5503\_LIVER\_DC\_VS\_PLN\_DC\_ACTIVATED\_ALLOGENIC\_TCELL\_DN |  | 26 | 0.23 | 1.07 | 0.363 | 1.000 | 1.000 | 507 | tags=58%, list=36%, signal=88% |
| 419 | GSE37416\_CTRL\_VS\_24H\_F\_TULARENSIS\_LVS\_NEUTROPHIL\_UP |  | 24 | 0.23 | 1.07 | 0.353 | 1.000 | 1.000 | 302 | tags=38%, list=21%, signal=47% |
| 420 | GSE27291\_6H\_VS\_7D\_STIM\_GAMMADELTA\_TCELL\_DN |  | 20 | 0.25 | 1.07 | 0.369 | 1.000 | 1.000 | 556 | tags=60%, list=39%, signal=98% |
| 421 | GSE17721\_POLYIC\_VS\_CPG\_16H\_BMDC\_UP |  | 18 | 0.26 | 1.06 | 0.392 | 1.000 | 1.000 | 77 | tags=17%, list=5%, signal=17% |
| 422 | GSE46143\_CTRL\_VS\_LMP2A\_TRANSDUCED\_CD10\_POS\_GC\_BCELL\_DN |  | 23 | 0.24 | 1.06 | 0.386 | 1.000 | 1.000 | 436 | tags=43%, list=31%, signal=62% |
| 423 | GSE22229\_RENAL\_TRANSPLANT\_IMMUNOSUPP\_THERAPY\_VS\_HEALTHY\_PBMC\_UP |  | 18 | 0.26 | 1.06 | 0.381 | 1.000 | 1.000 | 332 | tags=39%, list=24%, signal=50% |
| 424 | GSE18791\_UNSTIM\_VS\_NEWCATSLE\_VIRUS\_DC\_18H\_UP |  | 18 | 0.26 | 1.06 | 0.385 | 1.000 | 1.000 | 658 | tags=72%, list=47%, signal=134% |
| 425 | GSE17721\_0.5H\_VS\_4H\_PAM3CSK4\_BMDC\_DN |  | 21 | 0.25 | 1.06 | 0.377 | 1.000 | 1.000 | 355 | tags=38%, list=25%, signal=50% |
| 426 | GSE6674\_UNSTIM\_VS\_ANTI\_IGM\_AND\_CPG\_STIM\_BCELL\_DN |  | 30 | 0.22 | 1.06 | 0.392 | 1.000 | 1.000 | 690 | tags=70%, list=49%, signal=134% |
| 427 | GSE14308\_TH2\_VS\_TH17\_UP |  | 17 | 0.27 | 1.06 | 0.378 | 1.000 | 1.000 | 1036 | tags=100%, list=73%, signal=372% |
| 428 | GSE9509\_10MIN\_VS\_30MIN\_LPS\_AND\_IL10\_STIM\_IL10\_KO\_MACROPHAGE\_DN |  | 16 | 0.27 | 1.06 | 0.393 | 1.000 | 1.000 | 313 | tags=38%, list=22%, signal=48% |
| 429 | GSE5589\_WT\_VS\_IL10\_KO\_LPS\_STIM\_MACROPHAGE\_45MIN\_DN |  | 28 | 0.22 | 1.06 | 0.371 | 1.000 | 1.000 | 286 | tags=32%, list=20%, signal=40% |
| 430 | GSE11961\_FOLLICULAR\_BCELL\_VS\_PLASMA\_CELL\_DAY7\_UP |  | 21 | 0.25 | 1.06 | 0.405 | 1.000 | 1.000 | 560 | tags=52%, list=40%, signal=86% |
| 431 | GSE9037\_WT\_VS\_IRAK4\_KO\_BMDM\_UP |  | 27 | 0.23 | 1.05 | 0.361 | 1.000 | 1.000 | 209 | tags=26%, list=15%, signal=30% |
| 432 | GSE19888\_ADENOSINE\_A3R\_INH\_VS\_TCELL\_MEMBRANES\_ACT\_MAST\_CELL\_UP |  | 21 | 0.24 | 1.05 | 0.393 | 1.000 | 1.000 | 323 | tags=33%, list=23%, signal=43% |
| 433 | GSE17721\_CPG\_VS\_GARDIQUIMOD\_0.5H\_BMDC\_DN |  | 16 | 0.27 | 1.05 | 0.398 | 1.000 | 1.000 | 587 | tags=63%, list=42%, signal=106% |
| 434 | GSE7460\_WT\_VS\_FOXP3\_HET\_ACT\_WITH\_TGFB\_TCONV\_UP |  | 24 | 0.23 | 1.05 | 0.397 | 1.000 | 1.000 | 373 | tags=42%, list=26%, signal=56% |
| 435 | GSE21670\_TGFB\_VS\_TGFB\_AND\_IL6\_TREATED\_CD4\_TCELL\_UP |  | 17 | 0.27 | 1.05 | 0.384 | 1.000 | 1.000 | 306 | tags=35%, list=22%, signal=45% |
| 436 | GSE29618\_PRE\_VS\_DAY7\_POST\_TIV\_FLU\_VACCINE\_PDC\_DN |  | 27 | 0.23 | 1.05 | 0.396 | 1.000 | 1.000 | 800 | tags=78%, list=57%, signal=176% |
| 437 | GSE17721\_CPG\_VS\_GARDIQUIMOD\_1H\_BMDC\_DN |  | 21 | 0.24 | 1.05 | 0.387 | 1.000 | 1.000 | 713 | tags=76%, list=51%, signal=152% |
| 438 | GSE37532\_TREG\_VS\_TCONV\_CD4\_TCELL\_FROM\_LN\_UP |  | 34 | 0.21 | 1.05 | 0.382 | 1.000 | 1.000 | 890 | tags=88%, list=63%, signal=233% |
| 439 | GSE14308\_NAIVE\_CD4\_TCELL\_VS\_INDUCED\_TREG\_DN |  | 21 | 0.24 | 1.05 | 0.397 | 1.000 | 1.000 | 671 | tags=71%, list=48%, signal=134% |
| 440 | GSE2405\_0H\_VS\_1.5H\_A\_PHAGOCYTOPHILUM\_STIM\_NEUTROPHIL\_DN |  | 17 | 0.27 | 1.05 | 0.409 | 1.000 | 1.000 | 607 | tags=65%, list=43%, signal=112% |
| 441 | GSE36078\_UNTREATED\_VS\_AD5\_T425A\_HEXON\_INF\_MOUSE\_LUNG\_DC\_UP |  | 26 | 0.23 | 1.05 | 0.395 | 1.000 | 1.000 | 393 | tags=42%, list=28%, signal=58% |
| 442 | GSE5589\_LPS\_VS\_LPS\_AND\_IL10\_STIM\_MACROPHAGE\_45MIN\_UP |  | 23 | 0.24 | 1.05 | 0.397 | 1.000 | 1.000 | 432 | tags=48%, list=31%, signal=68% |
| 443 | GSE45365\_WT\_VS\_IFNAR\_KO\_BCELL\_MCMV\_INFECTION\_DN |  | 17 | 0.26 | 1.05 | 0.400 | 1.000 | 1.000 | 13 | tags=12%, list=1%, signal=12% |
| 444 | GSE36078\_UNTREATED\_VS\_AD5\_T425A\_HEXON\_INF\_IL1R\_KO\_MOUSE\_LUNG\_DC\_UP |  | 18 | 0.26 | 1.05 | 0.403 | 1.000 | 1.000 | 631 | tags=67%, list=45%, signal=119% |
| 445 | GSE3039\_ALPHABETA\_CD8\_TCELL\_VS\_B2\_BCELL\_UP |  | 18 | 0.26 | 1.05 | 0.405 | 1.000 | 1.000 | 373 | tags=44%, list=26%, signal=60% |
| 446 | GSE5589\_LPS\_VS\_LPS\_AND\_IL10\_STIM\_MACROPHAGE\_45MIN\_DN |  | 16 | 0.27 | 1.04 | 0.394 | 1.000 | 1.000 | 414 | tags=50%, list=29%, signal=70% |
| 447 | GSE40274\_XBP1\_VS\_FOXP3\_AND\_XBP1\_TRANSDUCED\_ACTIVATED\_CD4\_TCELL\_DN |  | 28 | 0.22 | 1.04 | 0.399 | 1.000 | 1.000 | 796 | tags=79%, list=56%, signal=177% |
| 448 | GSE6259\_33D1\_POS\_DC\_VS\_CD4\_TCELL\_DN |  | 17 | 0.26 | 1.04 | 0.400 | 1.000 | 1.000 | 174 | tags=24%, list=12%, signal=27% |
| 449 | GSE13485\_CTRL\_VS\_DAY3\_YF17D\_VACCINE\_PBMC\_DN |  | 22 | 0.24 | 1.04 | 0.386 | 1.000 | 1.000 | 382 | tags=41%, list=27%, signal=55% |
| 450 | GSE22935\_24H\_VS\_48H\_MBOVIS\_BCG\_STIM\_MYD88\_KO\_MACROPHAGE\_UP |  | 21 | 0.24 | 1.04 | 0.408 | 1.000 | 1.000 | 57 | tags=14%, list=4%, signal=15% |
| 451 | GSE17721\_LPS\_VS\_CPG\_12H\_BMDC\_UP |  | 15 | 0.27 | 1.04 | 0.404 | 1.000 | 1.000 | 285 | tags=40%, list=20%, signal=50% |
| 452 | GSE23505\_IL6\_IL1\_VS\_IL6\_IL1\_IL23\_TREATED\_CD4\_TCELL\_UP |  | 19 | 0.25 | 1.04 | 0.402 | 1.000 | 1.000 | 285 | tags=32%, list=20%, signal=39% |
| 453 | GSE7460\_WT\_VS\_FOXP3\_HET\_ACT\_TCONV\_DN |  | 15 | 0.27 | 1.04 | 0.401 | 1.000 | 1.000 | 470 | tags=60%, list=33%, signal=89% |
| 454 | GSE18791\_UNSTIM\_VS\_NEWCATSLE\_VIRUS\_DC\_6H\_DN |  | 25 | 0.22 | 1.04 | 0.421 | 1.000 | 1.000 | 263 | tags=32%, list=19%, signal=39% |
| 455 | GSE32423\_MEMORY\_VS\_NAIVE\_CD8\_TCELL\_IL7\_IL4\_DN |  | 20 | 0.25 | 1.04 | 0.421 | 1.000 | 1.000 | 456 | tags=50%, list=32%, signal=73% |
| 456 | GSE3982\_MAST\_CELL\_VS\_NEUTROPHIL\_UP |  | 20 | 0.24 | 1.03 | 0.401 | 1.000 | 1.000 | 428 | tags=45%, list=30%, signal=64% |
| 457 | GSE36009\_UNSTIM\_VS\_LPS\_STIM\_NLRP10\_KO\_DC\_DN |  | 27 | 0.22 | 1.03 | 0.423 | 1.000 | 1.000 | 448 | tags=44%, list=32%, signal=64% |
| 458 | GSE28737\_WT\_VS\_BCL6\_HET\_FOLLICULAR\_BCELL\_UP |  | 18 | 0.25 | 1.03 | 0.406 | 1.000 | 1.000 | 556 | tags=61%, list=39%, signal=100% |
| 459 | GSE5142\_HTERT\_TRANSDUCED\_VS\_CTRL\_CD8\_TCELL\_LATE\_PASSAGE\_CLONE\_UP |  | 27 | 0.22 | 1.03 | 0.412 | 1.000 | 1.000 | 563 | tags=52%, list=40%, signal=85% |
| 460 | GSE22611\_NOD2\_VS\_MUTANT\_NOD2\_TRANSDUCED\_HEK293T\_CELL\_UP |  | 20 | 0.24 | 1.03 | 0.418 | 1.000 | 1.000 | 640 | tags=65%, list=45%, signal=117% |
| 461 | GSE14415\_ACT\_TCONV\_VS\_ACT\_NATURAL\_TREG\_UP |  | 15 | 0.27 | 1.03 | 0.413 | 1.000 | 1.000 | 193 | tags=27%, list=14%, signal=31% |
| 462 | GSE21033\_CTRL\_VS\_POLYIC\_STIM\_DC\_12H\_DN |  | 26 | 0.22 | 1.03 | 0.416 | 1.000 | 1.000 | 257 | tags=31%, list=18%, signal=37% |
| 463 | GSE22611\_NOD2\_VS\_CTRL\_TRANSDUCED\_HEK293T\_CELL\_UP |  | 20 | 0.25 | 1.03 | 0.404 | 1.000 | 1.000 | 55 | tags=15%, list=4%, signal=15% |
| 464 | GSE2770\_UNTREATED\_VS\_IL12\_TREATED\_ACT\_CD4\_TCELL\_6H\_DN |  | 22 | 0.23 | 1.03 | 0.425 | 1.000 | 1.000 | 531 | tags=59%, list=38%, signal=93% |
| 465 | GSE5679\_CTRL\_VS\_PPARG\_LIGAND\_ROSIGLITAZONE\_TREATED\_DC\_UP |  | 23 | 0.23 | 1.03 | 0.430 | 1.000 | 1.000 | 833 | tags=83%, list=59%, signal=198% |
| 466 | GSE17186\_CD21LOW\_VS\_CD21HIGH\_TRANSITIONAL\_BCELL\_CORD\_BLOOD\_DN |  | 23 | 0.23 | 1.03 | 0.421 | 1.000 | 1.000 | 256 | tags=30%, list=18%, signal=37% |
| 467 | GSE40274\_CTRL\_VS\_FOXP3\_AND\_HELIOS\_TRANSDUCED\_ACTIVATED\_CD4\_TCELL\_UP |  | 27 | 0.21 | 1.03 | 0.434 | 1.000 | 1.000 | 600 | tags=56%, list=43%, signal=95% |
| 468 | GSE18893\_CTRL\_VS\_TNF\_TREATED\_TCONV\_24H\_UP |  | 19 | 0.25 | 1.03 | 0.408 | 1.000 | 1.000 | 278 | tags=37%, list=20%, signal=45% |
| 469 | GSE25088\_CTRL\_VS\_IL4\_AND\_ROSIGLITAZONE\_STIM\_STAT6\_KO\_MACROPHAGE\_UP |  | 33 | 0.21 | 1.03 | 0.408 | 1.000 | 1.000 | 247 | tags=27%, list=18%, signal=32% |
| 470 | GSE17721\_POLYIC\_VS\_CPG\_4H\_BMDC\_DN |  | 23 | 0.23 | 1.02 | 0.424 | 1.000 | 1.000 | 243 | tags=30%, list=17%, signal=36% |
| 471 | GSE16385\_MONOCYTE\_VS\_12H\_IFNG\_TNF\_TREATED\_MACROPHAGE\_DN |  | 24 | 0.23 | 1.02 | 0.431 | 1.000 | 1.000 | 193 | tags=25%, list=14%, signal=28% |
| 472 | GSE32986\_GMCSF\_VS\_GMCSF\_AND\_CURDLAN\_HIGHDOSE\_STIM\_DC\_DN |  | 24 | 0.23 | 1.02 | 0.421 | 1.000 | 1.000 | 166 | tags=25%, list=12%, signal=28% |
| 473 | GSE21546\_ELK1\_KO\_VS\_SAP1A\_KO\_AND\_ELK1\_KO\_DP\_THYMOCYTES\_DN |  | 24 | 0.23 | 1.02 | 0.457 | 1.000 | 1.000 | 837 | tags=83%, list=59%, signal=201% |
| 474 | GSE40274\_CTRL\_VS\_SATB1\_TRANSDUCED\_ACTIVATED\_CD4\_TCELL\_UP |  | 18 | 0.25 | 1.02 | 0.420 | 1.000 | 1.000 | 185 | tags=28%, list=13%, signal=32% |
| 475 | GSE34156\_UNTREATED\_VS\_24H\_NOD2\_LIGAND\_TREATED\_MONOCYTE\_DN |  | 18 | 0.25 | 1.02 | 0.442 | 1.000 | 1.000 | 240 | tags=28%, list=17%, signal=33% |
| 476 | GSE22589\_HIV\_VS\_HIV\_AND\_SIV\_INFECTED\_DC\_DN |  | 30 | 0.21 | 1.02 | 0.429 | 1.000 | 1.000 | 616 | tags=57%, list=44%, signal=98% |
| 477 | GSE17721\_PAM3CSK4\_VS\_CPG\_6H\_BMDC\_UP |  | 22 | 0.23 | 1.01 | 0.425 | 1.000 | 1.000 | 895 | tags=91%, list=63%, signal=245% |
| 478 | GSE5679\_CTRL\_VS\_RARA\_AGONIST\_AM580\_TREATED\_DC\_UP |  | 26 | 0.22 | 1.01 | 0.440 | 1.000 | 1.000 | 837 | tags=81%, list=59%, signal=195% |
| 479 | GSE27859\_MACROPHAGE\_VS\_CD11C\_INT\_F480\_INT\_DC\_DN |  | 19 | 0.24 | 1.01 | 0.439 | 1.000 | 1.000 | 728 | tags=74%, list=52%, signal=150% |
| 480 | GSE37532\_WT\_VS\_PPARG\_KO\_LN\_TREG\_UP |  | 25 | 0.22 | 1.01 | 0.442 | 1.000 | 1.000 | 229 | tags=28%, list=16%, signal=33% |
| 481 | GSE43955\_TH0\_VS\_TGFB\_IL6\_TH17\_ACT\_CD4\_TCELL\_60H\_UP |  | 25 | 0.21 | 1.01 | 0.431 | 1.000 | 1.000 | 376 | tags=36%, list=27%, signal=48% |
| 482 | GSE11924\_TFH\_VS\_TH17\_CD4\_TCELL\_UP |  | 18 | 0.25 | 1.01 | 0.450 | 1.000 | 1.000 | 664 | tags=67%, list=47%, signal=124% |
| 483 | GSE18804\_SPLEEN\_MACROPHAGE\_VS\_TUMORAL\_MACROPHAGE\_UP |  | 16 | 0.26 | 1.01 | 0.425 | 1.000 | 1.000 | 432 | tags=44%, list=31%, signal=62% |
| 484 | GSE11864\_UNTREATED\_VS\_CSF1\_IFNG\_PAM3CYS\_IN\_MAC\_DN |  | 16 | 0.26 | 1.01 | 0.453 | 1.000 | 1.000 | 377 | tags=50%, list=27%, signal=67% |
| 485 | GSE45365\_CTRL\_VS\_MCMV\_INFECTION\_NK\_CELL\_DN |  | 18 | 0.25 | 1.01 | 0.437 | 1.000 | 1.000 | 534 | tags=56%, list=38%, signal=88% |
| 486 | GSE17974\_0H\_VS\_2H\_IN\_VITRO\_ACT\_CD4\_TCELL\_DN |  | 33 | 0.20 | 1.01 | 0.444 | 1.000 | 1.000 | 222 | tags=24%, list=16%, signal=28% |
| 487 | GSE17721\_LPS\_VS\_PAM3CSK4\_0.5H\_BMDC\_DN |  | 19 | 0.24 | 1.01 | 0.454 | 1.000 | 1.000 | 77 | tags=16%, list=5%, signal=16% |
| 488 | GSE11961\_MEMORY\_BCELL\_DAY7\_VS\_MEMORY\_BCELL\_DAY40\_DN |  | 17 | 0.25 | 1.01 | 0.455 | 1.000 | 1.000 | 161 | tags=24%, list=11%, signal=26% |
| 489 | GSE17721\_LPS\_VS\_PAM3CSK4\_0.5H\_BMDC\_UP |  | 15 | 0.27 | 1.01 | 0.455 | 1.000 | 1.000 | 172 | tags=27%, list=12%, signal=30% |
| 490 | GSE27786\_NKCELL\_VS\_NEUTROPHIL\_UP |  | 28 | 0.22 | 1.01 | 0.442 | 1.000 | 1.000 | 801 | tags=75%, list=57%, signal=170% |
| 491 | GSE26030\_TH1\_VS\_TH17\_RESTIMULATED\_DAY5\_POST\_POLARIZATION\_DN |  | 20 | 0.24 | 1.01 | 0.437 | 1.000 | 1.000 | 204 | tags=25%, list=14%, signal=29% |
| 492 | GSE16385\_UNTREATED\_VS\_12H\_ROSIGLITAZONE\_IL4\_TREATED\_MACROPHAGE\_DN |  | 20 | 0.24 | 1.01 | 0.446 | 1.000 | 1.000 | 646 | tags=70%, list=46%, signal=127% |
| 493 | GSE3565\_DUSP1\_VS\_WT\_SPLENOCYTES\_POST\_LPS\_INJECTION\_UP |  | 16 | 0.26 | 1.01 | 0.423 | 1.000 | 1.000 | 485 | tags=50%, list=34%, signal=75% |
| 494 | GSE46606\_UNSTIM\_VS\_CD40L\_IL2\_IL5\_DAY1\_STIMULATED\_BCELL\_UP |  | 22 | 0.23 | 1.01 | 0.449 | 1.000 | 1.000 | 314 | tags=36%, list=22%, signal=46% |
| 495 | GSE3982\_BASOPHIL\_VS\_EFF\_MEMORY\_CD4\_TCELL\_DN |  | 15 | 0.26 | 1.00 | 0.444 | 1.000 | 1.000 | 715 | tags=73%, list=51%, signal=147% |
| 496 | GSE2770\_IL12\_AND\_TGFB\_VS\_IL4\_TREATED\_ACT\_CD4\_TCELL\_2H\_UP |  | 25 | 0.22 | 1.00 | 0.457 | 1.000 | 1.000 | 425 | tags=40%, list=30%, signal=56% |
| 497 | GSE5503\_MLN\_DC\_VS\_SPLEEN\_DC\_ACTIVATED\_ALLOGENIC\_TCELL\_DN |  | 29 | 0.21 | 1.00 | 0.473 | 1.000 | 1.000 | 596 | tags=55%, list=42%, signal=94% |
| 498 | GSE11924\_TH1\_VS\_TH17\_CD4\_TCELL\_UP |  | 15 | 0.26 | 1.00 | 0.461 | 1.000 | 1.000 | 199 | tags=27%, list=14%, signal=31% |
| 499 | GSE22432\_CONVENTIONAL\_CDC\_VS\_PLASMACYTOID\_PDC\_UP |  | 22 | 0.23 | 1.00 | 0.478 | 1.000 | 1.000 | 754 | tags=77%, list=53%, signal=163% |
| 500 | GSE25088\_WT\_VS\_STAT6\_KO\_MACROPHAGE\_ROSIGLITAZONE\_STIM\_UP |  | 23 | 0.22 | 1.00 | 0.437 | 1.000 | 1.000 | 78 | tags=17%, list=6%, signal=18% |
| 501 | GSE10240\_IL17\_VS\_IL17\_AND\_IL22\_STIM\_PRIMARY\_BRONCHIAL\_EPITHELIAL\_CELLS\_UP |  | 23 | 0.22 | 1.00 | 0.461 | 1.000 | 1.000 | 292 | tags=35%, list=21%, signal=43% |
| 502 | GSE15330\_MEGAKARYOCYTE\_ERYTHROID\_PROGENITOR\_VS\_PRO\_BCELL\_DN |  | 17 | 0.25 | 1.00 | 0.456 | 1.000 | 1.000 | 666 | tags=71%, list=47%, signal=132% |
| 503 | GSE25123\_WT\_VS\_PPARG\_KO\_MACROPHAGE\_ROSIGLITAZONE\_STIM\_UP |  | 28 | 0.21 | 1.00 | 0.460 | 1.000 | 1.000 | 464 | tags=43%, list=33%, signal=63% |
| 504 | GSE28783\_ANTI\_MIR33\_VS\_UNTREATED\_ATHEROSCLEROSIS\_MACROPHAGE\_UP |  | 18 | 0.24 | 1.00 | 0.450 | 1.000 | 1.000 | 329 | tags=39%, list=23%, signal=50% |
| 505 | GSE3982\_CTRL\_VS\_LPS\_1H\_NEUTROPHIL\_UP |  | 22 | 0.22 | 1.00 | 0.464 | 1.000 | 1.000 | 555 | tags=55%, list=39%, signal=89% |
| 506 | GSE24634\_NAIVE\_CD4\_TCELL\_VS\_DAY5\_IL4\_CONV\_TREG\_DN |  | 26 | 0.21 | 1.00 | 0.471 | 1.000 | 1.000 | 574 | tags=58%, list=41%, signal=95% |
| 507 | GSE46468\_LUNG\_INNATE\_LYMPHOID\_CELL\_VS\_SPLEEN\_CD4\_TCELL\_DN |  | 34 | 0.19 | 1.00 | 0.465 | 1.000 | 1.000 | 816 | tags=76%, list=58%, signal=177% |
| 508 | GSE17974\_0H\_VS\_12H\_IN\_VITRO\_ACT\_CD4\_TCELL\_DN |  | 27 | 0.21 | 1.00 | 0.455 | 1.000 | 1.000 | 947 | tags=93%, list=67%, signal=276% |
| 509 | GSE17721\_POLYIC\_VS\_PAM3CSK4\_2H\_BMDC\_DN |  | 18 | 0.24 | 1.00 | 0.457 | 1.000 | 1.000 | 5 | tags=11%, list=0%, signal=11% |
| 510 | GSE13306\_RA\_VS\_UNTREATED\_MEM\_CD4\_TCELL\_UP |  | 21 | 0.23 | 0.99 | 0.477 | 1.000 | 1.000 | 558 | tags=62%, list=40%, signal=101% |
| 511 | GSE45365\_WT\_VS\_IFNAR\_KO\_CD11B\_DC\_MCMV\_INFECTION\_UP |  | 22 | 0.23 | 0.99 | 0.452 | 1.000 | 1.000 | 627 | tags=59%, list=44%, signal=105% |
| 512 | GSE3982\_MAST\_CELL\_VS\_DC\_UP |  | 20 | 0.24 | 0.99 | 0.439 | 1.000 | 1.000 | 151 | tags=20%, list=11%, signal=22% |
| 513 | GSE4142\_NAIVE\_BCELL\_VS\_PLASMA\_CELL\_DN |  | 15 | 0.26 | 0.99 | 0.456 | 1.000 | 1.000 | 506 | tags=53%, list=36%, signal=82% |
| 514 | GSE18893\_TCONV\_VS\_TREG\_24H\_TNF\_STIM\_UP |  | 18 | 0.24 | 0.99 | 0.470 | 1.000 | 1.000 | 888 | tags=89%, list=63%, signal=237% |
| 515 | GSE360\_DC\_VS\_MAC\_B\_MALAYI\_LOW\_DOSE\_UP |  | 21 | 0.23 | 0.98 | 0.488 | 1.000 | 1.000 | 409 | tags=38%, list=29%, signal=53% |
| 516 | GSE27786\_CD8\_TCELL\_VS\_NKCELL\_UP |  | 18 | 0.25 | 0.98 | 0.487 | 1.000 | 1.000 | 372 | tags=44%, list=26%, signal=60% |
| 517 | GSE34156\_UNTREATED\_VS\_24H\_TLR1\_TLR2\_LIGAND\_TREATED\_MONOCYTE\_DN |  | 18 | 0.24 | 0.98 | 0.473 | 1.000 | 1.000 | 431 | tags=44%, list=31%, signal=63% |
| 518 | GSE36826\_NORMAL\_VS\_STAPH\_AUREUS\_INF\_SKIN\_DN |  | 21 | 0.22 | 0.98 | 0.476 | 1.000 | 1.000 | 256 | tags=29%, list=18%, signal=34% |
| 519 | GSE17721\_CTRL\_VS\_PAM3CSK4\_12H\_BMDC\_DN |  | 18 | 0.24 | 0.98 | 0.480 | 1.000 | 1.000 | 93 | tags=17%, list=7%, signal=18% |
| 520 | GSE22611\_UNSTIM\_VS\_2H\_MDP\_STIM\_NOD2\_TRANSDUCED\_HEK293T\_CELL\_DN |  | 18 | 0.25 | 0.98 | 0.491 | 1.000 | 1.000 | 449 | tags=50%, list=32%, signal=72% |
| 521 | GSE8921\_UNSTIM\_0H\_VS\_TLR1\_2\_STIM\_MONOCYTE\_12H\_UP |  | 18 | 0.24 | 0.98 | 0.476 | 1.000 | 1.000 | 451 | tags=44%, list=32%, signal=64% |
| 522 | GSE22886\_CD8\_VS\_CD4\_NAIVE\_TCELL\_UP |  | 21 | 0.23 | 0.98 | 0.491 | 1.000 | 1.000 | 710 | tags=67%, list=50%, signal=132% |
| 523 | GSE14308\_NAIVE\_CD4\_TCELL\_VS\_INDUCED\_TREG\_UP |  | 17 | 0.24 | 0.98 | 0.475 | 1.000 | 1.000 | 467 | tags=47%, list=33%, signal=69% |
| 524 | GSE37534\_PIOGLITAZONE\_VS\_ROSIGLITAZONE\_TREATED\_CD4\_TCELL\_PPARG1\_FOXP3\_TRANSDUCED\_UP |  | 17 | 0.25 | 0.98 | 0.483 | 1.000 | 1.000 | 741 | tags=71%, list=53%, signal=147% |
| 525 | GSE3982\_CTRL\_VS\_LPS\_48H\_DC\_UP |  | 21 | 0.23 | 0.98 | 0.477 | 1.000 | 1.000 | 359 | tags=43%, list=25%, signal=57% |
| 526 | GSE27241\_WT\_VS\_RORGT\_KO\_TH17\_POLARIZED\_CD4\_TCELL\_TREATED\_WITH\_DIGOXIN\_UP |  | 19 | 0.23 | 0.98 | 0.509 | 1.000 | 1.000 | 177 | tags=26%, list=13%, signal=30% |
| 527 | GSE8921\_UNSTIM\_0H\_VS\_TLR1\_2\_STIM\_MONOCYTE\_12H\_DN |  | 22 | 0.22 | 0.97 | 0.485 | 1.000 | 1.000 | 84 | tags=18%, list=6%, signal=19% |
| 528 | GSE3982\_EOSINOPHIL\_VS\_TH1\_DN |  | 28 | 0.20 | 0.97 | 0.486 | 1.000 | 1.000 | 868 | tags=86%, list=62%, signal=218% |
| 529 | GSE22282\_HYPOXIA\_VS\_NORMOXIA\_MYELOID\_DC\_DN |  | 15 | 0.26 | 0.97 | 0.487 | 1.000 | 1.000 | 287 | tags=40%, list=20%, signal=50% |
| 530 | GSE1112\_OT1\_CD8AB\_VS\_HY\_CD8AA\_THYMOCYTE\_RTOC\_CULTURE\_DN |  | 15 | 0.26 | 0.97 | 0.502 | 1.000 | 1.000 | 562 | tags=60%, list=40%, signal=99% |
| 531 | GSE3982\_CTRL\_VS\_PMA\_STIM\_EOSINOPHIL\_UP |  | 17 | 0.25 | 0.97 | 0.486 | 1.000 | 1.000 | 562 | tags=59%, list=40%, signal=97% |
| 532 | GSE20500\_CTRL\_VS\_RARA\_ANTAGONIST\_TREATED\_CD4\_TCELL\_DN |  | 28 | 0.20 | 0.97 | 0.501 | 1.000 | 1.000 | 665 | tags=61%, list=47%, signal=113% |
| 533 | GSE27786\_ERYTHROBLAST\_VS\_MONO\_MAC\_UP |  | 19 | 0.23 | 0.97 | 0.492 | 1.000 | 1.000 | 124 | tags=21%, list=9%, signal=23% |
| 534 | GSE36009\_UNSTIM\_VS\_LPS\_STIM\_NLRP10\_KO\_DC\_UP |  | 24 | 0.21 | 0.97 | 0.487 | 1.000 | 1.000 | 450 | tags=42%, list=32%, signal=60% |
| 535 | GSE21360\_SECONDARY\_VS\_TERTIARY\_MEMORY\_CD8\_TCELL\_UP |  | 18 | 0.24 | 0.97 | 0.503 | 1.000 | 1.000 | 265 | tags=28%, list=19%, signal=34% |
| 536 | GSE14908\_RESTING\_VS\_HDM\_STIM\_CD4\_TCELL\_NONATOPIC\_PATIENT\_UP |  | 17 | 0.25 | 0.97 | 0.503 | 1.000 | 1.000 | 58 | tags=18%, list=4%, signal=18% |
| 537 | GSE30083\_SP3\_VS\_SP4\_THYMOCYTE\_DN |  | 18 | 0.24 | 0.97 | 0.493 | 1.000 | 1.000 | 245 | tags=28%, list=17%, signal=33% |
| 538 | GSE26488\_CTRL\_VS\_PEPTIDE\_INJECTION\_HDAC7\_DELTAP\_TG\_OT2\_THYMOCYTE\_DN |  | 17 | 0.24 | 0.97 | 0.495 | 1.000 | 1.000 | 561 | tags=53%, list=40%, signal=87% |
| 539 | GSE360\_L\_DONOVANI\_VS\_L\_MAJOR\_DC\_UP |  | 18 | 0.24 | 0.97 | 0.503 | 1.000 | 1.000 | 337 | tags=39%, list=24%, signal=50% |
| 540 | GSE20366\_EX\_VIVO\_VS\_DEC205\_CONVERSION\_NAIVE\_CD4\_TCELL\_UP |  | 20 | 0.23 | 0.97 | 0.511 | 1.000 | 1.000 | 352 | tags=40%, list=25%, signal=53% |
| 541 | GSE46606\_IRF4MID\_VS\_WT\_CD40L\_IL2\_IL5\_DAY3\_STIMULATED\_BCELL\_DN |  | 16 | 0.25 | 0.97 | 0.479 | 1.000 | 1.000 | 284 | tags=31%, list=20%, signal=39% |
| 542 | GSE4142\_GC\_BCELL\_VS\_MEMORY\_BCELL\_DN |  | 20 | 0.23 | 0.97 | 0.515 | 1.000 | 1.000 | 268 | tags=30%, list=19%, signal=37% |
| 543 | GSE19401\_UNSTIM\_VS\_PAM2CSK4\_STIM\_FOLLICULAR\_DC\_DN |  | 23 | 0.21 | 0.97 | 0.508 | 1.000 | 1.000 | 202 | tags=26%, list=14%, signal=30% |
| 544 | GSE3982\_DC\_VS\_CENT\_MEMORY\_CD4\_TCELL\_DN |  | 18 | 0.24 | 0.97 | 0.505 | 1.000 | 1.000 | 62 | tags=17%, list=4%, signal=17% |
| 545 | GSE31082\_DN\_VS\_DP\_THYMOCYTE\_UP |  | 20 | 0.23 | 0.96 | 0.485 | 1.000 | 1.000 | 856 | tags=85%, list=61%, signal=213% |
| 546 | GSE18281\_CORTEX\_VS\_MEDULLA\_THYMUS\_UP |  | 17 | 0.24 | 0.96 | 0.501 | 1.000 | 1.000 | 394 | tags=41%, list=28%, signal=56% |
| 547 | GSE17721\_PAM3CSK4\_VS\_CPG\_24H\_BMDC\_UP |  | 16 | 0.25 | 0.96 | 0.486 | 1.000 | 1.000 | 92 | tags=19%, list=7%, signal=20% |
| 548 | GSE21670\_UNTREATED\_VS\_TGFB\_TREATED\_CD4\_TCELL\_DN |  | 19 | 0.23 | 0.96 | 0.518 | 1.000 | 1.000 | 129 | tags=21%, list=9%, signal=23% |
| 549 | GSE23308\_WT\_VS\_MINERALCORTICOID\_REC\_KO\_MACROPHAGE\_CORTICOSTERONE\_TREATED\_DN |  | 23 | 0.22 | 0.96 | 0.497 | 1.000 | 1.000 | 63 | tags=13%, list=4%, signal=13% |
| 550 | GSE21670\_UNTREATED\_VS\_IL6\_TREATED\_CD4\_TCELL\_UP |  | 18 | 0.23 | 0.96 | 0.503 | 1.000 | 1.000 | 69 | tags=17%, list=5%, signal=17% |
| 551 | GSE9006\_HEALTHY\_VS\_TYPE\_1\_DIABETES\_PBMC\_1MONTH\_POST\_DX\_DN |  | 16 | 0.25 | 0.96 | 0.518 | 1.000 | 1.000 | 686 | tags=69%, list=49%, signal=132% |
| 552 | GSE22886\_NAIVE\_CD4\_TCELL\_VS\_NEUTROPHIL\_UP |  | 23 | 0.21 | 0.96 | 0.516 | 1.000 | 1.000 | 208 | tags=26%, list=15%, signal=30% |
| 553 | GSE17721\_PAM3CSK4\_VS\_GADIQUIMOD\_8H\_BMDC\_DN |  | 26 | 0.21 | 0.96 | 0.477 | 1.000 | 1.000 | 493 | tags=50%, list=35%, signal=75% |
| 554 | GSE20366\_CD103\_KLRG1\_DP\_VS\_DN\_TREG\_UP |  | 17 | 0.24 | 0.96 | 0.516 | 1.000 | 1.000 | 391 | tags=41%, list=28%, signal=56% |
| 555 | GSE24814\_STAT5\_KO\_VS\_WT\_PRE\_BCELL\_UP |  | 16 | 0.24 | 0.96 | 0.494 | 1.000 | 1.000 | 99 | tags=19%, list=7%, signal=20% |
| 556 | GSE2124\_CTRL\_VS\_LYMPHOTOXIN\_BETA\_TREATED\_MLN\_DN |  | 17 | 0.24 | 0.96 | 0.510 | 1.000 | 1.000 | 302 | tags=35%, list=21%, signal=44% |
| 557 | GSE22886\_NAIVE\_CD8\_TCELL\_VS\_NEUTROPHIL\_UP |  | 19 | 0.23 | 0.96 | 0.515 | 1.000 | 1.000 | 470 | tags=47%, list=33%, signal=70% |
| 558 | GSE12507\_PDC\_CELL\_LINE\_VS\_IMMATUE\_T\_CELL\_LINE\_UP |  | 20 | 0.23 | 0.96 | 0.516 | 1.000 | 1.000 | 568 | tags=55%, list=40%, signal=91% |
| 559 | GSE13485\_CTRL\_VS\_DAY7\_YF17D\_VACCINE\_PBMC\_DN |  | 26 | 0.21 | 0.96 | 0.494 | 1.000 | 1.000 | 263 | tags=27%, list=19%, signal=32% |
| 560 | GSE2770\_IL12\_VS\_TGFB\_AND\_IL12\_TREATED\_ACT\_CD4\_TCELL\_48H\_DN |  | 23 | 0.21 | 0.96 | 0.496 | 1.000 | 1.000 | 264 | tags=30%, list=19%, signal=37% |
| 561 | GSE13493\_CD4INTCD8POS\_VS\_CD8POS\_THYMOCYTE\_DN |  | 16 | 0.24 | 0.96 | 0.509 | 1.000 | 1.000 | 317 | tags=38%, list=22%, signal=48% |
| 562 | GSE11864\_CSF1\_IFNG\_VS\_CSF1\_IFNG\_PAM3CYS\_IN\_MAC\_UP |  | 18 | 0.24 | 0.96 | 0.508 | 1.000 | 1.000 | 499 | tags=50%, list=35%, signal=76% |
| 563 | GSE45382\_UNTREATED\_VS\_TGFB\_TREATED\_MACROPHAGES\_UP |  | 22 | 0.22 | 0.96 | 0.486 | 1.000 | 1.000 | 623 | tags=64%, list=44%, signal=112% |
| 564 | GSE14350\_IL2RB\_KO\_VS\_WT\_TEFF\_DN |  | 19 | 0.23 | 0.96 | 0.526 | 1.000 | 1.000 | 195 | tags=26%, list=14%, signal=30% |
| 565 | GSE17721\_LPS\_VS\_GARDIQUIMOD\_8H\_BMDC\_DN |  | 17 | 0.24 | 0.96 | 0.507 | 1.000 | 1.000 | 62 | tags=18%, list=4%, signal=18% |
| 566 | GSE2770\_IL12\_VS\_IL4\_TREATED\_ACT\_CD4\_TCELL\_48H\_DN |  | 21 | 0.22 | 0.96 | 0.505 | 1.000 | 1.000 | 217 | tags=29%, list=15%, signal=33% |
| 567 | GSE27786\_LSK\_VS\_BCELL\_UP |  | 21 | 0.22 | 0.95 | 0.503 | 1.000 | 1.000 | 575 | tags=57%, list=41%, signal=95% |
| 568 | GSE1460\_CD4\_THYMOCYTE\_VS\_NAIVE\_CD4\_TCELL\_CORD\_BLOOD\_DN |  | 16 | 0.24 | 0.95 | 0.493 | 1.000 | 1.000 | 407 | tags=44%, list=29%, signal=61% |
| 569 | GSE37301\_MULTIPOTENT\_PROGENITOR\_VS\_LYMPHOID\_PRIMED\_MPP\_UP |  | 24 | 0.21 | 0.95 | 0.515 | 1.000 | 1.000 | 689 | tags=71%, list=49%, signal=136% |
| 570 | GSE3982\_DC\_VS\_NEUTROPHIL\_UP |  | 15 | 0.25 | 0.95 | 0.489 | 1.000 | 1.000 | 264 | tags=33%, list=19%, signal=41% |
| 571 | GSE46606\_IRF4MID\_VS\_WT\_CD40L\_IL2\_IL5\_DAY3\_STIMULATED\_BCELL\_UP |  | 29 | 0.20 | 0.95 | 0.530 | 1.000 | 1.000 | 93 | tags=17%, list=7%, signal=18% |
| 572 | GSE10240\_CTRL\_VS\_IL22\_STIM\_PRIMARY\_BRONCHIAL\_EPITHELIAL\_CELLS\_DN |  | 15 | 0.25 | 0.95 | 0.499 | 1.000 | 1.000 | 616 | tags=60%, list=44%, signal=105% |
| 573 | GSE2770\_TGFB\_AND\_IL4\_VS\_TGFB\_AND\_IL12\_TREATED\_ACT\_CD4\_TCELL\_2H\_DN |  | 30 | 0.20 | 0.95 | 0.532 | 1.000 | 1.000 | 225 | tags=23%, list=16%, signal=27% |
| 574 | GSE13547\_2H\_VS\_12\_H\_ANTI\_IGM\_STIM\_ZFX\_KO\_BCELL\_DN |  | 17 | 0.23 | 0.95 | 0.512 | 1.000 | 1.000 | 365 | tags=35%, list=26%, signal=47% |
| 575 | GSE17721\_LPS\_VS\_GARDIQUIMOD\_0.5H\_BMDC\_UP |  | 15 | 0.25 | 0.95 | 0.489 | 1.000 | 1.000 | 286 | tags=33%, list=20%, signal=41% |
| 576 | GSE11961\_PLASMA\_CELL\_DAY7\_VS\_GERMINAL\_CENTER\_BCELL\_DAY40\_UP |  | 15 | 0.25 | 0.95 | 0.505 | 1.000 | 1.000 | 132 | tags=20%, list=9%, signal=22% |
| 577 | GSE11961\_GERMINAL\_CENTER\_BCELL\_DAY7\_VS\_MEMORY\_BCELL\_DAY40\_UP |  | 21 | 0.22 | 0.95 | 0.510 | 1.000 | 1.000 | 324 | tags=38%, list=23%, signal=49% |
| 578 | GSE5503\_LIVER\_DC\_VS\_PLN\_DC\_ACTIVATED\_ALLOGENIC\_TCELL\_UP |  | 15 | 0.25 | 0.95 | 0.521 | 1.000 | 1.000 | 25 | tags=13%, list=2%, signal=13% |
| 579 | GSE46606\_UNSTIM\_VS\_CD40L\_IL2\_IL5\_3DAY\_STIMULATED\_IRF4HIGH\_SORTED\_BCELL\_UP |  | 21 | 0.22 | 0.95 | 0.521 | 1.000 | 1.000 | 375 | tags=43%, list=27%, signal=58% |
| 580 | GSE3920\_UNTREATED\_VS\_IFNG\_TREATED\_ENDOTHELIAL\_CELL\_DN |  | 16 | 0.24 | 0.95 | 0.533 | 1.000 | 1.000 | 669 | tags=69%, list=47%, signal=129% |
| 581 | GSE37532\_VISCERAL\_ADIPOSE\_TISSUE\_VS\_LN\_DERIVED\_PPARG\_KO\_TREG\_CD4\_TCELL\_UP |  | 23 | 0.21 | 0.95 | 0.514 | 1.000 | 1.000 | 588 | tags=57%, list=42%, signal=95% |
| 582 | GSE40274\_LEF1\_VS\_FOXP3\_AND\_LEF1\_TRANSDUCED\_ACTIVATED\_CD4\_TCELL\_UP |  | 20 | 0.23 | 0.95 | 0.521 | 1.000 | 1.000 | 198 | tags=25%, list=14%, signal=29% |
| 583 | GSE5589\_WT\_VS\_IL10\_KO\_LPS\_AND\_IL10\_STIM\_MACROPHAGE\_180MIN\_UP |  | 20 | 0.23 | 0.95 | 0.518 | 1.000 | 1.000 | 635 | tags=60%, list=45%, signal=108% |
| 584 | GSE30971\_2H\_VS\_4H\_LPS\_STIM\_MACROPHAGE\_WBP7\_HET\_UP |  | 20 | 0.22 | 0.95 | 0.511 | 1.000 | 1.000 | 638 | tags=60%, list=45%, signal=108% |
| 585 | GSE42088\_UNINF\_VS\_LEISHMANIA\_INF\_DC\_8H\_UP |  | 31 | 0.19 | 0.95 | 0.525 | 1.000 | 1.000 | 562 | tags=52%, list=40%, signal=84% |
| 586 | GSE24142\_ADULT\_VS\_FETAL\_DN3\_THYMOCYTE\_UP |  | 27 | 0.20 | 0.95 | 0.520 | 1.000 | 1.000 | 300 | tags=33%, list=21%, signal=42% |
| 587 | GSE22589\_HEALTHY\_VS\_HIV\_INFECTED\_DC\_UP |  | 24 | 0.21 | 0.95 | 0.519 | 1.000 | 1.000 | 849 | tags=83%, list=60%, signal=206% |
| 588 | GSE42724\_NAIVE\_VS\_B1\_BCELL\_UP |  | 22 | 0.22 | 0.94 | 0.541 | 1.000 | 1.000 | 258 | tags=32%, list=18%, signal=38% |
| 589 | GSE22611\_UNSTIM\_VS\_2H\_MDP\_STIM\_MUTANT\_NOD2\_TRANSDUCED\_HEK293T\_CELL\_UP |  | 19 | 0.23 | 0.94 | 0.521 | 1.000 | 1.000 | 1094 | tags=100%, list=78%, signal=439% |
| 590 | GSE15330\_HSC\_VS\_MEGAKARYOCYTE\_ERYTHROID\_PROGENITOR\_DN |  | 23 | 0.20 | 0.94 | 0.540 | 1.000 | 1.000 | 217 | tags=26%, list=15%, signal=30% |
| 591 | GSE26023\_PHD3\_KO\_VS\_WT\_NEUTROPHIL\_HYPOXIA\_DN |  | 16 | 0.24 | 0.94 | 0.518 | 1.000 | 1.000 | 78 | tags=19%, list=6%, signal=20% |
| 592 | GSE1740\_UNSTIM\_VS\_IFNA\_STIMULATED\_MCSF\_DERIVED\_MACROPHAGE\_DN |  | 17 | 0.24 | 0.94 | 0.519 | 1.000 | 1.000 | 69 | tags=18%, list=5%, signal=18% |
| 593 | GSE5099\_UNSTIM\_VS\_MCSF\_TREATED\_MONOCYTE\_DAY3\_UP |  | 23 | 0.21 | 0.94 | 0.547 | 1.000 | 1.000 | 641 | tags=61%, list=45%, signal=110% |
| 594 | GSE40666\_UNTREATED\_VS\_IFNA\_STIM\_EFFECTOR\_CD8\_TCELL\_90MIN\_DN |  | 20 | 0.22 | 0.94 | 0.538 | 1.000 | 1.000 | 202 | tags=25%, list=14%, signal=29% |
| 595 | GSE17721\_CPG\_VS\_GARDIQUIMOD\_1H\_BMDC\_UP |  | 17 | 0.23 | 0.94 | 0.545 | 1.000 | 1.000 | 227 | tags=29%, list=16%, signal=35% |
| 596 | GSE13485\_DAY1\_VS\_DAY7\_YF17D\_VACCINE\_PBMC\_DN |  | 27 | 0.20 | 0.94 | 0.526 | 1.000 | 1.000 | 289 | tags=30%, list=20%, signal=37% |
| 597 | GSE15659\_CD45RA\_NEG\_CD4\_TCELL\_VS\_NONSUPPRESSIVE\_TCELL\_DN |  | 15 | 0.25 | 0.94 | 0.540 | 1.000 | 1.000 | 263 | tags=33%, list=19%, signal=41% |
| 598 | GSE26488\_HDAC7\_KO\_VS\_VP16\_TRANSGENIC\_HDAC7\_KO\_DOUBLE\_POSITIVE\_THYMOCYTE\_UP |  | 22 | 0.21 | 0.94 | 0.533 | 1.000 | 1.000 | 214 | tags=27%, list=15%, signal=32% |
| 599 | GSE31082\_DN\_VS\_CD8\_SP\_THYMOCYTE\_DN |  | 16 | 0.23 | 0.94 | 0.540 | 1.000 | 1.000 | 387 | tags=50%, list=27%, signal=68% |
| 600 | GSE14415\_TCONV\_VS\_FOXP3\_KO\_INDUCED\_TREG\_UP |  | 29 | 0.19 | 0.94 | 0.534 | 1.000 | 1.000 | 498 | tags=48%, list=35%, signal=73% |
| 601 | GSE10422\_WT\_VS\_BAFF\_TRANSGENIC\_LN\_BCELL\_UP |  | 20 | 0.23 | 0.94 | 0.522 | 1.000 | 1.000 | 139 | tags=20%, list=10%, signal=22% |
| 602 | GSE8835\_HEALTHY\_VS\_CLL\_CD8\_TCELL\_DN |  | 17 | 0.23 | 0.94 | 0.531 | 1.000 | 1.000 | 93 | tags=18%, list=7%, signal=19% |
| 603 | GSE24634\_TEFF\_VS\_TCONV\_DAY3\_IN\_CULTURE\_UP |  | 21 | 0.21 | 0.94 | 0.527 | 1.000 | 1.000 | 870 | tags=86%, list=62%, signal=220% |
| 604 | GSE21546\_WT\_VS\_SAP1A\_KO\_AND\_ELK1\_KO\_DP\_THYMOCYTES\_UP |  | 23 | 0.21 | 0.94 | 0.541 | 1.000 | 1.000 | 732 | tags=78%, list=52%, signal=160% |
| 605 | GSE14415\_FOXP3\_KO\_NATURAL\_TREG\_VS\_TCONV\_DN |  | 23 | 0.22 | 0.93 | 0.570 | 1.000 | 1.000 | 344 | tags=39%, list=24%, signal=51% |
| 606 | GSE23321\_EFFECTOR\_MEMORY\_VS\_NAIVE\_CD8\_TCELL\_DN |  | 28 | 0.20 | 0.93 | 0.533 | 1.000 | 1.000 | 593 | tags=54%, list=42%, signal=91% |
| 607 | GSE13484\_12H\_VS\_3H\_YF17D\_VACCINE\_STIM\_PBMC\_DN |  | 28 | 0.20 | 0.93 | 0.560 | 1.000 | 1.000 | 27 | tags=11%, list=2%, signal=11% |
| 608 | GSE17721\_POLYIC\_VS\_CPG\_6H\_BMDC\_DN |  | 22 | 0.22 | 0.93 | 0.542 | 1.000 | 1.000 | 272 | tags=32%, list=19%, signal=39% |
| 609 | GSE40274\_CTRL\_VS\_FOXP3\_AND\_PBX1\_TRANSDUCED\_ACTIVATED\_CD4\_TCELL\_UP |  | 17 | 0.24 | 0.93 | 0.513 | 1.000 | 1.000 | 12 | tags=12%, list=1%, signal=12% |
| 610 | GSE2585\_CTEC\_VS\_THYMIC\_MACROPHAGE\_DN |  | 20 | 0.22 | 0.93 | 0.521 | 1.000 | 1.000 | 502 | tags=50%, list=36%, signal=77% |
| 611 | GSE42021\_TCONV\_PLN\_VS\_TREG\_PRECURSORS\_THYMUS\_UP |  | 26 | 0.20 | 0.93 | 0.551 | 1.000 | 1.000 | 27 | tags=12%, list=2%, signal=12% |
| 612 | GSE26351\_UNSTIM\_VS\_WNT\_PATHWAY\_STIM\_HEMATOPOIETIC\_PROGENITORS\_UP |  | 24 | 0.21 | 0.93 | 0.561 | 1.000 | 1.000 | 492 | tags=46%, list=35%, signal=69% |
| 613 | GSE369\_SOCS3\_KO\_VS\_WT\_LIVER\_UP |  | 25 | 0.20 | 0.93 | 0.551 | 1.000 | 1.000 | 1022 | tags=96%, list=72%, signal=342% |
| 614 | GSE27859\_DC\_VS\_CD11C\_INT\_F480\_HI\_MACROPHAGE\_DN |  | 20 | 0.22 | 0.93 | 0.538 | 1.000 | 1.000 | 532 | tags=55%, list=38%, signal=87% |
| 615 | GSE2770\_TGFB\_AND\_IL4\_VS\_TGFB\_AND\_IL12\_TREATED\_ACT\_CD4\_TCELL\_2H\_UP |  | 15 | 0.24 | 0.93 | 0.538 | 1.000 | 1.000 | 30 | tags=13%, list=2%, signal=13% |
| 616 | GSE13493\_DP\_VS\_CD4INTCD8POS\_THYMOCYTE\_DN |  | 17 | 0.23 | 0.93 | 0.539 | 1.000 | 1.000 | 753 | tags=71%, list=53%, signal=150% |
| 617 | GSE3400\_UNTREATED\_VS\_IFNB\_TREATED\_MEF\_UP |  | 20 | 0.22 | 0.93 | 0.535 | 1.000 | 1.000 | 90 | tags=15%, list=6%, signal=16% |
| 618 | GSE22886\_CD8\_TCELL\_VS\_BCELL\_NAIVE\_DN |  | 15 | 0.24 | 0.93 | 0.540 | 1.000 | 1.000 | 413 | tags=47%, list=29%, signal=65% |
| 619 | GSE7568\_IL4\_VS\_IL4\_AND\_TGFB\_TREATED\_MACROPHAGE\_24H\_UP |  | 22 | 0.21 | 0.92 | 0.558 | 1.000 | 1.000 | 159 | tags=18%, list=11%, signal=20% |
| 620 | GSE13411\_SWITCHED\_MEMORY\_BCELL\_VS\_PLASMA\_CELL\_UP |  | 32 | 0.19 | 0.92 | 0.553 | 1.000 | 1.000 | 264 | tags=28%, list=19%, signal=34% |
| 621 | GSE7568\_IL4\_TGFB\_DEXAMETHASONE\_VS\_IL4\_TGFB\_TREATED\_MACROPHAGE\_UP |  | 22 | 0.21 | 0.92 | 0.563 | 1.000 | 1.000 | 900 | tags=86%, list=64%, signal=235% |
| 622 | GSE27291\_0H\_VS\_6H\_STIM\_GAMMADELTA\_TCELL\_DN |  | 16 | 0.24 | 0.92 | 0.553 | 1.000 | 1.000 | 878 | tags=88%, list=62%, signal=229% |
| 623 | GSE360\_L\_DONOVANI\_VS\_B\_MALAYI\_LOW\_DOSE\_MAC\_UP |  | 22 | 0.21 | 0.92 | 0.554 | 1.000 | 1.000 | 57 | tags=14%, list=4%, signal=14% |
| 624 | GSE29618\_PRE\_VS\_DAY7\_POST\_TIV\_FLU\_VACCINE\_MONOCYTE\_DN |  | 15 | 0.24 | 0.92 | 0.558 | 1.000 | 1.000 | 101 | tags=20%, list=7%, signal=21% |
| 625 | GSE27786\_CD8\_TCELL\_VS\_NEUTROPHIL\_UP |  | 26 | 0.20 | 0.92 | 0.545 | 1.000 | 1.000 | 734 | tags=65%, list=52%, signal=134% |
| 626 | GSE16385\_IFNG\_TNF\_VS\_UNSTIM\_MACROPHAGE\_ROSIGLITAZONE\_TREATED\_UP |  | 17 | 0.24 | 0.92 | 0.527 | 1.000 | 1.000 | 493 | tags=53%, list=35%, signal=80% |
| 627 | GSE22886\_IL2\_VS\_IL15\_STIM\_NKCELL\_DN |  | 19 | 0.22 | 0.92 | 0.556 | 1.000 | 1.000 | 162 | tags=21%, list=11%, signal=23% |
| 628 | GSE5589\_UNSTIM\_VS\_45MIN\_LPS\_AND\_IL10\_STIM\_MACROPHAGE\_DN |  | 17 | 0.23 | 0.92 | 0.544 | 1.000 | 1.000 | 318 | tags=35%, list=23%, signal=45% |
| 629 | GSE19888\_ADENOSINE\_A3R\_ACT\_VS\_TCELL\_MEMBRANES\_ACT\_IN\_MAST\_CELL\_DN |  | 21 | 0.22 | 0.92 | 0.576 | 1.000 | 1.000 | 329 | tags=38%, list=23%, signal=49% |
| 630 | GSE41867\_NAIVE\_VS\_DAY6\_LCMV\_ARMSTRONG\_EFFECTOR\_CD8\_TCELL\_UP |  | 16 | 0.24 | 0.92 | 0.570 | 1.000 | 1.000 | 375 | tags=38%, list=27%, signal=50% |
| 631 | GSE45365\_HEALTHY\_VS\_MCMV\_INFECTION\_CD8A\_DC\_DN |  | 25 | 0.20 | 0.92 | 0.555 | 1.000 | 1.000 | 83 | tags=16%, list=6%, signal=17% |
| 632 | GSE17186\_BLOOD\_VS\_CORD\_BLOOD\_CD21LOW\_TRANSITIONAL\_BCELL\_DN |  | 21 | 0.22 | 0.92 | 0.584 | 1.000 | 1.000 | 307 | tags=33%, list=22%, signal=42% |
| 633 | GSE9960\_HEALTHY\_VS\_GRAM\_NEG\_AND\_POS\_SEPSIS\_PBMC\_DN |  | 22 | 0.21 | 0.92 | 0.549 | 1.000 | 1.000 | 345 | tags=36%, list=24%, signal=47% |
| 634 | GSE360\_L\_MAJOR\_VS\_B\_MALAYI\_HIGH\_DOSE\_MAC\_UP |  | 19 | 0.22 | 0.92 | 0.577 | 1.000 | 1.000 | 405 | tags=37%, list=29%, signal=51% |
| 635 | GSE13485\_PRE\_VS\_POST\_YF17D\_VACCINATION\_PBMC\_DN |  | 17 | 0.23 | 0.92 | 0.553 | 1.000 | 1.000 | 356 | tags=41%, list=25%, signal=54% |
| 636 | GSE32423\_IL7\_VS\_IL7\_IL4\_MEMORY\_CD8\_TCELL\_DN |  | 15 | 0.24 | 0.92 | 0.566 | 1.000 | 1.000 | 961 | tags=93%, list=68%, signal=290% |
| 637 | GSE11057\_NAIVE\_CD4\_VS\_PBMC\_CD4\_TCELL\_UP |  | 24 | 0.20 | 0.91 | 0.569 | 1.000 | 1.000 | 140 | tags=21%, list=10%, signal=23% |
| 638 | GSE27786\_LIN\_NEG\_VS\_CD4\_TCELL\_UP |  | 20 | 0.22 | 0.91 | 0.562 | 1.000 | 1.000 | 780 | tags=75%, list=55%, signal=165% |
| 639 | GSE34515\_CD16\_NEG\_VS\_POS\_MONOCYTE\_DN |  | 15 | 0.23 | 0.91 | 0.568 | 1.000 | 1.000 | 87 | tags=20%, list=6%, signal=21% |
| 640 | GSE17721\_POLYIC\_VS\_PAM3CSK4\_6H\_BMDC\_DN |  | 30 | 0.19 | 0.91 | 0.572 | 1.000 | 1.000 | 399 | tags=37%, list=28%, signal=50% |
| 641 | GSE41867\_DAY6\_EFFECTOR\_VS\_DAY30\_EXHAUSTED\_CD8\_TCELL\_LCMV\_CLONE13\_DN |  | 28 | 0.19 | 0.91 | 0.566 | 1.000 | 1.000 | 224 | tags=25%, list=16%, signal=29% |
| 642 | GSE12198\_LOW\_IL2\_STIM\_NK\_CELL\_VS\_HIGH\_IL2\_STIM\_NK\_CELL\_DN |  | 15 | 0.24 | 0.91 | 0.573 | 1.000 | 1.000 | 489 | tags=53%, list=35%, signal=81% |
| 643 | GSE29164\_DAY3\_VS\_DAY7\_UNTREATED\_MELANOMA\_UP |  | 21 | 0.21 | 0.91 | 0.551 | 1.000 | 1.000 | 203 | tags=24%, list=14%, signal=27% |
| 644 | GSE16266\_LPS\_VS\_HEATSHOCK\_AND\_LPS\_STIM\_MEF\_DN |  | 19 | 0.22 | 0.91 | 0.551 | 1.000 | 1.000 | 79 | tags=16%, list=6%, signal=17% |
| 645 | GSE26030\_UNSTIM\_VS\_RESTIM\_TH17\_DAY5\_POST\_POLARIZATION\_UP |  | 17 | 0.23 | 0.91 | 0.551 | 1.000 | 1.000 | 392 | tags=41%, list=28%, signal=56% |
| 646 | GSE22886\_NEUTROPHIL\_VS\_DC\_DN |  | 22 | 0.20 | 0.91 | 0.567 | 1.000 | 1.000 | 616 | tags=55%, list=44%, signal=95% |
| 647 | GSE19888\_CTRL\_VS\_A3R\_ACT\_TREATED\_MAST\_CELL\_PRETREATED\_WITH\_A3R\_INH\_DN |  | 17 | 0.22 | 0.91 | 0.575 | 1.000 | 1.000 | 318 | tags=35%, list=23%, signal=45% |
| 648 | GSE25088\_ROSIGLITAZONE\_VS\_IL4\_AND\_ROSIGLITAZONE\_STIM\_MACROPHAGE\_DAY10\_UP |  | 17 | 0.23 | 0.91 | 0.583 | 1.000 | 1.000 | 193 | tags=29%, list=14%, signal=34% |
| 649 | GSE17721\_POLYIC\_VS\_PAM3CSK4\_0.5H\_BMDC\_UP |  | 16 | 0.24 | 0.91 | 0.574 | 1.000 | 1.000 | 88 | tags=19%, list=6%, signal=20% |
| 650 | GSE17974\_0H\_VS\_4H\_IN\_VITRO\_ACT\_CD4\_TCELL\_DN |  | 28 | 0.19 | 0.91 | 0.561 | 1.000 | 1.000 | 773 | tags=75%, list=55%, signal=163% |
| 651 | GSE15330\_HSC\_VS\_LYMPHOID\_PRIMED\_MULTIPOTENT\_PROGENITOR\_IKAROS\_KO\_UP |  | 16 | 0.23 | 0.91 | 0.554 | 1.000 | 1.000 | 680 | tags=69%, list=48%, signal=131% |
| 652 | GSE22886\_NAIVE\_CD4\_TCELL\_VS\_DC\_DN |  | 16 | 0.23 | 0.91 | 0.545 | 1.000 | 1.000 | 439 | tags=50%, list=31%, signal=72% |
| 653 | GSE21360\_PRIMARY\_VS\_QUATERNARY\_MEMORY\_CD8\_TCELL\_DN |  | 31 | 0.18 | 0.91 | 0.595 | 1.000 | 1.000 | 164 | tags=19%, list=12%, signal=21% |
| 654 | GSE23505\_IL6\_IL1\_IL23\_VS\_IL6\_IL1\_TGFB\_TREATED\_CD4\_TCELL\_DN |  | 15 | 0.24 | 0.91 | 0.558 | 1.000 | 1.000 | 144 | tags=20%, list=10%, signal=22% |
| 655 | GSE22025\_TGFB1\_VS\_TGFB1\_AND\_PROGESTERONE\_TREATED\_CD4\_TCELL\_DN |  | 15 | 0.24 | 0.90 | 0.588 | 1.000 | 1.000 | 119 | tags=20%, list=8%, signal=22% |
| 656 | GSE5099\_MONOCYTE\_VS\_ALTERNATIVE\_M2\_MACROPHAGE\_UP |  | 17 | 0.23 | 0.90 | 0.548 | 1.000 | 1.000 | 253 | tags=29%, list=18%, signal=35% |
| 657 | GSE39556\_CD8A\_DC\_VS\_NK\_CELL\_DN |  | 24 | 0.20 | 0.90 | 0.585 | 1.000 | 1.000 | 518 | tags=54%, list=37%, signal=84% |
| 658 | GSE3982\_NEUTROPHIL\_VS\_TH2\_DN |  | 17 | 0.22 | 0.90 | 0.570 | 1.000 | 1.000 | 683 | tags=65%, list=48%, signal=124% |
| 659 | GSE39556\_CD8A\_DC\_VS\_NK\_CELL\_MOUSE\_3H\_POST\_POLYIC\_INJ\_DN |  | 22 | 0.20 | 0.90 | 0.572 | 1.000 | 1.000 | 161 | tags=23%, list=11%, signal=25% |
| 660 | GSE2770\_IL12\_VS\_TGFB\_AND\_IL12\_TREATED\_ACT\_CD4\_TCELL\_6H\_DN |  | 18 | 0.22 | 0.90 | 0.590 | 1.000 | 1.000 | 602 | tags=61%, list=43%, signal=105% |
| 661 | GSE17721\_0.5H\_VS\_4H\_GARDIQUIMOD\_BMDC\_UP |  | 19 | 0.22 | 0.90 | 0.574 | 1.000 | 1.000 | 215 | tags=26%, list=15%, signal=31% |
| 662 | GSE22611\_MUTANT\_NOD2\_TRANSDUCED\_VS\_CTRL\_HEK293T\_STIMULATED\_WITH\_MDP\_6H\_UP |  | 16 | 0.23 | 0.90 | 0.579 | 1.000 | 1.000 | 78 | tags=19%, list=6%, signal=20% |
| 663 | GSE16385\_MONOCYTE\_VS\_12H\_IL4\_TREATED\_MACROPHAGE\_UP |  | 17 | 0.22 | 0.90 | 0.600 | 1.000 | 1.000 | 163 | tags=24%, list=12%, signal=26% |
| 664 | GSE21380\_NON\_TFH\_VS\_TFH\_CD4\_TCELL\_DN |  | 19 | 0.22 | 0.90 | 0.590 | 1.000 | 1.000 | 324 | tags=37%, list=23%, signal=47% |
| 665 | GSE22611\_MUTANT\_NOD2\_VS\_CTRL\_TRANSDUCED\_HEK293T\_CELL\_DN |  | 15 | 0.23 | 0.90 | 0.591 | 1.000 | 1.000 | 328 | tags=40%, list=23%, signal=52% |
| 666 | GSE8515\_CTRL\_VS\_IL1\_4H\_STIM\_MAC\_DN |  | 20 | 0.22 | 0.90 | 0.597 | 1.000 | 1.000 | 478 | tags=50%, list=34%, signal=75% |
| 667 | GSE16450\_CTRL\_VS\_IFNA\_6H\_STIM\_MATURE\_NEURON\_CELL\_LINE\_DN |  | 15 | 0.24 | 0.90 | 0.562 | 1.000 | 1.000 | 873 | tags=87%, list=62%, signal=225% |
| 668 | GSE6259\_BCELL\_VS\_CD4\_TCELL\_UP |  | 19 | 0.21 | 0.90 | 0.588 | 1.000 | 1.000 | 127 | tags=16%, list=9%, signal=17% |
| 669 | GSE11864\_UNTREATED\_VS\_CSF1\_IFNG\_IN\_MAC\_DN |  | 26 | 0.19 | 0.90 | 0.599 | 1.000 | 1.000 | 512 | tags=50%, list=36%, signal=77% |
| 670 | GSE17721\_LPS\_VS\_CPG\_0.5H\_BMDC\_UP |  | 17 | 0.23 | 0.90 | 0.593 | 1.000 | 1.000 | 158 | tags=24%, list=11%, signal=26% |
| 671 | GSE32423\_IL7\_VS\_IL7\_IL4\_NAIVE\_CD8\_TCELL\_DN |  | 25 | 0.19 | 0.89 | 0.596 | 1.000 | 1.000 | 35 | tags=12%, list=2%, signal=12% |
| 672 | GSE12366\_PLASMA\_CELL\_VS\_MEMORY\_BCELL\_DN |  | 17 | 0.22 | 0.89 | 0.594 | 1.000 | 1.000 | 1000 | tags=94%, list=71%, signal=319% |
| 673 | GSE3982\_NEUTROPHIL\_VS\_BCELL\_DN |  | 20 | 0.21 | 0.89 | 0.587 | 1.000 | 1.000 | 320 | tags=30%, list=23%, signal=38% |
| 674 | GSE12198\_NK\_VS\_NK\_ACT\_EXPANSION\_SYSTEM\_DERIVED\_NK\_CELL\_UP |  | 20 | 0.22 | 0.89 | 0.588 | 1.000 | 1.000 | 84 | tags=15%, list=6%, signal=16% |
| 675 | GSE17974\_2.5H\_VS\_72H\_IL4\_AND\_ANTI\_IL12\_ACT\_CD4\_TCELL\_UP |  | 26 | 0.19 | 0.89 | 0.591 | 1.000 | 1.000 | 334 | tags=35%, list=24%, signal=45% |
| 676 | GSE46468\_LUNG\_INNATE\_LYMPHOID\_CELL\_VS\_SPLEEN\_CD4\_TCELL\_UP |  | 25 | 0.19 | 0.89 | 0.573 | 1.000 | 1.000 | 408 | tags=40%, list=29%, signal=55% |
| 677 | GSE45365\_CD8A\_DC\_VS\_CD11B\_DC\_DN |  | 16 | 0.23 | 0.89 | 0.590 | 1.000 | 1.000 | 412 | tags=44%, list=29%, signal=61% |
| 678 | GSE30962\_PRIMARY\_VS\_SECONDARY\_CHRONIC\_LCMV\_INF\_CD8\_TCELL\_UP |  | 19 | 0.21 | 0.89 | 0.598 | 1.000 | 1.000 | 824 | tags=79%, list=58%, signal=187% |
| 679 | GSE7509\_DC\_VS\_MONOCYTE\_DN |  | 20 | 0.20 | 0.89 | 0.609 | 1.000 | 1.000 | 124 | tags=20%, list=9%, signal=22% |
| 680 | GSE25123\_WT\_VS\_PPARG\_KO\_MACROPHAGE\_IL4\_AND\_ROSIGLITAZONE\_STIM\_DN |  | 17 | 0.22 | 0.89 | 0.587 | 1.000 | 1.000 | 73 | tags=18%, list=5%, signal=18% |
| 681 | GSE28237\_FOLLICULAR\_VS\_EARLY\_GC\_BCELL\_UP |  | 24 | 0.20 | 0.89 | 0.588 | 1.000 | 1.000 | 787 | tags=75%, list=56%, signal=167% |
| 682 | GSE45365\_BCELL\_VS\_CD8\_TCELL\_DN |  | 15 | 0.24 | 0.89 | 0.587 | 1.000 | 1.000 | 98 | tags=20%, list=7%, signal=21% |
| 683 | GSE22935\_UNSTIM\_VS\_48H\_MBOVIS\_BCG\_STIM\_MYD88\_KO\_MACROPHAGE\_UP |  | 18 | 0.22 | 0.89 | 0.597 | 1.000 | 1.000 | 326 | tags=39%, list=23%, signal=50% |
| 684 | GSE36888\_UNTREATED\_VS\_IL2\_TREATED\_STAT5\_AB\_KNOCKIN\_TCELL\_6H\_DN |  | 20 | 0.21 | 0.89 | 0.604 | 1.000 | 1.000 | 409 | tags=40%, list=29%, signal=56% |
| 685 | GSE29164\_CD8\_TCELL\_VS\_CD8\_TCELL\_AND\_IL12\_TREATED\_MELANOMA\_DAY7\_UP |  | 17 | 0.22 | 0.89 | 0.625 | 1.000 | 1.000 | 578 | tags=59%, list=41%, signal=98% |
| 686 | GSE20198\_UNTREATED\_VS\_IL12\_TREATED\_ACT\_CD4\_TCELL\_UP |  | 24 | 0.20 | 0.89 | 0.588 | 1.000 | 1.000 | 193 | tags=21%, list=14%, signal=24% |
| 687 | GSE7852\_TREG\_VS\_TCONV\_FAT\_DN |  | 20 | 0.21 | 0.89 | 0.594 | 1.000 | 1.000 | 219 | tags=30%, list=16%, signal=35% |
| 688 | GSE42021\_TCONV\_PLN\_VS\_CD24INT\_TCONV\_THYMUS\_DN |  | 21 | 0.21 | 0.89 | 0.624 | 1.000 | 1.000 | 24 | tags=10%, list=2%, signal=10% |
| 689 | GSE41978\_WT\_VS\_ID2\_KO\_AND\_BIM\_KO\_KLRG1\_LOW\_EFFECTOR\_CD8\_TCELL\_UP |  | 15 | 0.24 | 0.89 | 0.596 | 1.000 | 1.000 | 70 | tags=20%, list=5%, signal=21% |
| 690 | GSE40274\_CTRL\_VS\_FOXP3\_TRANSDUCED\_ACTIVATED\_CD4\_TCELL\_DN |  | 26 | 0.19 | 0.89 | 0.599 | 1.000 | 1.000 | 79 | tags=15%, list=6%, signal=16% |
| 691 | GSE43863\_TH1\_VS\_LY6C\_INT\_CXCR5POS\_MEMORY\_CD4\_TCELL\_UP |  | 31 | 0.18 | 0.89 | 0.584 | 1.000 | 1.000 | 865 | tags=84%, list=61%, signal=212% |
| 692 | GSE42724\_B1\_BCELL\_VS\_PLASMABLAST\_UP |  | 16 | 0.23 | 0.89 | 0.590 | 1.000 | 1.000 | 76 | tags=19%, list=5%, signal=20% |
| 693 | GSE15330\_WT\_VS\_IKAROS\_KO\_LYMPHOID\_MULTIPOTENT\_PROGENITOR\_DN |  | 26 | 0.19 | 0.89 | 0.604 | 1.000 | 1.000 | 958 | tags=88%, list=68%, signal=270% |
| 694 | GSE15330\_WT\_VS\_IKAROS\_KO\_LYMPHOID\_MULTIPOTENT\_PROGENITOR\_UP |  | 16 | 0.22 | 0.89 | 0.585 | 1.000 | 1.000 | 300 | tags=31%, list=21%, signal=39% |
| 695 | GSE9960\_GRAM\_NEG\_VS\_GRAM\_NEG\_AND\_POS\_SEPSIS\_PBMC\_UP |  | 16 | 0.22 | 0.88 | 0.583 | 1.000 | 1.000 | 1097 | tags=100%, list=78%, signal=444% |
| 696 | GSE42021\_TREG\_PLN\_VS\_TREG\_PRECURSORS\_THYMUS\_UP |  | 20 | 0.20 | 0.88 | 0.613 | 1.000 | 1.000 | 298 | tags=30%, list=21%, signal=37% |
| 697 | GSE28737\_WT\_VS\_BCL6\_KO\_MARGINAL\_ZONE\_BCELL\_DN |  | 17 | 0.22 | 0.88 | 0.598 | 1.000 | 1.000 | 482 | tags=53%, list=34%, signal=79% |
| 698 | GSE43955\_10H\_VS\_30H\_ACT\_CD4\_TCELL\_UP |  | 17 | 0.22 | 0.88 | 0.620 | 1.000 | 1.000 | 207 | tags=24%, list=15%, signal=27% |
| 699 | GSE2770\_IL12\_AND\_TGFB\_ACT\_VS\_ACT\_CD4\_TCELL\_6H\_DN |  | 29 | 0.18 | 0.88 | 0.611 | 1.000 | 1.000 | 133 | tags=17%, list=9%, signal=19% |
| 700 | GSE15659\_CD45RA\_NEG\_CD4\_TCELL\_VS\_NONSUPPRESSIVE\_TCELL\_UP |  | 16 | 0.22 | 0.88 | 0.603 | 1.000 | 1.000 | 513 | tags=56%, list=36%, signal=87% |
| 701 | GSE37533\_PPARG2\_FOXP3\_VS\_FOXP3\_TRANSDUCED\_CD4\_TCELL\_UP |  | 33 | 0.18 | 0.88 | 0.610 | 1.000 | 1.000 | 835 | tags=76%, list=59%, signal=181% |
| 702 | GSE12845\_IGD\_POS\_BLOOD\_VS\_DARKZONE\_GC\_TONSIL\_BCELL\_DN |  | 20 | 0.21 | 0.88 | 0.608 | 1.000 | 1.000 | 14 | tags=10%, list=1%, signal=10% |
| 703 | GSE20152\_SPHK1\_KO\_VS\_WT\_HTNFA\_OVERXPRESS\_ANKLE\_DN |  | 22 | 0.20 | 0.88 | 0.603 | 1.000 | 1.000 | 814 | tags=77%, list=58%, signal=180% |
| 704 | GSE6674\_ANTI\_IGM\_VS\_CPG\_STIM\_BCELL\_DN |  | 36 | 0.17 | 0.88 | 0.626 | 1.000 | 1.000 | 720 | tags=67%, list=51%, signal=133% |
| 705 | GSE26488\_WT\_VS\_VP16\_TRANSGENIC\_HDAC7\_KO\_DOUBLE\_POSITIVE\_THYMOCYTE\_UP |  | 18 | 0.21 | 0.88 | 0.613 | 1.000 | 1.000 | 410 | tags=39%, list=29%, signal=54% |
| 706 | GSE26488\_WT\_VS\_HDAC7\_KO\_DOUBLE\_POSITIVE\_THYMOCYTE\_UP |  | 17 | 0.22 | 0.88 | 0.642 | 1.000 | 1.000 | 286 | tags=29%, list=20%, signal=36% |
| 707 | GSE2770\_IL12\_AND\_TGFB\_ACT\_VS\_ACT\_CD4\_TCELL\_2H\_UP |  | 25 | 0.19 | 0.88 | 0.613 | 1.000 | 1.000 | 269 | tags=28%, list=19%, signal=34% |
| 708 | GSE22589\_HEALTHY\_VS\_SIV\_INFECTED\_DC\_UP |  | 17 | 0.22 | 0.88 | 0.601 | 1.000 | 1.000 | 207 | tags=29%, list=15%, signal=34% |
| 709 | GSE41087\_WT\_VS\_FOXP3\_MUT\_ANTI\_CD3\_CD28\_STIM\_CD4\_TCELL\_DN |  | 18 | 0.22 | 0.88 | 0.575 | 1.000 | 1.000 | 868 | tags=83%, list=62%, signal=214% |
| 710 | GSE11864\_CSF1\_VS\_CSF1\_IFNG\_IN\_MAC\_DN |  | 18 | 0.22 | 0.88 | 0.594 | 1.000 | 1.000 | 233 | tags=28%, list=17%, signal=33% |
| 711 | GSE26928\_CENTR\_MEMORY\_VS\_CXCR5\_POS\_CD4\_TCELL\_DN |  | 24 | 0.20 | 0.88 | 0.611 | 1.000 | 1.000 | 458 | tags=46%, list=32%, signal=67% |
| 712 | GSE22611\_UNSTIM\_VS\_2H\_MDP\_STIM\_MUTANT\_NOD2\_TRANSDUCED\_HEK293T\_CELL\_DN |  | 28 | 0.18 | 0.88 | 0.600 | 1.000 | 1.000 | 633 | tags=64%, list=45%, signal=114% |
| 713 | GSE41176\_UNSTIM\_VS\_ANTI\_IGM\_STIM\_BCELL\_6H\_UP |  | 16 | 0.23 | 0.88 | 0.599 | 1.000 | 1.000 | 457 | tags=50%, list=32%, signal=73% |
| 714 | GSE1791\_CTRL\_VS\_NEUROMEDINU\_IN\_T\_CELL\_LINE\_12H\_UP |  | 21 | 0.20 | 0.88 | 0.607 | 1.000 | 1.000 | 504 | tags=48%, list=36%, signal=73% |
| 715 | GSE20198\_UNTREATED\_VS\_IFNA\_TREATED\_ACT\_CD4\_TCELL\_DN |  | 18 | 0.22 | 0.88 | 0.596 | 1.000 | 1.000 | 549 | tags=56%, list=39%, signal=90% |
| 716 | GSE15324\_NAIVE\_VS\_ACTIVATED\_ELF4\_KO\_CD8\_TCELL\_UP |  | 28 | 0.18 | 0.88 | 0.630 | 1.000 | 1.000 | 163 | tags=21%, list=12%, signal=24% |
| 717 | GSE27786\_BCELL\_VS\_CD8\_TCELL\_UP |  | 19 | 0.21 | 0.88 | 0.618 | 1.000 | 1.000 | 207 | tags=26%, list=15%, signal=30% |
| 718 | GSE28726\_ACT\_CD4\_TCELL\_VS\_ACT\_NKTCELL\_UP |  | 24 | 0.19 | 0.88 | 0.590 | 1.000 | 1.000 | 989 | tags=92%, list=70%, signal=301% |
| 719 | GSE3039\_ALPHABETA\_CD8\_TCELL\_VS\_B1\_BCELL\_DN |  | 19 | 0.21 | 0.88 | 0.618 | 1.000 | 1.000 | 736 | tags=74%, list=52%, signal=152% |
| 720 | GSE16450\_CTRL\_VS\_IFNA\_12H\_STIM\_IMMATURE\_NEURON\_CELL\_LINE\_UP |  | 28 | 0.18 | 0.87 | 0.608 | 1.000 | 1.000 | 403 | tags=39%, list=29%, signal=54% |
| 721 | GSE23984\_CTRL\_VS\_HYPOCALEMIC\_VITAMIND\_ANALOG\_TCELL\_UP |  | 18 | 0.22 | 0.87 | 0.604 | 1.000 | 1.000 | 519 | tags=50%, list=37%, signal=78% |
| 722 | GSE27786\_CD4\_TCELL\_VS\_NKTCELL\_DN |  | 20 | 0.20 | 0.87 | 0.641 | 1.000 | 1.000 | 618 | tags=55%, list=44%, signal=96% |
| 723 | GSE8868\_SPLEEN\_VS\_INTESTINE\_CD11B\_POS\_CD11C\_NEG\_DC\_DN |  | 16 | 0.22 | 0.87 | 0.610 | 1.000 | 1.000 | 306 | tags=31%, list=22%, signal=39% |
| 724 | GSE45365\_HEALTHY\_VS\_MCMV\_INFECTION\_BCELL\_IFNAR\_KO\_UP |  | 21 | 0.20 | 0.87 | 0.638 | 1.000 | 1.000 | 733 | tags=71%, list=52%, signal=146% |
| 725 | GSE3982\_MEMORY\_CD4\_TCELL\_VS\_TH1\_DN |  | 27 | 0.19 | 0.87 | 0.623 | 1.000 | 1.000 | 953 | tags=89%, list=68%, signal=269% |
| 726 | GSE11924\_TH2\_VS\_TH17\_CD4\_TCELL\_DN |  | 20 | 0.20 | 0.87 | 0.631 | 1.000 | 1.000 | 161 | tags=20%, list=11%, signal=22% |
| 727 | GSE360\_L\_MAJOR\_VS\_B\_MALAYI\_LOW\_DOSE\_MAC\_DN |  | 26 | 0.19 | 0.87 | 0.635 | 1.000 | 1.000 | 838 | tags=81%, list=59%, signal=195% |
| 728 | KAECH\_NAIVE\_VS\_DAY8\_EFF\_CD8\_TCELL\_UP |  | 21 | 0.20 | 0.87 | 0.622 | 1.000 | 1.000 | 425 | tags=43%, list=30%, signal=60% |
| 729 | GSE17721\_POLYIC\_VS\_CPG\_8H\_BMDC\_DN |  | 20 | 0.20 | 0.87 | 0.620 | 1.000 | 1.000 | 874 | tags=85%, list=62%, signal=220% |
| 730 | GSE11973\_MIR223\_KOVS\_WT\_BONE\_MARROW\_NEUTROPHIL\_DN |  | 17 | 0.22 | 0.86 | 0.617 | 1.000 | 1.000 | 617 | tags=65%, list=44%, signal=114% |
| 731 | GSE40666\_WT\_VS\_STAT1\_KO\_CD8\_TCELL\_DN |  | 16 | 0.22 | 0.86 | 0.619 | 1.000 | 1.000 | 29 | tags=13%, list=2%, signal=13% |
| 732 | GSE21927\_C26GM\_VS\_4T1\_TUMOR\_MONOCYTE\_BALBC\_UP |  | 17 | 0.22 | 0.86 | 0.647 | 1.000 | 1.000 | 495 | tags=53%, list=35%, signal=81% |
| 733 | GSE3982\_EFF\_MEMORY\_CD4\_TCELL\_VS\_TH1\_DN |  | 26 | 0.19 | 0.86 | 0.619 | 1.000 | 1.000 | 890 | tags=85%, list=63%, signal=225% |
| 734 | GSE7509\_UNSTIM\_VS\_FCGRIIB\_STIM\_MONOCYTE\_DN |  | 18 | 0.22 | 0.86 | 0.621 | 1.000 | 1.000 | 221 | tags=28%, list=16%, signal=33% |
| 735 | GSE36009\_WT\_VS\_NLRP10\_KO\_DC\_LPS\_STIM\_UP |  | 19 | 0.21 | 0.86 | 0.642 | 1.000 | 1.000 | 184 | tags=21%, list=13%, signal=24% |
| 736 | GSE17974\_0H\_VS\_1H\_IN\_VITRO\_ACT\_CD4\_TCELL\_DN |  | 27 | 0.18 | 0.86 | 0.605 | 1.000 | 1.000 | 964 | tags=89%, list=68%, signal=275% |
| 737 | GSE3720\_VD1\_VS\_VD2\_GAMMADELTA\_TCELL\_WITH\_PMA\_STIM\_UP |  | 27 | 0.18 | 0.86 | 0.629 | 1.000 | 1.000 | 200 | tags=19%, list=14%, signal=21% |
| 738 | GSE5589\_IL6\_KO\_VS\_IL10\_KO\_LPS\_AND\_IL10\_STIM\_MACROPHAGE\_180MIN\_DN |  | 16 | 0.22 | 0.86 | 0.646 | 1.000 | 1.000 | 299 | tags=31%, list=21%, signal=39% |
| 739 | GSE22886\_NAIVE\_VS\_MEMORY\_TCELL\_UP |  | 15 | 0.23 | 0.86 | 0.647 | 1.000 | 1.000 | 525 | tags=53%, list=37%, signal=84% |
| 740 | GSE411\_WT\_VS\_SOCS3\_KO\_MACROPHAGE\_IL6\_STIM\_100MIN\_UP |  | 18 | 0.21 | 0.86 | 0.623 | 1.000 | 1.000 | 591 | tags=56%, list=42%, signal=94% |
| 741 | GSE11864\_UNTREATED\_VS\_CSF1\_PAM3CYS\_IN\_MAC\_DN |  | 26 | 0.19 | 0.86 | 0.630 | 1.000 | 1.000 | 1006 | tags=92%, list=71%, signal=316% |
| 742 | GSE17721\_0.5H\_VS\_24H\_PAM3CSK4\_BMDC\_DN |  | 25 | 0.19 | 0.86 | 0.639 | 1.000 | 1.000 | 133 | tags=20%, list=9%, signal=22% |
| 743 | GSE15735\_CTRL\_VS\_HDAC\_INHIBITOR\_TREATED\_CD4\_TCELL\_12H\_DN |  | 17 | 0.21 | 0.86 | 0.663 | 1.000 | 1.000 | 743 | tags=71%, list=53%, signal=147% |
| 744 | GSE557\_WT\_VS\_CIITA\_KO\_DC\_UP |  | 22 | 0.19 | 0.86 | 0.639 | 1.000 | 1.000 | 409 | tags=41%, list=29%, signal=57% |
| 745 | GSE12366\_GC\_BCELL\_VS\_PLASMA\_CELL\_UP |  | 25 | 0.19 | 0.86 | 0.633 | 1.000 | 1.000 | 588 | tags=56%, list=42%, signal=94% |
| 746 | GSE25502\_WT\_VS\_KLF13\_KO\_THYMIC\_MEMORY\_LIKE\_CD8\_TCELL\_UP |  | 17 | 0.21 | 0.86 | 0.615 | 1.000 | 1.000 | 261 | tags=29%, list=18%, signal=36% |
| 747 | GSE2197\_IMMUNOSUPPRESSIVE\_DNA\_VS\_UNTREATEDIN\_DC\_DN |  | 24 | 0.19 | 0.86 | 0.636 | 1.000 | 1.000 | 592 | tags=58%, list=42%, signal=99% |
| 748 | GSE31082\_DP\_VS\_CD4\_SP\_THYMOCYTE\_UP |  | 19 | 0.21 | 0.86 | 0.618 | 1.000 | 1.000 | 421 | tags=47%, list=30%, signal=67% |
| 749 | GSE27786\_LIN\_NEG\_VS\_BCELL\_UP |  | 24 | 0.19 | 0.86 | 0.625 | 1.000 | 1.000 | 727 | tags=71%, list=52%, signal=144% |
| 750 | GSE27786\_BCELL\_VS\_NKCELL\_UP |  | 16 | 0.22 | 0.86 | 0.622 | 1.000 | 1.000 | 430 | tags=44%, list=30%, signal=62% |
| 751 | GSE9006\_HEALTHY\_VS\_TYPE\_1\_DIABETES\_PBMC\_1MONTH\_POST\_DX\_UP |  | 23 | 0.19 | 0.86 | 0.639 | 1.000 | 1.000 | 89 | tags=13%, list=6%, signal=14% |
| 752 | GSE17721\_PAM3CSK4\_VS\_GADIQUIMOD\_4H\_BMDC\_UP |  | 15 | 0.22 | 0.86 | 0.640 | 1.000 | 1.000 | 308 | tags=33%, list=22%, signal=42% |
| 753 | GSE20366\_TREG\_VS\_NAIVE\_CD4\_TCELL\_DEC205\_CONVERSION\_DN |  | 25 | 0.19 | 0.86 | 0.648 | 1.000 | 1.000 | 100 | tags=16%, list=7%, signal=17% |
| 754 | GSE11961\_MEMORY\_BCELL\_DAY7\_VS\_GERMINAL\_CENTER\_BCELL\_DAY40\_UP |  | 17 | 0.21 | 0.85 | 0.619 | 1.000 | 1.000 | 1020 | tags=94%, list=72%, signal=336% |
| 755 | GSE22886\_NAIVE\_CD4\_TCELL\_VS\_NKCELL\_UP |  | 15 | 0.22 | 0.85 | 0.648 | 1.000 | 1.000 | 126 | tags=20%, list=9%, signal=22% |
| 756 | GSE24210\_TCONV\_VS\_TREG\_DN |  | 19 | 0.20 | 0.85 | 0.650 | 1.000 | 1.000 | 17 | tags=11%, list=1%, signal=11% |
| 757 | GSE40225\_WT\_VS\_RIP\_B7X\_DIABETIC\_MOUSE\_PANCREATIC\_CD8\_TCELL\_DN |  | 25 | 0.19 | 0.85 | 0.653 | 1.000 | 1.000 | 956 | tags=88%, list=68%, signal=268% |
| 758 | GSE45739\_NRAS\_KO\_VS\_WT\_UNSTIM\_CD4\_TCELL\_UP |  | 28 | 0.18 | 0.85 | 0.635 | 1.000 | 1.000 | 412 | tags=36%, list=29%, signal=49% |
| 759 | GSE28783\_CTRL\_ANTI\_MIR\_VS\_UNTREATED\_ATHEROSCLEROSIS\_MACROPHAGE\_UP |  | 16 | 0.22 | 0.85 | 0.653 | 1.000 | 1.000 | 329 | tags=38%, list=23%, signal=48% |
| 760 | GSE21927\_UNTREATED\_VS\_GMCSF\_GCSF\_TREATED\_BONE\_MARROW\_UP |  | 17 | 0.22 | 0.85 | 0.650 | 1.000 | 1.000 | 376 | tags=41%, list=27%, signal=55% |
| 761 | GSE37301\_PRO\_BCELL\_VS\_CD4\_TCELL\_UP |  | 19 | 0.21 | 0.85 | 0.647 | 1.000 | 1.000 | 310 | tags=32%, list=22%, signal=40% |
| 762 | GSE17974\_CTRL\_VS\_ACT\_IL4\_AND\_ANTI\_IL12\_1H\_CD4\_TCELL\_UP |  | 16 | 0.22 | 0.85 | 0.671 | 1.000 | 1.000 | 83 | tags=19%, list=6%, signal=20% |
| 763 | GSE2770\_IL12\_VS\_IL4\_TREATED\_ACT\_CD4\_TCELL\_6H\_DN |  | 20 | 0.20 | 0.85 | 0.650 | 1.000 | 1.000 | 407 | tags=40%, list=29%, signal=55% |
| 764 | GSE4590\_PRE\_BCELL\_VS\_LARGE\_PRE\_BCELL\_DN |  | 17 | 0.21 | 0.85 | 0.630 | 1.000 | 1.000 | 669 | tags=65%, list=47%, signal=122% |
| 765 | GSE2770\_IL4\_ACT\_VS\_ACT\_CD4\_TCELL\_6H\_DN |  | 29 | 0.18 | 0.85 | 0.658 | 1.000 | 1.000 | 410 | tags=34%, list=29%, signal=48% |
| 766 | GSE12001\_MIR223\_KO\_VS\_WT\_NEUTROPHIL\_UP |  | 19 | 0.20 | 0.85 | 0.640 | 1.000 | 1.000 | 132 | tags=21%, list=9%, signal=23% |
| 767 | GSE20727\_DNFB\_ALLERGEN\_VS\_ROS\_INH\_AND\_DNFB\_ALLERGEN\_TREATED\_DC\_DN |  | 19 | 0.20 | 0.84 | 0.659 | 1.000 | 1.000 | 177 | tags=21%, list=13%, signal=24% |
| 768 | GSE46606\_IRF4HIGH\_VS\_IRF4MID\_CD40L\_IL2\_IL5\_DAY1\_STIMULATED\_BCELL\_DN |  | 15 | 0.22 | 0.84 | 0.656 | 1.000 | 1.000 | 351 | tags=40%, list=25%, signal=53% |
| 769 | GSE12845\_IGD\_POS\_VS\_NEG\_BLOOD\_BCELL\_DN |  | 28 | 0.18 | 0.84 | 0.683 | 1.000 | 1.000 | 1098 | tags=96%, list=78%, signal=426% |
| 770 | GSE17721\_CTRL\_VS\_POLYIC\_6H\_BMDC\_UP |  | 15 | 0.22 | 0.84 | 0.652 | 1.000 | 1.000 | 393 | tags=40%, list=28%, signal=55% |
| 771 | GSE22886\_NAIVE\_BCELL\_VS\_BM\_PLASMA\_CELL\_UP |  | 22 | 0.20 | 0.84 | 0.676 | 1.000 | 1.000 | 373 | tags=36%, list=26%, signal=49% |
| 772 | GSE9006\_HEALTHY\_VS\_TYPE\_2\_DIABETES\_PBMC\_AT\_DX\_UP |  | 17 | 0.21 | 0.84 | 0.640 | 1.000 | 1.000 | 212 | tags=24%, list=15%, signal=27% |
| 773 | GSE22886\_CTRL\_VS\_LPS\_24H\_DC\_UP |  | 16 | 0.21 | 0.84 | 0.653 | 1.000 | 1.000 | 907 | tags=94%, list=64%, signal=259% |
| 774 | GSE360\_CTRL\_VS\_L\_DONOVANI\_DC\_UP |  | 23 | 0.19 | 0.84 | 0.661 | 1.000 | 1.000 | 533 | tags=48%, list=38%, signal=76% |
| 775 | GSE21927\_SPLENIC\_VS\_TUMOR\_MONOCYTES\_FROM\_C26GM\_TUMOROUS\_MICE\_BALBC\_DN |  | 23 | 0.19 | 0.84 | 0.663 | 1.000 | 1.000 | 10 | tags=9%, list=1%, signal=9% |
| 776 | GSE36392\_TYPE\_2\_MYELOID\_VS\_MAC\_IL25\_TREATED\_LUNG\_DN |  | 28 | 0.18 | 0.84 | 0.649 | 1.000 | 1.000 | 638 | tags=57%, list=45%, signal=102% |
| 777 | GSE7852\_TREG\_VS\_TCONV\_DN |  | 19 | 0.20 | 0.84 | 0.668 | 1.000 | 1.000 | 269 | tags=32%, list=19%, signal=38% |
| 778 | GSE13173\_UNTREATED\_VS\_IL12\_TREATED\_ACT\_CD8\_TCELL\_DN |  | 18 | 0.21 | 0.84 | 0.662 | 1.000 | 1.000 | 353 | tags=33%, list=25%, signal=44% |
| 779 | GSE17721\_CTRL\_VS\_PAM3CSK4\_6H\_BMDC\_DN |  | 26 | 0.18 | 0.84 | 0.674 | 1.000 | 1.000 | 613 | tags=58%, list=43%, signal=100% |
| 780 | GSE18281\_SUBCAPSULAR\_CORTICAL\_REGION\_VS\_WHOLE\_CORTEX\_THYMUS\_DN |  | 16 | 0.21 | 0.84 | 0.651 | 1.000 | 1.000 | 498 | tags=50%, list=35%, signal=76% |
| 781 | GSE12505\_WT\_VS\_E2\_2\_HET\_PDC\_DN |  | 21 | 0.19 | 0.84 | 0.657 | 1.000 | 1.000 | 345 | tags=33%, list=24%, signal=43% |
| 782 | GSE24671\_CTRL\_VS\_SENDAI\_VIRUS\_INFECTED\_MOUSE\_SPLENOCYTES\_DN |  | 15 | 0.22 | 0.84 | 0.667 | 1.000 | 1.000 | 612 | tags=60%, list=43%, signal=105% |
| 783 | GSE17721\_CTRL\_VS\_LPS\_0.5H\_BMDC\_UP |  | 17 | 0.21 | 0.84 | 0.660 | 1.000 | 1.000 | 255 | tags=29%, list=18%, signal=35% |
| 784 | GSE19401\_NAIVE\_VS\_IMMUNIZED\_MOUSE\_PLN\_FOLLICULAR\_DC\_DN |  | 21 | 0.19 | 0.83 | 0.656 | 1.000 | 1.000 | 78 | tags=14%, list=6%, signal=15% |
| 785 | GSE360\_L\_DONOVANI\_VS\_T\_GONDII\_MAC\_UP |  | 20 | 0.20 | 0.83 | 0.657 | 1.000 | 1.000 | 380 | tags=40%, list=27%, signal=54% |
| 786 | GSE40274\_CTRL\_VS\_XBP1\_TRANSDUCED\_ACTIVATED\_CD4\_TCELL\_DN |  | 23 | 0.19 | 0.83 | 0.663 | 1.000 | 1.000 | 818 | tags=78%, list=58%, signal=183% |
| 787 | GSE18791\_CTRL\_VS\_NEWCASTLE\_VIRUS\_DC\_6H\_DN |  | 27 | 0.17 | 0.83 | 0.679 | 1.000 | 1.000 | 263 | tags=26%, list=19%, signal=31% |
| 788 | GSE40274\_CTRL\_VS\_FOXP3\_AND\_XBP1\_TRANSDUCED\_ACTIVATED\_CD4\_TCELL\_UP |  | 24 | 0.19 | 0.83 | 0.667 | 1.000 | 1.000 | 56 | tags=13%, list=4%, signal=13% |
| 789 | GSE10240\_IL22\_VS\_IL17\_STIM\_PRIMARY\_BRONCHIAL\_EPITHELIAL\_CELLS\_UP |  | 23 | 0.18 | 0.83 | 0.675 | 1.000 | 1.000 | 336 | tags=30%, list=24%, signal=39% |
| 790 | GSE2770\_TGFB\_AND\_IL4\_ACT\_VS\_ACT\_CD4\_TCELL\_48H\_UP |  | 19 | 0.20 | 0.83 | 0.686 | 1.000 | 1.000 | 528 | tags=47%, list=37%, signal=75% |
| 791 | GSE10240\_CTRL\_VS\_IL17\_AND\_IL22\_STIM\_PRIMARY\_BRONCHIAL\_EPITHELIAL\_CELLS\_UP |  | 23 | 0.18 | 0.83 | 0.676 | 1.000 | 1.000 | 686 | tags=61%, list=49%, signal=117% |
| 792 | GSE3982\_BASOPHIL\_VS\_NKCELL\_DN |  | 18 | 0.20 | 0.83 | 0.691 | 1.000 | 1.000 | 747 | tags=72%, list=53%, signal=152% |
| 793 | GSE2770\_IL12\_VS\_TGFB\_AND\_IL12\_TREATED\_ACT\_CD4\_TCELL\_48H\_UP |  | 20 | 0.20 | 0.83 | 0.675 | 1.000 | 1.000 | 73 | tags=15%, list=5%, signal=16% |
| 794 | GSE6269\_HEALTHY\_VS\_FLU\_INF\_PBMC\_UP |  | 16 | 0.21 | 0.83 | 0.662 | 1.000 | 1.000 | 38 | tags=13%, list=3%, signal=13% |
| 795 | GSE27786\_LSK\_VS\_NKTCELL\_UP |  | 23 | 0.19 | 0.83 | 0.655 | 1.000 | 1.000 | 780 | tags=74%, list=55%, signal=163% |
| 796 | GSE339\_CD8POS\_VS\_CD4CD8DN\_DC\_UP |  | 18 | 0.20 | 0.83 | 0.654 | 1.000 | 1.000 | 596 | tags=56%, list=42%, signal=95% |
| 797 | GSE24102\_GRANULOCYSTIC\_MDSC\_VS\_NEUTROPHIL\_DN |  | 21 | 0.19 | 0.83 | 0.685 | 1.000 | 1.000 | 518 | tags=48%, list=37%, signal=74% |
| 798 | GSE14308\_TH17\_VS\_NAIVE\_CD4\_TCELL\_DN |  | 18 | 0.20 | 0.83 | 0.689 | 1.000 | 1.000 | 627 | tags=67%, list=44%, signal=118% |
| 799 | GSE29618\_LAIV\_VS\_TIV\_FLU\_VACCINE\_DAY7\_MDC\_DN |  | 15 | 0.21 | 0.83 | 0.672 | 1.000 | 1.000 | 324 | tags=33%, list=23%, signal=43% |
| 800 | GSE13306\_TREG\_VS\_TCONV\_LAMINA\_PROPRIA\_DN |  | 17 | 0.21 | 0.83 | 0.652 | 1.000 | 1.000 | 174 | tags=24%, list=12%, signal=27% |
| 801 | GSE3982\_EFF\_MEMORY\_CD4\_TCELL\_VS\_NKCELL\_UP |  | 17 | 0.20 | 0.83 | 0.688 | 1.000 | 1.000 | 857 | tags=82%, list=61%, signal=207% |
| 802 | GSE3982\_EOSINOPHIL\_VS\_BASOPHIL\_DN |  | 29 | 0.17 | 0.82 | 0.679 | 1.000 | 1.000 | 78 | tags=14%, list=6%, signal=14% |
| 803 | GSE14415\_FOXP3\_KO\_NATURAL\_TREG\_VS\_TCONV\_UP |  | 16 | 0.21 | 0.82 | 0.672 | 1.000 | 1.000 | 261 | tags=31%, list=18%, signal=38% |
| 804 | GSE5455\_HEALTHY\_VS\_TUMOR\_BEARING\_MOUSE\_SPLEEN\_MONOCYTE\_24H\_INCUBATION\_UP |  | 20 | 0.20 | 0.82 | 0.662 | 1.000 | 1.000 | 480 | tags=45%, list=34%, signal=67% |
| 805 | GSE21063\_CTRL\_VS\_ANTI\_IGM\_STIM\_BCELL\_NFATC1\_KO\_8H\_DN |  | 17 | 0.21 | 0.82 | 0.694 | 1.000 | 1.000 | 457 | tags=47%, list=32%, signal=69% |
| 806 | GSE37301\_RAG2\_KO\_VS\_RAG2\_AND\_ETS1\_KO\_NK\_CELL\_DN |  | 16 | 0.21 | 0.82 | 0.687 | 1.000 | 1.000 | 375 | tags=38%, list=27%, signal=50% |
| 807 | GSE23505\_UNTREATED\_VS\_4DAY\_IL6\_IL1\_TREATED\_CD4\_TCELL\_UP |  | 16 | 0.21 | 0.82 | 0.672 | 1.000 | 1.000 | 542 | tags=50%, list=38%, signal=80% |
| 808 | GSE27786\_BCELL\_VS\_CD8\_TCELL\_DN |  | 20 | 0.19 | 0.82 | 0.684 | 1.000 | 1.000 | 414 | tags=45%, list=29%, signal=63% |
| 809 | GSE23502\_WT\_VS\_HDC\_KO\_MYELOID\_DERIVED\_SUPPRESSOR\_CELL\_BM\_DN |  | 20 | 0.19 | 0.82 | 0.708 | 1.000 | 1.000 | 233 | tags=25%, list=17%, signal=30% |
| 810 | GSE20366\_EX\_VIVO\_VS\_DEC205\_CONVERSION\_UP |  | 28 | 0.17 | 0.82 | 0.682 | 1.000 | 1.000 | 584 | tags=46%, list=41%, signal=78% |
| 811 | GSE21546\_UNSTIM\_VS\_ANTI\_CD3\_STIM\_ELK1\_KO\_DP\_THYMOCYTES\_DN |  | 21 | 0.19 | 0.82 | 0.685 | 1.000 | 1.000 | 265 | tags=29%, list=19%, signal=35% |
| 812 | GSE21063\_3H\_VS\_16H\_ANTI\_IGM\_STIM\_NFATC1\_KOBCELL\_DN |  | 16 | 0.21 | 0.82 | 0.672 | 1.000 | 1.000 | 208 | tags=25%, list=15%, signal=29% |
| 813 | GSE32533\_WT\_VS\_MIR17\_OVEREXPRESS\_ACT\_CD4\_TCELL\_UP |  | 34 | 0.16 | 0.82 | 0.680 | 1.000 | 1.000 | 372 | tags=32%, list=26%, signal=43% |
| 814 | GSE19401\_RETINOIC\_ACID\_VS\_RETINOIC\_ACID\_AND\_PAM2CSK4\_STIM\_FOLLICULAR\_DC\_DN |  | 24 | 0.18 | 0.82 | 0.694 | 1.000 | 1.000 | 184 | tags=21%, list=13%, signal=24% |
| 815 | GSE40443\_INDUCED\_VS\_TOTAL\_TREG\_DN |  | 15 | 0.22 | 0.82 | 0.699 | 1.000 | 1.000 | 203 | tags=27%, list=14%, signal=31% |
| 816 | GSE2585\_THYMIC\_DC\_VS\_THYMIC\_MACROPHAGE\_DN |  | 15 | 0.21 | 0.82 | 0.668 | 1.000 | 1.000 | 1114 | tags=100%, list=79%, signal=470% |
| 817 | GSE22103\_UNSTIM\_VS\_GMCSF\_AND\_IFNG\_STIM\_NEUTROPHIL\_UP |  | 28 | 0.18 | 0.82 | 0.699 | 1.000 | 1.000 | 415 | tags=36%, list=29%, signal=50% |
| 818 | GSE3982\_BCELL\_VS\_BASOPHIL\_UP |  | 24 | 0.18 | 0.82 | 0.681 | 1.000 | 1.000 | 690 | tags=67%, list=49%, signal=128% |
| 819 | GSE411\_WT\_VS\_SOCS3\_KO\_MACROPHAGE\_DN |  | 22 | 0.19 | 0.82 | 0.704 | 1.000 | 1.000 | 907 | tags=86%, list=64%, signal=238% |
| 820 | GSE43863\_NAIVE\_VS\_MEMORY\_TH1\_CD4\_TCELL\_D150\_LCMV\_UP |  | 15 | 0.22 | 0.82 | 0.681 | 1.000 | 1.000 | 227 | tags=27%, list=16%, signal=31% |
| 821 | GSE12845\_IGD\_POS\_BLOOD\_VS\_PRE\_GC\_TONSIL\_BCELL\_DN |  | 29 | 0.17 | 0.82 | 0.702 | 1.000 | 1.000 | 484 | tags=41%, list=34%, signal=62% |
| 822 | GSE20366\_EX\_VIVO\_VS\_HOMEOSTATIC\_CONVERSION\_TREG\_UP |  | 27 | 0.17 | 0.82 | 0.696 | 1.000 | 1.000 | 46 | tags=11%, list=3%, signal=11% |
| 823 | GSE13306\_RA\_VS\_UNTREATED\_TCONV\_UP |  | 24 | 0.18 | 0.81 | 0.704 | 1.000 | 1.000 | 670 | tags=67%, list=47%, signal=125% |
| 824 | GSE3720\_UNSTIM\_VS\_LPS\_STIM\_VD1\_GAMMADELTA\_TCELL\_UP |  | 23 | 0.18 | 0.81 | 0.699 | 1.000 | 1.000 | 176 | tags=22%, list=12%, signal=24% |
| 825 | GSE36078\_WT\_VS\_IL1R\_KO\_LUNG\_DC\_AFTER\_AD5\_T425A\_HEXON\_INF\_UP |  | 23 | 0.19 | 0.81 | 0.686 | 1.000 | 1.000 | 175 | tags=17%, list=12%, signal=20% |
| 826 | GSE27241\_WT\_VS\_RORGT\_KO\_TH17\_POLARIZED\_CD4\_TCELL\_UP |  | 15 | 0.22 | 0.81 | 0.687 | 1.000 | 1.000 | 177 | tags=20%, list=13%, signal=23% |
| 827 | GSE17721\_POLYIC\_VS\_CPG\_2H\_BMDC\_DN |  | 19 | 0.20 | 0.81 | 0.697 | 1.000 | 1.000 | 193 | tags=21%, list=14%, signal=24% |
| 828 | GSE24026\_PD1\_LIGATION\_VS\_CTRL\_IN\_ACT\_TCELL\_LINE\_DN |  | 22 | 0.19 | 0.81 | 0.707 | 1.000 | 1.000 | 728 | tags=68%, list=52%, signal=139% |
| 829 | GSE30971\_2H\_VS\_4H\_LPS\_STIM\_MACROPHAGE\_WBP7\_KO\_DN |  | 18 | 0.20 | 0.81 | 0.698 | 1.000 | 1.000 | 448 | tags=44%, list=32%, signal=64% |
| 830 | GSE23505\_IL6\_IL1\_IL23\_VS\_IL6\_IL1\_TGFB\_TREATED\_CD4\_TCELL\_UP |  | 15 | 0.22 | 0.81 | 0.690 | 1.000 | 1.000 | 289 | tags=33%, list=20%, signal=41% |
| 831 | GSE18893\_CTRL\_VS\_TNF\_TREATED\_TCONV\_2H\_UP |  | 18 | 0.20 | 0.81 | 0.688 | 1.000 | 1.000 | 277 | tags=28%, list=20%, signal=34% |
| 832 | GSE411\_WT\_VS\_SOCS3\_KO\_MACROPHAGE\_IL6\_STIM\_400MIN\_DN |  | 23 | 0.18 | 0.81 | 0.703 | 1.000 | 1.000 | 266 | tags=26%, list=19%, signal=32% |
| 833 | GSE15930\_NAIVE\_VS\_24H\_IN\_VITRO\_STIM\_INFAB\_CD8\_TCELL\_DN |  | 21 | 0.19 | 0.81 | 0.748 | 1.000 | 1.000 | 1022 | tags=95%, list=72%, signal=340% |
| 834 | GSE19401\_PLN\_VS\_PEYERS\_PATCH\_FOLLICULAR\_DC\_DN |  | 19 | 0.20 | 0.81 | 0.694 | 1.000 | 1.000 | 89 | tags=16%, list=6%, signal=17% |
| 835 | GSE3982\_NKCELL\_VS\_TH2\_UP |  | 21 | 0.19 | 0.81 | 0.707 | 1.000 | 1.000 | 536 | tags=52%, list=38%, signal=83% |
| 836 | GSE17721\_POLYIC\_VS\_GARDIQUIMOD\_1H\_BMDC\_DN |  | 21 | 0.19 | 0.81 | 0.684 | 1.000 | 1.000 | 39 | tags=10%, list=3%, signal=10% |
| 837 | GSE22527\_ANTI\_CD3\_INVIVO\_VS\_UNTREATED\_MOUSE\_TREG\_DN |  | 30 | 0.16 | 0.81 | 0.702 | 1.000 | 1.000 | 747 | tags=70%, list=53%, signal=146% |
| 838 | GSE26669\_CTRL\_VS\_COSTIM\_BLOCK\_MLR\_CD4\_TCELL\_DN |  | 17 | 0.20 | 0.81 | 0.742 | 1.000 | 1.000 | 362 | tags=35%, list=26%, signal=47% |
| 839 | GSE360\_DC\_VS\_MAC\_B\_MALAYI\_HIGH\_DOSE\_UP |  | 19 | 0.20 | 0.81 | 0.672 | 1.000 | 1.000 | 14 | tags=11%, list=1%, signal=10% |
| 840 | GSE37301\_COMMON\_LYMPHOID\_PROGENITOR\_VS\_PRO\_BCELL\_UP |  | 16 | 0.20 | 0.81 | 0.687 | 1.000 | 1.000 | 1011 | tags=94%, list=72%, signal=327% |
| 841 | GSE30153\_LUPUS\_VS\_HEALTHY\_DONOR\_BCELL\_UP |  | 21 | 0.18 | 0.80 | 0.712 | 1.000 | 1.000 | 355 | tags=33%, list=25%, signal=44% |
| 842 | GSE42021\_CD24HI\_VS\_CD24LOW\_TREG\_THYMUS\_UP |  | 22 | 0.19 | 0.80 | 0.711 | 1.000 | 1.000 | 665 | tags=64%, list=47%, signal=118% |
| 843 | GSE6269\_HEALTHY\_VS\_STAPH\_PNEUMO\_INF\_PBMC\_UP |  | 16 | 0.20 | 0.80 | 0.716 | 1.000 | 1.000 | 335 | tags=31%, list=24%, signal=41% |
| 844 | GSE29618\_PRE\_VS\_DAY7\_FLU\_VACCINE\_MDC\_DN |  | 21 | 0.18 | 0.80 | 0.712 | 1.000 | 1.000 | 992 | tags=90%, list=70%, signal=300% |
| 845 | GSE2706\_LPS\_VS\_R848\_AND\_LPS\_8H\_STIM\_DC\_UP |  | 19 | 0.19 | 0.80 | 0.699 | 1.000 | 1.000 | 608 | tags=58%, list=43%, signal=100% |
| 846 | GSE17974\_0H\_VS\_6H\_IN\_VITRO\_ACT\_CD4\_TCELL\_DN |  | 32 | 0.16 | 0.80 | 0.709 | 1.000 | 1.000 | 597 | tags=53%, list=42%, signal=90% |
| 847 | GSE21546\_WT\_VS\_SAP1A\_KO\_AND\_ELK1\_KO\_DP\_THYMOCYTES\_DN |  | 26 | 0.17 | 0.80 | 0.703 | 1.000 | 1.000 | 212 | tags=23%, list=15%, signal=27% |
| 848 | GSE7831\_UNSTIM\_VS\_INFLUENZA\_STIM\_PDC\_4H\_UP |  | 19 | 0.19 | 0.80 | 0.701 | 1.000 | 1.000 | 26 | tags=11%, list=2%, signal=11% |
| 849 | GSE37532\_TREG\_VS\_TCONV\_PPARG\_KO\_CD4\_TCELL\_FROM\_LN\_DN |  | 35 | 0.15 | 0.80 | 0.745 | 1.000 | 1.000 | 800 | tags=71%, list=57%, signal=161% |
| 850 | GSE43955\_1H\_VS\_60H\_ACT\_CD4\_TCELL\_WITH\_TGFB\_IL6\_DN |  | 18 | 0.20 | 0.80 | 0.713 | 1.000 | 1.000 | 124 | tags=17%, list=9%, signal=18% |
| 851 | GSE28737\_BCL6\_HET\_VS\_BCL6\_KO\_FOLLICULAR\_BCELL\_UP |  | 19 | 0.19 | 0.80 | 0.724 | 1.000 | 1.000 | 184 | tags=21%, list=13%, signal=24% |
| 852 | GSE31082\_DP\_VS\_CD8\_SP\_THYMOCYTE\_DN |  | 20 | 0.19 | 0.80 | 0.703 | 1.000 | 1.000 | 209 | tags=25%, list=15%, signal=29% |
| 853 | GSE6092\_UNSTIM\_VS\_IFNG\_STIM\_ENDOTHELIAL\_CELL\_UP |  | 22 | 0.18 | 0.80 | 0.717 | 1.000 | 1.000 | 362 | tags=36%, list=26%, signal=48% |
| 854 | GSE17721\_PAM3CSK4\_VS\_GADIQUIMOD\_24H\_BMDC\_UP |  | 23 | 0.17 | 0.80 | 0.715 | 1.000 | 1.000 | 229 | tags=22%, list=16%, signal=26% |
| 855 | GSE17721\_CTRL\_VS\_GARDIQUIMOD\_1H\_BMDC\_DN |  | 23 | 0.18 | 0.80 | 0.717 | 1.000 | 1.000 | 153 | tags=17%, list=11%, signal=19% |
| 856 | GSE9509\_10MIN\_VS\_30MIN\_LPS\_STIM\_IL10\_KO\_MACROPHAGE\_UP |  | 19 | 0.19 | 0.80 | 0.698 | 1.000 | 1.000 | 1045 | tags=95%, list=74%, signal=360% |
| 857 | GSE14415\_ACT\_VS\_CTRL\_NATURAL\_TREG\_DN |  | 27 | 0.17 | 0.80 | 0.734 | 1.000 | 1.000 | 261 | tags=26%, list=18%, signal=31% |
| 858 | GSE37532\_TREG\_VS\_TCONV\_PPARG\_KO\_CD4\_TCELL\_FROM\_LN\_UP |  | 22 | 0.18 | 0.80 | 0.714 | 1.000 | 1.000 | 166 | tags=18%, list=12%, signal=20% |
| 859 | GSE5589\_LPS\_VS\_LPS\_AND\_IL6\_STIM\_IL10\_KO\_MACROPHAGE\_45MIN\_DN |  | 23 | 0.18 | 0.80 | 0.692 | 1.000 | 1.000 | 856 | tags=83%, list=61%, signal=207% |
| 860 | GSE17721\_0.5H\_VS\_24H\_GARDIQUIMOD\_BMDC\_UP |  | 15 | 0.21 | 0.79 | 0.725 | 1.000 | 1.000 | 160 | tags=20%, list=11%, signal=22% |
| 861 | GSE17974\_CTRL\_VS\_ACT\_IL4\_AND\_ANTI\_IL12\_2H\_CD4\_TCELL\_DN |  | 33 | 0.16 | 0.79 | 0.720 | 1.000 | 1.000 | 301 | tags=30%, list=21%, signal=38% |
| 862 | GSE17721\_CTRL\_VS\_PAM3CSK4\_4H\_BMDC\_UP |  | 20 | 0.19 | 0.79 | 0.722 | 1.000 | 1.000 | 1150 | tags=100%, list=82%, signal=533% |
| 863 | GSE17721\_POLYIC\_VS\_PAM3CSK4\_4H\_BMDC\_DN |  | 24 | 0.17 | 0.79 | 0.732 | 1.000 | 1.000 | 265 | tags=25%, list=19%, signal=30% |
| 864 | GSE34006\_A2AR\_KO\_VS\_A2AR\_AGONIST\_TREATED\_TREG\_UP |  | 18 | 0.20 | 0.79 | 0.744 | 1.000 | 1.000 | 541 | tags=50%, list=38%, signal=80% |
| 865 | GSE5142\_CTRL\_VS\_HTERT\_TRANSDUCED\_CD8\_TCELL\_EARLY\_PASSAGE\_CLONE\_DN |  | 21 | 0.18 | 0.79 | 0.706 | 1.000 | 1.000 | 631 | tags=62%, list=45%, signal=110% |
| 866 | GSE27859\_MACROPHAGE\_VS\_CD11C\_INT\_F480\_HI\_MACROPHAGE\_UP |  | 22 | 0.18 | 0.79 | 0.740 | 1.000 | 1.000 | 353 | tags=32%, list=25%, signal=42% |
| 867 | GSE37533\_PPARG1\_FOXP3\_VS\_FOXP3\_TRANSDUCED\_CD4\_TCELL\_PIOGLITAZONE\_TREATED\_DN |  | 33 | 0.16 | 0.79 | 0.748 | 1.000 | 1.000 | 282 | tags=27%, list=20%, signal=33% |
| 868 | GSE2770\_UNTREATED\_VS\_IL12\_TREATED\_ACT\_CD4\_TCELL\_2H\_DN |  | 17 | 0.20 | 0.79 | 0.732 | 1.000 | 1.000 | 207 | tags=24%, list=15%, signal=27% |
| 869 | GSE3039\_ALPHAALPHA\_CD8\_TCELL\_VS\_B1\_BCELL\_UP |  | 17 | 0.20 | 0.79 | 0.722 | 1.000 | 1.000 | 138 | tags=18%, list=10%, signal=19% |
| 870 | GSE18791\_UNSTIM\_VS\_NEWCATSLE\_VIRUS\_DC\_6H\_UP |  | 23 | 0.18 | 0.79 | 0.729 | 1.000 | 1.000 | 345 | tags=39%, list=24%, signal=51% |
| 871 | GSE43863\_NAIVE\_VS\_TFH\_CD4\_EFF\_TCELL\_D6\_LCMV\_UP |  | 32 | 0.16 | 0.79 | 0.725 | 1.000 | 1.000 | 558 | tags=47%, list=40%, signal=76% |
| 872 | GSE40666\_NAIVE\_VS\_EFFECTOR\_CD8\_TCELL\_UP |  | 18 | 0.19 | 0.79 | 0.736 | 1.000 | 1.000 | 28 | tags=11%, list=2%, signal=11% |
| 873 | GSE18281\_MEDULLARY\_THYMOCYTE\_VS\_WHOLE\_MEDULLA\_THYMUS\_DN |  | 20 | 0.19 | 0.79 | 0.723 | 1.000 | 1.000 | 281 | tags=30%, list=20%, signal=37% |
| 874 | GSE28737\_FOLLICULAR\_VS\_MARGINAL\_ZONE\_BCELL\_BCL6\_HET\_DN |  | 17 | 0.20 | 0.79 | 0.750 | 1.000 | 1.000 | 306 | tags=35%, list=22%, signal=45% |
| 875 | GSE40274\_CTRL\_VS\_FOXP3\_AND\_SATB1\_TRANSDUCED\_ACTIVATED\_CD4\_TCELL\_UP |  | 27 | 0.17 | 0.79 | 0.727 | 1.000 | 1.000 | 835 | tags=74%, list=59%, signal=178% |
| 876 | GSE2770\_IL12\_AND\_TGFB\_ACT\_VS\_ACT\_CD4\_TCELL\_48H\_UP |  | 16 | 0.20 | 0.79 | 0.736 | 1.000 | 1.000 | 449 | tags=44%, list=32%, signal=63% |
| 877 | GSE36888\_UNTREATED\_VS\_IL2\_TREATED\_TCELL\_17H\_UP |  | 15 | 0.21 | 0.79 | 0.725 | 1.000 | 1.000 | 1123 | tags=100%, list=80%, signal=485% |
| 878 | GSE32986\_GMCSF\_AND\_CURDLAN\_LOWDOSE\_VS\_GMCSF\_AND\_CURDLAN\_HIGHDOSE\_STIM\_DC\_UP |  | 18 | 0.19 | 0.78 | 0.732 | 1.000 | 1.000 | 717 | tags=67%, list=51%, signal=134% |
| 879 | GSE11961\_MEMORY\_BCELL\_DAY7\_VS\_PLASMA\_CELL\_DAY7\_UP |  | 17 | 0.19 | 0.78 | 0.720 | 1.000 | 1.000 | 756 | tags=71%, list=54%, signal=150% |
| 880 | GSE17721\_POLYIC\_VS\_GARDIQUIMOD\_4H\_BMDC\_DN |  | 20 | 0.19 | 0.78 | 0.735 | 1.000 | 1.000 | 311 | tags=30%, list=22%, signal=38% |
| 881 | GSE17974\_1.5H\_VS\_72H\_IL4\_AND\_ANTI\_IL12\_ACT\_CD4\_TCELL\_UP |  | 26 | 0.17 | 0.78 | 0.742 | 1.000 | 1.000 | 583 | tags=58%, list=41%, signal=97% |
| 882 | GSE23114\_PERITONEAL\_CAVITY\_B1A\_BCELL\_VS\_SPLEEN\_BCELL\_IN\_SLE2C1\_MOUSE\_DN |  | 16 | 0.20 | 0.78 | 0.759 | 1.000 | 1.000 | 483 | tags=44%, list=34%, signal=66% |
| 883 | GSE3920\_UNTREATED\_VS\_IFNA\_TREATED\_ENDOTHELIAL\_CELL\_DN |  | 16 | 0.20 | 0.78 | 0.739 | 1.000 | 1.000 | 73 | tags=13%, list=5%, signal=13% |
| 884 | GSE22443\_IL2\_VS\_IL12\_TREATED\_ACT\_CD8\_TCELL\_UP |  | 15 | 0.20 | 0.78 | 0.719 | 1.000 | 1.000 | 370 | tags=40%, list=26%, signal=54% |
| 885 | GSE27786\_LIN\_NEG\_VS\_NKCELL\_UP |  | 21 | 0.18 | 0.78 | 0.727 | 1.000 | 1.000 | 334 | tags=33%, list=24%, signal=43% |
| 886 | GSE20727\_CTRL\_VS\_H2O2\_TREATED\_DC\_UP |  | 23 | 0.18 | 0.78 | 0.740 | 1.000 | 1.000 | 198 | tags=22%, list=14%, signal=25% |
| 887 | GSE25088\_CTRL\_VS\_ROSIGLITAZONE\_STIM\_MACROPHAGE\_DN |  | 16 | 0.20 | 0.78 | 0.739 | 1.000 | 1.000 | 31 | tags=13%, list=2%, signal=13% |
| 888 | GSE15659\_CD45RA\_NEG\_CD4\_TCELL\_VS\_ACTIVATED\_TREG\_DN |  | 16 | 0.20 | 0.78 | 0.712 | 1.000 | 1.000 | 607 | tags=56%, list=43%, signal=98% |
| 889 | GSE27786\_CD4\_TCELL\_VS\_NEUTROPHIL\_DN |  | 15 | 0.20 | 0.78 | 0.758 | 1.000 | 1.000 | 131 | tags=20%, list=9%, signal=22% |
| 890 | GSE3982\_NEUTROPHIL\_VS\_EFF\_MEMORY\_CD4\_TCELL\_DN |  | 16 | 0.21 | 0.78 | 0.727 | 1.000 | 1.000 | 388 | tags=38%, list=27%, signal=51% |
| 891 | GSE8921\_UNSTIM\_VS\_TLR1\_2\_STIM\_MONOCYTE\_12H\_UP |  | 17 | 0.19 | 0.78 | 0.724 | 1.000 | 1.000 | 46 | tags=12%, list=3%, signal=12% |
| 892 | GSE2770\_TGFB\_AND\_IL4\_VS\_TGFB\_AND\_IL12\_TREATED\_ACT\_CD4\_TCELL\_48H\_UP |  | 21 | 0.18 | 0.78 | 0.763 | 1.000 | 1.000 | 752 | tags=67%, list=53%, signal=141% |
| 893 | GSE19401\_UNSTIM\_VS\_RETINOIC\_ACID\_STIM\_FOLLICULAR\_DC\_UP |  | 22 | 0.18 | 0.78 | 0.752 | 1.000 | 1.000 | 26 | tags=9%, list=2%, signal=9% |
| 894 | GSE2770\_TGFB\_AND\_IL4\_VS\_TGFB\_AND\_IL12\_TREATED\_ACT\_CD4\_TCELL\_6H\_UP |  | 19 | 0.19 | 0.78 | 0.735 | 1.000 | 1.000 | 22 | tags=11%, list=2%, signal=11% |
| 895 | GSE41176\_WT\_VS\_TAK1\_KO\_ANTI\_IGM\_STIM\_BCELL\_3H\_DN |  | 24 | 0.17 | 0.78 | 0.751 | 1.000 | 1.000 | 69 | tags=13%, list=5%, signal=13% |
| 896 | GSE15659\_RESTING\_VS\_ACTIVATED\_TREG\_DN |  | 16 | 0.20 | 0.78 | 0.754 | 1.000 | 1.000 | 398 | tags=38%, list=28%, signal=52% |
| 897 | GSE37605\_TREG\_VS\_TCONV\_NOD\_FOXP3\_FUSION\_GFP\_DN |  | 22 | 0.17 | 0.77 | 0.759 | 1.000 | 1.000 | 487 | tags=41%, list=35%, signal=61% |
| 898 | GSE43955\_1H\_VS\_20H\_ACT\_CD4\_TCELL\_WITH\_TGFB\_IL6\_UP |  | 15 | 0.20 | 0.77 | 0.752 | 1.000 | 1.000 | 478 | tags=47%, list=34%, signal=70% |
| 899 | GSE17721\_LPS\_VS\_GARDIQUIMOD\_6H\_BMDC\_DN |  | 15 | 0.20 | 0.77 | 0.739 | 1.000 | 1.000 | 57 | tags=13%, list=4%, signal=14% |
| 900 | GSE3982\_EFF\_MEMORY\_CD4\_TCELL\_VS\_TH1\_UP |  | 18 | 0.19 | 0.77 | 0.756 | 1.000 | 1.000 | 366 | tags=33%, list=26%, signal=44% |
| 901 | GSE360\_L\_DONOVANI\_VS\_B\_MALAYI\_HIGH\_DOSE\_DC\_DN |  | 23 | 0.17 | 0.77 | 0.752 | 1.000 | 1.000 | 327 | tags=30%, list=23%, signal=39% |
| 902 | GSE22886\_NAIVE\_BCELL\_VS\_BLOOD\_PLASMA\_CELL\_DN |  | 23 | 0.18 | 0.77 | 0.774 | 1.000 | 1.000 | 93 | tags=13%, list=7%, signal=14% |
| 903 | GSE46606\_IRF4MID\_VS\_WT\_CD40L\_IL2\_IL5\_DAY1\_STIMULATED\_BCELL\_UP |  | 23 | 0.17 | 0.77 | 0.736 | 1.000 | 1.000 | 406 | tags=39%, list=29%, signal=54% |
| 904 | GSE13484\_12H\_UNSTIM\_VS\_YF17D\_VACCINE\_STIM\_PBMC\_UP |  | 26 | 0.17 | 0.77 | 0.750 | 1.000 | 1.000 | 450 | tags=42%, list=32%, signal=61% |
| 905 | GSE4984\_UNTREATED\_VS\_VEHICLE\_CTRL\_TREATED\_DC\_UP |  | 18 | 0.19 | 0.77 | 0.758 | 1.000 | 1.000 | 174 | tags=22%, list=12%, signal=25% |
| 906 | GSE2770\_TGFB\_AND\_IL4\_VS\_TGFB\_AND\_IL12\_TREATED\_ACT\_CD4\_TCELL\_48H\_DN |  | 17 | 0.20 | 0.77 | 0.754 | 1.000 | 1.000 | 364 | tags=41%, list=26%, signal=55% |
| 907 | GSE9037\_CTRL\_VS\_LPS\_4H\_STIM\_BMDM\_DN |  | 24 | 0.17 | 0.77 | 0.754 | 1.000 | 1.000 | 356 | tags=38%, list=25%, signal=49% |
| 908 | GSE6674\_ANTI\_IGM\_VS\_PL2\_3\_STIM\_BCELL\_DN |  | 21 | 0.18 | 0.77 | 0.742 | 1.000 | 1.000 | 223 | tags=24%, list=16%, signal=28% |
| 909 | GSE10463\_CD40L\_AND\_VA347\_VS\_CD40L\_IN\_DC\_UP |  | 26 | 0.17 | 0.77 | 0.784 | 1.000 | 1.000 | 219 | tags=23%, list=16%, signal=27% |
| 910 | GSE17721\_CTRL\_VS\_POLYIC\_24H\_BMDC\_DN |  | 17 | 0.19 | 0.77 | 0.759 | 1.000 | 1.000 | 508 | tags=47%, list=36%, signal=73% |
| 911 | GSE45881\_CXCR6HI\_VS\_CXCR1LO\_COLONIC\_LAMINA\_PROPRIA\_DN |  | 15 | 0.20 | 0.77 | 0.748 | 1.000 | 1.000 | 160 | tags=20%, list=11%, signal=22% |
| 912 | GSE17301\_ACD3\_ACD28\_VS\_ACD3\_ACD28\_AND\_IFNA5\_STIM\_CD8\_TCELL\_UP |  | 19 | 0.19 | 0.77 | 0.765 | 1.000 | 1.000 | 127 | tags=16%, list=9%, signal=17% |
| 913 | GSE15659\_NONSUPPRESSIVE\_TCELL\_VS\_ACTIVATED\_TREG\_DN |  | 16 | 0.20 | 0.77 | 0.741 | 1.000 | 1.000 | 607 | tags=56%, list=43%, signal=98% |
| 914 | GSE2706\_R848\_VS\_LPS\_8H\_STIM\_DC\_DN |  | 17 | 0.19 | 0.76 | 0.749 | 1.000 | 1.000 | 113 | tags=18%, list=8%, signal=19% |
| 915 | GSE41867\_DAY8\_VS\_DAY15\_LCMV\_CLONE13\_EFFECTOR\_CD8\_TCELL\_DN |  | 25 | 0.16 | 0.76 | 0.731 | 1.000 | 1.000 | 743 | tags=68%, list=53%, signal=141% |
| 916 | GSE36826\_NORMAL\_VS\_STAPH\_AUREUS\_INF\_IL1R\_KO\_SKIN\_DN |  | 25 | 0.17 | 0.76 | 0.759 | 1.000 | 1.000 | 484 | tags=44%, list=34%, signal=66% |
| 917 | GSE26669\_CTRL\_VS\_COSTIM\_BLOCK\_MLR\_CD4\_TCELL\_UP |  | 25 | 0.16 | 0.76 | 0.762 | 1.000 | 1.000 | 841 | tags=76%, list=60%, signal=185% |
| 918 | GSE17974\_0.5H\_VS\_72H\_IL4\_AND\_ANTI\_IL12\_ACT\_CD4\_TCELL\_UP |  | 27 | 0.16 | 0.76 | 0.754 | 1.000 | 1.000 | 723 | tags=70%, list=51%, signal=142% |
| 919 | GSE24634\_TREG\_VS\_TCONV\_POST\_DAY3\_IL4\_CONVERSION\_UP |  | 23 | 0.17 | 0.76 | 0.775 | 1.000 | 1.000 | 890 | tags=87%, list=63%, signal=232% |
| 920 | GSE1112\_OT1\_VS\_HY\_CD8AB\_THYMOCYTE\_RTOC\_CULTURE\_DN |  | 26 | 0.16 | 0.76 | 0.756 | 1.000 | 1.000 | 135 | tags=15%, list=10%, signal=17% |
| 921 | GSE23308\_WT\_VS\_MINERALCORTICOID\_REC\_KO\_MACROPHAGE\_CORTICOSTERONE\_TREATED\_UP |  | 21 | 0.18 | 0.76 | 0.762 | 1.000 | 1.000 | 212 | tags=24%, list=15%, signal=28% |
| 922 | GSE17186\_MEMORY\_VS\_NAIVE\_BCELL\_UP |  | 20 | 0.18 | 0.76 | 0.746 | 1.000 | 1.000 | 1071 | tags=95%, list=76%, signal=389% |
| 923 | GSE23925\_DARK\_ZONE\_VS\_NAIVE\_BCELL\_DN |  | 23 | 0.17 | 0.76 | 0.779 | 1.000 | 1.000 | 76 | tags=13%, list=5%, signal=14% |
| 924 | GSE15930\_STIM\_VS\_STIM\_AND\_TRICHOSTATINA\_72H\_CD8\_T\_CELL\_DN |  | 20 | 0.18 | 0.76 | 0.765 | 1.000 | 1.000 | 25 | tags=10%, list=2%, signal=10% |
| 925 | GSE5589\_UNSTIM\_VS\_45MIN\_LPS\_STIM\_MACROPHAGE\_DN |  | 15 | 0.20 | 0.76 | 0.768 | 1.000 | 1.000 | 279 | tags=27%, list=20%, signal=33% |
| 926 | GSE3982\_CENT\_MEMORY\_CD4\_TCELL\_VS\_TH1\_UP |  | 22 | 0.17 | 0.76 | 0.752 | 1.000 | 1.000 | 241 | tags=23%, list=17%, signal=27% |
| 927 | GSE7768\_OVA\_WITH\_LPS\_VS\_OVA\_WITH\_MPL\_IMMUNIZED\_MOUSE\_WHOLE\_SPLEEN\_6H\_UP |  | 18 | 0.19 | 0.76 | 0.768 | 1.000 | 1.000 | 570 | tags=50%, list=40%, signal=83% |
| 928 | GSE411\_100MIN\_VS\_400MIN\_IL6\_STIM\_SOCS3\_KO\_MACROPHAGE\_DN |  | 16 | 0.20 | 0.76 | 0.779 | 1.000 | 1.000 | 162 | tags=19%, list=11%, signal=21% |
| 929 | GSE19888\_ADENOSINE\_A3R\_INH\_VS\_INH\_PRETREAT\_AND\_ACT\_WITH\_TCELL\_MEMBRANES\_MAST\_CELL\_UP |  | 16 | 0.19 | 0.76 | 0.756 | 1.000 | 1.000 | 287 | tags=31%, list=20%, signal=39% |
| 930 | GSE35543\_IN\_VIVO\_NTREG\_VS\_IN\_VITRO\_ITREG\_DN |  | 16 | 0.20 | 0.76 | 0.765 | 1.000 | 1.000 | 45 | tags=13%, list=3%, signal=13% |
| 931 | GSE13946\_CTRL\_VS\_DSS\_COLITIS\_GD\_TCELL\_FROM\_COLON\_DN |  | 23 | 0.17 | 0.76 | 0.789 | 1.000 | 1.000 | 344 | tags=30%, list=24%, signal=40% |
| 932 | GSE36476\_CTRL\_VS\_TSST\_ACT\_72H\_MEMORY\_CD4\_TCELL\_OLD\_DN |  | 20 | 0.18 | 0.76 | 0.770 | 1.000 | 1.000 | 777 | tags=70%, list=55%, signal=154% |
| 933 | GSE26928\_EFF\_MEM\_VS\_CENTR\_MEM\_CD4\_TCELL\_UP |  | 17 | 0.19 | 0.76 | 0.751 | 1.000 | 1.000 | 266 | tags=29%, list=19%, signal=36% |
| 934 | GSE3691\_IFN\_PRODUCING\_KILLER\_DC\_VS\_PLASMACYTOID\_DC\_SPLEEN\_DN |  | 17 | 0.19 | 0.76 | 0.765 | 1.000 | 1.000 | 261 | tags=29%, list=18%, signal=36% |
| 935 | GSE25088\_CTRL\_VS\_IL4\_STIM\_MACROPHAGE\_UP |  | 23 | 0.17 | 0.76 | 0.773 | 1.000 | 1.000 | 459 | tags=43%, list=33%, signal=63% |
| 936 | GSE11961\_MARGINAL\_ZONE\_BCELL\_VS\_GERMINAL\_CENTER\_BCELL\_DAY7\_DN |  | 19 | 0.18 | 0.76 | 0.769 | 1.000 | 1.000 | 30 | tags=11%, list=2%, signal=11% |
| 937 | GSE24210\_CTRL\_VS\_IL35\_TREATED\_TCONV\_CD4\_TCELL\_DN |  | 22 | 0.17 | 0.76 | 0.799 | 1.000 | 1.000 | 825 | tags=77%, list=58%, signal=183% |
| 938 | GSE15767\_MED\_VS\_SCS\_MAC\_LN\_DN |  | 20 | 0.18 | 0.76 | 0.756 | 1.000 | 1.000 | 657 | tags=60%, list=47%, signal=111% |
| 939 | GSE1460\_INTRATHYMIC\_T\_PROGENITOR\_VS\_NAIVE\_CD4\_TCELL\_CORD\_BLOOD\_UP |  | 19 | 0.18 | 0.75 | 0.777 | 1.000 | 1.000 | 492 | tags=42%, list=35%, signal=64% |
| 940 | GSE1460\_DP\_THYMOCYTE\_VS\_THYMIC\_STROMAL\_CELL\_DN |  | 17 | 0.19 | 0.75 | 0.767 | 1.000 | 1.000 | 269 | tags=24%, list=19%, signal=29% |
| 941 | GSE6269\_HEALTHY\_VS\_STAPH\_AUREUS\_INF\_PBMC\_DN |  | 16 | 0.19 | 0.75 | 0.779 | 1.000 | 1.000 | 38 | tags=13%, list=3%, signal=13% |
| 942 | GSE21927\_BALBC\_VS\_C57BL6\_MONOCYTE\_SPLEEN\_UP |  | 20 | 0.18 | 0.75 | 0.757 | 1.000 | 1.000 | 566 | tags=55%, list=40%, signal=91% |
| 943 | GSE3982\_BASOPHIL\_VS\_CENT\_MEMORY\_CD4\_TCELL\_DN |  | 18 | 0.18 | 0.75 | 0.769 | 1.000 | 1.000 | 664 | tags=67%, list=47%, signal=124% |
| 944 | GSE8621\_LPS\_PRIMED\_UNSTIM\_VS\_LPS\_PRIMED\_AND\_LPS\_STIM\_MACROPHAGE\_UP |  | 18 | 0.19 | 0.75 | 0.779 | 1.000 | 1.000 | 128 | tags=17%, list=9%, signal=18% |
| 945 | GSE12392\_CD8A\_POS\_VS\_NEG\_SPLEEN\_DC\_UP |  | 19 | 0.18 | 0.75 | 0.766 | 1.000 | 1.000 | 556 | tags=53%, list=39%, signal=86% |
| 946 | GSE25085\_FETAL\_LIVER\_VS\_FETAL\_BM\_SP4\_THYMIC\_IMPLANT\_UP |  | 17 | 0.19 | 0.75 | 0.790 | 1.000 | 1.000 | 595 | tags=53%, list=42%, signal=90% |
| 947 | GSE17580\_TREG\_VS\_TEFF\_UP |  | 19 | 0.18 | 0.75 | 0.767 | 1.000 | 1.000 | 326 | tags=32%, list=23%, signal=41% |
| 948 | GSE16755\_CTRL\_VS\_IFNA\_TREATED\_MAC\_UP |  | 19 | 0.18 | 0.75 | 0.790 | 1.000 | 1.000 | 439 | tags=42%, list=31%, signal=60% |
| 949 | GSE10273\_HIGH\_IL7\_VS\_HIGH\_IL7\_AND\_IRF4\_IN\_IRF4\_8\_NULL\_PRE\_BCELL\_UP |  | 17 | 0.19 | 0.75 | 0.783 | 1.000 | 1.000 | 890 | tags=88%, list=63%, signal=236% |
| 950 | GSE36891\_UNSTIM\_VS\_POLYIC\_TLR3\_STIM\_PERITONEAL\_MACROPHAGE\_DN |  | 22 | 0.17 | 0.75 | 0.769 | 1.000 | 1.000 | 282 | tags=27%, list=20%, signal=34% |
| 951 | GSE13229\_IMM\_VS\_INTMATURE\_NKCELL\_DN |  | 22 | 0.17 | 0.75 | 0.785 | 1.000 | 1.000 | 16 | tags=9%, list=1%, signal=9% |
| 952 | GSE3982\_DC\_VS\_EFF\_MEMORY\_CD4\_TCELL\_UP |  | 27 | 0.16 | 0.75 | 0.772 | 1.000 | 1.000 | 255 | tags=26%, list=18%, signal=31% |
| 953 | GSE43955\_TH0\_VS\_TGFB\_IL6\_IL23\_TH17\_ACT\_CD4\_TCELL\_52H\_UP |  | 21 | 0.17 | 0.75 | 0.779 | 1.000 | 1.000 | 1168 | tags=100%, list=83%, signal=572% |
| 954 | GSE9037\_CTRL\_VS\_LPS\_1H\_STIM\_IRAK4\_KO\_BMDM\_UP |  | 17 | 0.18 | 0.75 | 0.753 | 1.000 | 1.000 | 717 | tags=71%, list=51%, signal=142% |
| 955 | GSE11864\_UNTREATED\_VS\_CSF1\_IFNG\_PAM3CYS\_IN\_MAC\_UP |  | 15 | 0.19 | 0.75 | 0.790 | 1.000 | 1.000 | 245 | tags=27%, list=17%, signal=32% |
| 956 | GSE7460\_CTRL\_VS\_TGFB\_TREATED\_ACT\_TREG\_UP |  | 27 | 0.16 | 0.75 | 0.777 | 1.000 | 1.000 | 224 | tags=22%, list=16%, signal=26% |
| 957 | GSE6259\_FLT3L\_INDUCED\_33D1\_POS\_DC\_VS\_CD4\_TCELL\_DN |  | 18 | 0.18 | 0.75 | 0.761 | 1.000 | 1.000 | 138 | tags=17%, list=10%, signal=18% |
| 958 | GSE9878\_CTRL\_VS\_EBF\_TRANSDUCED\_PAX5\_KO\_PRO\_BCELL\_UP |  | 18 | 0.19 | 0.75 | 0.760 | 1.000 | 1.000 | 92 | tags=17%, list=7%, signal=18% |
| 959 | GSE411\_100MIN\_VS\_400MIN\_IL6\_STIM\_MACROPHAGE\_DN |  | 31 | 0.15 | 0.74 | 0.785 | 1.000 | 1.000 | 144 | tags=16%, list=10%, signal=18% |
| 960 | GSE9946\_MATURE\_STIMULATORY\_VS\_LISTERIA\_INF\_MATURE\_DC\_UP |  | 20 | 0.17 | 0.74 | 0.754 | 1.000 | 1.000 | 181 | tags=20%, list=13%, signal=23% |
| 961 | GSE21063\_CTRL\_VS\_ANTI\_IGM\_STIM\_BCELL\_16H\_UP |  | 15 | 0.20 | 0.74 | 0.760 | 1.000 | 1.000 | 234 | tags=27%, list=17%, signal=32% |
| 962 | GSE37301\_COMMON\_LYMPHOID\_PROGENITOR\_VS\_RAG2\_KO\_NK\_CELL\_DN |  | 16 | 0.19 | 0.74 | 0.802 | 1.000 | 1.000 | 103 | tags=19%, list=7%, signal=20% |
| 963 | GSE27786\_CD4\_TCELL\_VS\_NKCELL\_UP |  | 19 | 0.18 | 0.74 | 0.785 | 1.000 | 1.000 | 1162 | tags=100%, list=82%, signal=559% |
| 964 | GSE37416\_0H\_VS\_6H\_F\_TULARENSIS\_LVS\_NEUTROPHIL\_UP |  | 22 | 0.17 | 0.74 | 0.797 | 1.000 | 1.000 | 473 | tags=45%, list=34%, signal=67% |
| 965 | GSE12845\_IGD\_POS\_BLOOD\_VS\_NAIVE\_TONSIL\_BCELL\_UP |  | 21 | 0.17 | 0.74 | 0.781 | 1.000 | 1.000 | 315 | tags=29%, list=22%, signal=36% |
| 966 | GSE21927\_GMCSF\_IL6\_VS\_GMCSF\_GCSF\_TREATED\_BONE\_MARROW\_DN |  | 24 | 0.17 | 0.74 | 0.787 | 1.000 | 1.000 | 704 | tags=63%, list=50%, signal=123% |
| 967 | GSE29618\_PRE\_VS\_DAY7\_POST\_LAIV\_FLU\_VACCINE\_MDC\_DN |  | 18 | 0.18 | 0.74 | 0.800 | 1.000 | 1.000 | 175 | tags=22%, list=12%, signal=25% |
| 968 | GSE7548\_NAIVE\_VS\_DAY7\_PCC\_IMMUNIZATION\_CD4\_TCELL\_UP |  | 26 | 0.16 | 0.74 | 0.802 | 1.000 | 1.000 | 701 | tags=62%, list=50%, signal=120% |
| 969 | GSE22886\_IGM\_MEMORY\_BCELL\_VS\_BM\_PLASMA\_CELL\_UP |  | 20 | 0.17 | 0.74 | 0.785 | 1.000 | 1.000 | 449 | tags=45%, list=32%, signal=65% |
| 970 | GSE14000\_4H\_VS\_16H\_LPS\_DC\_TRANSLATED\_RNA\_DN |  | 22 | 0.17 | 0.74 | 0.814 | 1.000 | 1.000 | 1028 | tags=95%, list=73%, signal=346% |
| 971 | GSE17721\_LPS\_VS\_PAM3CSK4\_1H\_BMDC\_UP |  | 15 | 0.20 | 0.73 | 0.801 | 1.000 | 1.000 | 451 | tags=40%, list=32%, signal=58% |
| 972 | GSE16385\_IL4\_VS\_ROSIGLITAZONE\_STIM\_MACROPHAGE\_UP |  | 24 | 0.16 | 0.73 | 0.800 | 1.000 | 1.000 | 196 | tags=21%, list=14%, signal=24% |
| 973 | GSE43955\_TH0\_VS\_TGFB\_IL6\_TH17\_ACT\_CD4\_TCELL\_1H\_DN |  | 15 | 0.20 | 0.73 | 0.792 | 1.000 | 1.000 | 492 | tags=47%, list=35%, signal=71% |
| 974 | GSE40666\_WT\_VS\_STAT4\_KO\_CD8\_TCELL\_DN |  | 23 | 0.16 | 0.73 | 0.802 | 1.000 | 1.000 | 317 | tags=30%, list=22%, signal=39% |
| 975 | GSE39022\_LN\_VS\_SPLEEN\_DC\_DN |  | 23 | 0.16 | 0.73 | 0.808 | 1.000 | 1.000 | 16 | tags=9%, list=1%, signal=9% |
| 976 | GSE8515\_IL1\_VS\_IL6\_4H\_STIM\_MAC\_UP |  | 24 | 0.16 | 0.73 | 0.804 | 1.000 | 1.000 | 499 | tags=46%, list=35%, signal=70% |
| 977 | GSE45365\_HEALTHY\_VS\_MCMV\_INFECTION\_CD11B\_DC\_DN |  | 17 | 0.18 | 0.73 | 0.791 | 1.000 | 1.000 | 66 | tags=12%, list=5%, signal=12% |
| 978 | GSE21670\_STAT3\_KO\_VS\_WT\_CD4\_TCELL\_IL6\_TREATED\_DN |  | 23 | 0.16 | 0.73 | 0.809 | 1.000 | 1.000 | 158 | tags=17%, list=11%, signal=19% |
| 979 | GSE360\_T\_GONDII\_VS\_B\_MALAYI\_LOW\_DOSE\_MAC\_UP |  | 16 | 0.19 | 0.73 | 0.795 | 1.000 | 1.000 | 1019 | tags=94%, list=72%, signal=334% |
| 980 | GSE6090\_UNSTIM\_VS\_DC\_SIGN\_STIM\_DC\_UP |  | 19 | 0.18 | 0.73 | 0.796 | 1.000 | 1.000 | 650 | tags=63%, list=46%, signal=116% |
| 981 | GSE36392\_EOSINOPHIL\_VS\_MAC\_IL25\_TREATED\_LUNG\_DN |  | 21 | 0.17 | 0.73 | 0.781 | 1.000 | 1.000 | 570 | tags=57%, list=40%, signal=94% |
| 982 | GSE23925\_LIGHT\_ZONE\_VS\_DARK\_ZONE\_BCELL\_DN |  | 19 | 0.17 | 0.73 | 0.815 | 1.000 | 1.000 | 394 | tags=37%, list=28%, signal=50% |
| 983 | GSE30971\_WBP7\_HET\_VS\_KO\_MACROPHAGE\_4H\_LPS\_STIM\_UP |  | 28 | 0.15 | 0.73 | 0.827 | 1.000 | 1.000 | 497 | tags=46%, list=35%, signal=70% |
| 984 | GSE22886\_NAIVE\_CD8\_TCELL\_VS\_DC\_DN |  | 19 | 0.18 | 0.73 | 0.785 | 1.000 | 1.000 | 327 | tags=32%, list=23%, signal=41% |
| 985 | GSE21546\_WT\_VS\_ELK1\_KO\_DP\_THYMOCYTES\_UP |  | 18 | 0.18 | 0.73 | 0.792 | 1.000 | 1.000 | 378 | tags=39%, list=27%, signal=52% |
| 986 | GSE10147\_IL3\_VS\_IL3\_AND\_HIVP17\_STIM\_PDC\_UP |  | 22 | 0.17 | 0.73 | 0.798 | 1.000 | 1.000 | 73 | tags=14%, list=5%, signal=14% |
| 987 | GSE25123\_ROSIGLITAZONE\_VS\_IL4\_AND\_ROSIGLITAZONE\_STIM\_PPARG\_KO\_MACROPHAGE\_DAY10\_UP |  | 22 | 0.17 | 0.73 | 0.790 | 1.000 | 1.000 | 951 | tags=86%, list=67%, signal=261% |
| 988 | GSE27291\_6H\_VS\_7D\_STIM\_GAMMADELTA\_TCELL\_UP |  | 20 | 0.17 | 0.73 | 0.810 | 1.000 | 1.000 | 357 | tags=30%, list=25%, signal=40% |
| 989 | GSE17721\_CTRL\_VS\_CPG\_0.5H\_BMDC\_UP |  | 18 | 0.18 | 0.73 | 0.787 | 1.000 | 1.000 | 473 | tags=44%, list=34%, signal=66% |
| 990 | GSE15750\_WT\_VS\_TRAF6KO\_DAY10\_EFF\_CD8\_TCELL\_UP |  | 17 | 0.18 | 0.73 | 0.803 | 1.000 | 1.000 | 582 | tags=59%, list=41%, signal=99% |
| 991 | GSE24726\_WT\_VS\_E2\_22\_KO\_PDC\_DAY4\_POST\_DELETION\_DN |  | 22 | 0.16 | 0.73 | 0.779 | 1.000 | 1.000 | 23 | tags=9%, list=2%, signal=9% |
| 992 | GSE11961\_MEMORY\_BCELL\_DAY7\_VS\_PLASMA\_CELL\_DAY7\_DN |  | 18 | 0.18 | 0.73 | 0.796 | 1.000 | 1.000 | 324 | tags=33%, list=23%, signal=43% |
| 993 | GSE41867\_LCMV\_ARMSTRONG\_VS\_CLONE13\_DAY15\_EFFECTOR\_CD8\_TCELL\_UP |  | 17 | 0.18 | 0.73 | 0.775 | 1.000 | 1.000 | 258 | tags=29%, list=18%, signal=36% |
| 994 | GSE26928\_NAIVE\_VS\_EFF\_MEMORY\_CD4\_TCELL\_UP |  | 20 | 0.17 | 0.73 | 0.807 | 1.000 | 1.000 | 710 | tags=65%, list=50%, signal=129% |
| 995 | GSE14308\_TH1\_VS\_INDUCED\_TREG\_UP |  | 16 | 0.18 | 0.73 | 0.798 | 1.000 | 1.000 | 264 | tags=25%, list=19%, signal=30% |
| 996 | GSE41867\_DAY15\_EFFECTOR\_VS\_DAY30\_EXHAUSTED\_CD8\_TCELL\_LCMV\_CLONE13\_DN |  | 23 | 0.17 | 0.73 | 0.816 | 1.000 | 1.000 | 224 | tags=22%, list=16%, signal=25% |
| 997 | GSE7768\_OVA\_ALONE\_VS\_OVA\_WITH\_LPS\_IMMUNIZED\_MOUSE\_WHOLE\_SPLEEN\_6H\_UP |  | 17 | 0.18 | 0.73 | 0.814 | 1.000 | 1.000 | 187 | tags=24%, list=13%, signal=27% |
| 998 | GSE17721\_POLYIC\_VS\_GARDIQUIMOD\_1H\_BMDC\_UP |  | 16 | 0.19 | 0.73 | 0.826 | 1.000 | 1.000 | 535 | tags=56%, list=38%, signal=90% |
| 999 | GSE31082\_DP\_VS\_CD4\_SP\_THYMOCYTE\_DN |  | 22 | 0.17 | 0.73 | 0.807 | 1.000 | 1.000 | 415 | tags=36%, list=29%, signal=51% |
| 1000 | GSE26351\_UNSTIM\_VS\_WNT\_PATHWAY\_STIM\_HEMATOPOIETIC\_PROGENITORS\_DN |  | 28 | 0.15 | 0.72 | 0.807 | 1.000 | 1.000 | 305 | tags=29%, list=22%, signal=36% |
| 1001 | GSE4748\_CTRL\_VS\_CYANOBACTERIUM\_LPSLIKE\_STIM\_DC\_1H\_UP |  | 20 | 0.17 | 0.72 | 0.785 | 1.000 | 1.000 | 42 | tags=10%, list=3%, signal=10% |
| 1002 | GSE17721\_12H\_VS\_24H\_CPG\_BMDC\_DN |  | 23 | 0.16 | 0.72 | 0.812 | 1.000 | 1.000 | 734 | tags=70%, list=52%, signal=143% |
| 1003 | GSE24726\_WT\_VS\_E2\_2\_KO\_PDC\_DAY4\_POST\_DELETION\_UP |  | 18 | 0.18 | 0.72 | 0.813 | 1.000 | 1.000 | 27 | tags=11%, list=2%, signal=11% |
| 1004 | GSE14308\_TH1\_VS\_TH17\_DN |  | 15 | 0.19 | 0.72 | 0.805 | 1.000 | 1.000 | 403 | tags=40%, list=29%, signal=55% |
| 1005 | GSE8515\_CTRL\_VS\_IL1\_4H\_STIM\_MAC\_UP |  | 22 | 0.17 | 0.72 | 0.816 | 1.000 | 1.000 | 1100 | tags=95%, list=78%, signal=426% |
| 1006 | GSE27786\_LIN\_NEG\_VS\_NKTCELL\_DN |  | 17 | 0.18 | 0.72 | 0.804 | 1.000 | 1.000 | 1160 | tags=100%, list=82%, signal=555% |
| 1007 | GSE22886\_NEUTROPHIL\_VS\_MONOCYTE\_DN |  | 18 | 0.18 | 0.72 | 0.823 | 1.000 | 1.000 | 557 | tags=50%, list=39%, signal=82% |
| 1008 | GSE26343\_WT\_VS\_NFAT5\_KO\_MACROPHAGE\_LPS\_STIM\_DN |  | 25 | 0.15 | 0.72 | 0.793 | 1.000 | 1.000 | 274 | tags=28%, list=19%, signal=34% |
| 1009 | GSE19888\_ADENOSINE\_A3R\_ACT\_VS\_A3R\_ACT\_WITH\_A3R\_INH\_PRETREATMENT\_IN\_MAST\_CELL\_DN |  | 24 | 0.16 | 0.72 | 0.819 | 1.000 | 1.000 | 208 | tags=21%, list=15%, signal=24% |
| 1010 | GSE41867\_DAY6\_VS\_DAY8\_LCMV\_ARMSTRONG\_EFFECTOR\_CD8\_TCELL\_DN |  | 24 | 0.16 | 0.72 | 0.823 | 1.000 | 1.000 | 876 | tags=83%, list=62%, signal=216% |
| 1011 | GSE44649\_NAIVE\_VS\_ACTIVATED\_CD8\_TCELL\_UP |  | 19 | 0.17 | 0.72 | 0.780 | 1.000 | 1.000 | 998 | tags=89%, list=71%, signal=302% |
| 1012 | GSE3982\_NEUTROPHIL\_VS\_CENT\_MEMORY\_CD4\_TCELL\_DN |  | 21 | 0.17 | 0.72 | 0.813 | 1.000 | 1.000 | 1179 | tags=100%, list=84%, signal=599% |
| 1013 | GSE19825\_CD24LOW\_VS\_IL2RA\_HIGH\_DAY3\_EFF\_CD8\_TCELL\_DN |  | 30 | 0.15 | 0.72 | 0.809 | 1.000 | 1.000 | 490 | tags=43%, list=35%, signal=65% |
| 1014 | GSE27786\_CD8\_TCELL\_VS\_ERYTHROBLAST\_DN |  | 19 | 0.17 | 0.72 | 0.810 | 1.000 | 1.000 | 668 | tags=63%, list=47%, signal=118% |
| 1015 | GSE27786\_NKCELL\_VS\_MONO\_MAC\_DN |  | 19 | 0.17 | 0.72 | 0.823 | 1.000 | 1.000 | 44 | tags=11%, list=3%, signal=11% |
| 1016 | GSE13306\_TREG\_RA\_VS\_TCONV\_RA\_UP |  | 20 | 0.17 | 0.71 | 0.812 | 1.000 | 1.000 | 45 | tags=10%, list=3%, signal=10% |
| 1017 | GSE36009\_WT\_VS\_NLRP10\_KO\_DC\_LPS\_STIM\_DN |  | 27 | 0.15 | 0.71 | 0.814 | 1.000 | 1.000 | 763 | tags=70%, list=54%, signal=150% |
| 1018 | GSE18893\_TCONV\_VS\_TREG\_24H\_TNF\_STIM\_DN |  | 22 | 0.16 | 0.71 | 0.824 | 1.000 | 1.000 | 267 | tags=23%, list=19%, signal=28% |
| 1019 | GSE40273\_GATA1\_KO\_VS\_WT\_TREG\_DN |  | 22 | 0.16 | 0.71 | 0.808 | 1.000 | 1.000 | 463 | tags=41%, list=33%, signal=60% |
| 1020 | GSE24634\_NAIVE\_CD4\_TCELL\_VS\_DAY10\_IL4\_CONV\_TREG\_DN |  | 24 | 0.15 | 0.71 | 0.819 | 1.000 | 1.000 | 478 | tags=46%, list=34%, signal=68% |
| 1021 | GSE4748\_CYANOBACTERIUM\_LPSLIKE\_VS\_LPS\_AND\_CYANOBACTERIUM\_LPSLIKE\_STIM\_DC\_3H\_UP |  | 22 | 0.16 | 0.71 | 0.838 | 1.000 | 1.000 | 628 | tags=55%, list=45%, signal=97% |
| 1022 | GSE36476\_YOUNG\_VS\_OLD\_DONOR\_MEMORY\_CD4\_TCELL\_40H\_TSST\_ACT\_DN |  | 19 | 0.17 | 0.71 | 0.826 | 1.000 | 1.000 | 528 | tags=47%, list=37%, signal=75% |
| 1023 | GSE32423\_MEMORY\_VS\_NAIVE\_CD8\_TCELL\_IL7\_UP |  | 20 | 0.16 | 0.71 | 0.794 | 1.000 | 1.000 | 297 | tags=25%, list=21%, signal=31% |
| 1024 | GSE19888\_CTRL\_VS\_A3R\_ACTIVATION\_MAST\_CELL\_UP |  | 22 | 0.16 | 0.71 | 0.810 | 1.000 | 1.000 | 98 | tags=14%, list=7%, signal=14% |
| 1025 | GSE25088\_CTRL\_VS\_ROSIGLITAZONE\_STIM\_MACROPHAGE\_UP |  | 24 | 0.16 | 0.71 | 0.848 | 1.000 | 1.000 | 448 | tags=38%, list=32%, signal=54% |
| 1026 | GSE1432\_6H\_VS\_24H\_IFNG\_MICROGLIA\_DN |  | 18 | 0.17 | 0.71 | 0.816 | 1.000 | 1.000 | 141 | tags=17%, list=10%, signal=18% |
| 1027 | GSE1460\_CD4\_THYMOCYTE\_VS\_THYMIC\_STROMAL\_CELL\_UP |  | 18 | 0.17 | 0.71 | 0.822 | 1.000 | 1.000 | 551 | tags=50%, list=39%, signal=81% |
| 1028 | GSE22886\_TH1\_VS\_TH2\_48H\_ACT\_DN |  | 26 | 0.15 | 0.71 | 0.813 | 1.000 | 1.000 | 33 | tags=8%, list=2%, signal=8% |
| 1029 | GSE20715\_0H\_VS\_6H\_OZONE\_LUNG\_UP |  | 19 | 0.17 | 0.71 | 0.829 | 1.000 | 1.000 | 216 | tags=21%, list=15%, signal=25% |
| 1030 | GSE1925\_CTRL\_VS\_3H\_IFNG\_STIM\_MACROPHAGE\_UP |  | 21 | 0.16 | 0.71 | 0.837 | 1.000 | 1.000 | 414 | tags=33%, list=29%, signal=46% |
| 1031 | GSE23505\_IL6\_IL1\_VS\_IL6\_IL1\_TGFB\_TREATED\_CD4\_TCELL\_UP |  | 17 | 0.18 | 0.71 | 0.830 | 1.000 | 1.000 | 40 | tags=12%, list=3%, signal=12% |
| 1032 | GSE25088\_WT\_VS\_STAT6\_KO\_MACROPHAGE\_IL4\_STIM\_DN |  | 15 | 0.18 | 0.71 | 0.819 | 1.000 | 1.000 | 1 | tags=7%, list=0%, signal=7% |
| 1033 | GSE17721\_0.5H\_VS\_24H\_PAM3CSK4\_BMDC\_UP |  | 17 | 0.18 | 0.71 | 0.830 | 1.000 | 1.000 | 494 | tags=47%, list=35%, signal=72% |
| 1034 | GSE8685\_IL15\_ACT\_IL2\_STARVED\_VS\_IL21\_ACT\_IL2\_STARVED\_CD4\_TCELL\_DN |  | 20 | 0.17 | 0.70 | 0.822 | 1.000 | 1.000 | 887 | tags=85%, list=63%, signal=226% |
| 1035 | GSE22886\_IGM\_MEMORY\_BCELL\_VS\_BLOOD\_PLASMA\_CELL\_DN |  | 16 | 0.18 | 0.70 | 0.816 | 1.000 | 1.000 | 62 | tags=13%, list=4%, signal=13% |
| 1036 | GSE17721\_POLYIC\_VS\_GARDIQUIMOD\_6H\_BMDC\_DN |  | 15 | 0.19 | 0.70 | 0.823 | 1.000 | 1.000 | 166 | tags=20%, list=12%, signal=22% |
| 1037 | GSE22886\_DC\_VS\_MONOCYTE\_UP |  | 24 | 0.16 | 0.70 | 0.836 | 1.000 | 1.000 | 335 | tags=29%, list=24%, signal=38% |
| 1038 | GSE39820\_CTRL\_VS\_TGFBETA1\_IL6\_IL23A\_CD4\_TCELL\_UP |  | 26 | 0.15 | 0.70 | 0.853 | 1.000 | 1.000 | 824 | tags=73%, list=58%, signal=172% |
| 1039 | GSE16385\_ROSIGLITAZONE\_IFNG\_TNF\_VS\_IL4\_STIM\_MACROPHAGE\_DN |  | 18 | 0.17 | 0.70 | 0.834 | 1.000 | 1.000 | 34 | tags=11%, list=2%, signal=11% |
| 1040 | GSE8685\_IL2\_ACT\_IL2\_STARVED\_VS\_IL15\_ACT\_IL2\_STARVED\_CD4\_TCELL\_UP |  | 19 | 0.17 | 0.70 | 0.818 | 1.000 | 1.000 | 30 | tags=11%, list=2%, signal=11% |
| 1041 | GSE15930\_NAIVE\_VS\_48H\_IN\_VITRO\_STIM\_CD8\_TCELL\_DN |  | 26 | 0.15 | 0.70 | 0.853 | 1.000 | 1.000 | 1001 | tags=92%, list=71%, signal=312% |
| 1042 | GSE41176\_UNSTIM\_VS\_ANTI\_IGM\_STIM\_TAK1\_KO\_BCELL\_1H\_UP |  | 31 | 0.14 | 0.70 | 0.849 | 1.000 | 1.000 | 20 | tags=6%, list=1%, signal=6% |
| 1043 | GSE13493\_DP\_VS\_CD8POS\_THYMOCYTE\_UP |  | 16 | 0.18 | 0.70 | 0.831 | 1.000 | 1.000 | 1161 | tags=100%, list=82%, signal=558% |
| 1044 | GSE5099\_MONOCYTE\_VS\_CLASSICAL\_M1\_MACROPHAGE\_DN |  | 23 | 0.15 | 0.70 | 0.830 | 1.000 | 1.000 | 82 | tags=13%, list=6%, signal=14% |
| 1045 | GSE1460\_DP\_THYMOCYTE\_VS\_NAIVE\_CD4\_TCELL\_CORD\_BLOOD\_UP |  | 19 | 0.16 | 0.70 | 0.823 | 1.000 | 1.000 | 138 | tags=16%, list=10%, signal=17% |
| 1046 | GSE13484\_12H\_VS\_3H\_YF17D\_VACCINE\_STIM\_PBMC\_UP |  | 21 | 0.16 | 0.70 | 0.842 | 1.000 | 1.000 | 301 | tags=29%, list=21%, signal=36% |
| 1047 | GSE8678\_IL7R\_LOW\_VS\_HIGH\_EFF\_CD8\_TCELL\_DN |  | 23 | 0.16 | 0.70 | 0.840 | 1.000 | 1.000 | 84 | tags=13%, list=6%, signal=14% |
| 1048 | GSE3039\_CD4\_TCELL\_VS\_ALPHABETA\_CD8\_TCELL\_UP |  | 20 | 0.17 | 0.70 | 0.848 | 1.000 | 1.000 | 384 | tags=35%, list=27%, signal=47% |
| 1049 | GSE3920\_UNTREATED\_VS\_IFNA\_TREATED\_FIBROBLAST\_DN |  | 22 | 0.16 | 0.69 | 0.862 | 1.000 | 1.000 | 647 | tags=55%, list=46%, signal=99% |
| 1050 | GSE37533\_PPARG1\_FOXP3\_VS\_PPARG2\_FOXP3\_TRANSDUCED\_CD4\_TCELL\_DN |  | 25 | 0.15 | 0.69 | 0.841 | 1.000 | 1.000 | 20 | tags=8%, list=1%, signal=8% |
| 1051 | GSE17974\_2H\_VS\_72H\_UNTREATED\_IN\_VITRO\_CD4\_TCELL\_UP |  | 35 | 0.13 | 0.69 | 0.870 | 1.000 | 1.000 | 582 | tags=49%, list=41%, signal=81% |
| 1052 | GSE24634\_NAIVE\_CD4\_TCELL\_VS\_DAY3\_IL4\_CONV\_TREG\_DN |  | 22 | 0.16 | 0.69 | 0.833 | 1.000 | 1.000 | 646 | tags=55%, list=46%, signal=99% |
| 1053 | GSE20198\_IL12\_VS\_IL12\_IL18\_TREATED\_ACT\_CD4\_TCELL\_UP |  | 21 | 0.17 | 0.69 | 0.827 | 1.000 | 1.000 | 510 | tags=43%, list=36%, signal=66% |
| 1054 | GSE1432\_6H\_VS\_24H\_IFNG\_MICROGLIA\_UP |  | 16 | 0.18 | 0.69 | 0.848 | 1.000 | 1.000 | 334 | tags=31%, list=24%, signal=40% |
| 1055 | GSE21546\_ELK1\_KO\_VS\_SAP1A\_KO\_AND\_ELK1\_KO\_ANTI\_CD3\_STIM\_DP\_THYMOCYTES\_DN |  | 35 | 0.14 | 0.69 | 0.853 | 1.000 | 1.000 | 306 | tags=26%, list=22%, signal=32% |
| 1056 | GSE28726\_NAIVE\_VS\_ACTIVATED\_CD4\_TCELL\_DN |  | 19 | 0.17 | 0.69 | 0.825 | 1.000 | 1.000 | 265 | tags=21%, list=19%, signal=26% |
| 1057 | GSE7509\_DC\_VS\_MONOCYTE\_WITH\_FCGRIIB\_STIM\_UP |  | 35 | 0.13 | 0.69 | 0.852 | 1.000 | 1.000 | 576 | tags=49%, list=41%, signal=80% |
| 1058 | GSE3982\_MEMORY\_CD4\_TCELL\_VS\_TH1\_UP |  | 26 | 0.15 | 0.69 | 0.826 | 1.000 | 1.000 | 161 | tags=15%, list=11%, signal=17% |
| 1059 | GSE26351\_WNT\_VS\_BMP\_PATHWAY\_STIM\_HEMATOPOIETIC\_PROGENITORS\_DN |  | 19 | 0.17 | 0.69 | 0.872 | 1.000 | 1.000 | 709 | tags=68%, list=50%, signal=136% |
| 1060 | GSE7348\_LPS\_VS\_TOLERIZED\_AND\_LPS\_STIM\_MACROPHAGE\_DN |  | 20 | 0.16 | 0.69 | 0.851 | 1.000 | 1.000 | 384 | tags=35%, list=27%, signal=47% |
| 1061 | GSE5542\_UNTREATED\_VS\_IFNA\_TREATED\_EPITHELIAL\_CELLS\_6H\_DN |  | 17 | 0.17 | 0.69 | 0.841 | 1.000 | 1.000 | 570 | tags=53%, list=40%, signal=88% |
| 1062 | GSE7831\_1H\_VS\_4H\_CPG\_STIM\_PDC\_UP |  | 19 | 0.17 | 0.69 | 0.835 | 1.000 | 1.000 | 54 | tags=11%, list=4%, signal=11% |
| 1063 | GSE24210\_IL35\_TREATED\_VS\_UNTREATED\_TCONV\_CD4\_TCELL\_UP |  | 23 | 0.16 | 0.69 | 0.854 | 1.000 | 1.000 | 195 | tags=22%, list=14%, signal=25% |
| 1064 | GSE17974\_CTRL\_VS\_ACT\_IL4\_AND\_ANTI\_IL12\_12H\_CD4\_TCELL\_DN |  | 28 | 0.14 | 0.68 | 0.860 | 1.000 | 1.000 | 947 | tags=86%, list=67%, signal=255% |
| 1065 | GSE2770\_UNTREATED\_VS\_TGFB\_AND\_IL12\_TREATED\_ACT\_CD4\_TCELL\_6H\_UP |  | 25 | 0.15 | 0.68 | 0.860 | 1.000 | 1.000 | 20 | tags=8%, list=1%, signal=8% |
| 1066 | GSE11924\_TFH\_VS\_TH1\_CD4\_TCELL\_DN |  | 15 | 0.18 | 0.68 | 0.845 | 1.000 | 1.000 | 736 | tags=73%, list=52%, signal=152% |
| 1067 | GSE3982\_MAC\_VS\_CENT\_MEMORY\_CD4\_TCELL\_DN |  | 16 | 0.18 | 0.68 | 0.822 | 1.000 | 1.000 | 551 | tags=50%, list=39%, signal=81% |
| 1068 | GSE9988\_LPS\_VS\_CTRL\_TREATED\_MONOCYTE\_DN |  | 23 | 0.15 | 0.68 | 0.858 | 1.000 | 1.000 | 813 | tags=74%, list=58%, signal=172% |
| 1069 | GSE360\_CTRL\_VS\_L\_MAJOR\_MAC\_UP |  | 17 | 0.17 | 0.68 | 0.872 | 1.000 | 1.000 | 380 | tags=35%, list=27%, signal=48% |
| 1070 | GSE17580\_UNINFECTED\_VS\_S\_MANSONI\_INF\_TREG\_UP |  | 19 | 0.16 | 0.68 | 0.864 | 1.000 | 1.000 | 1081 | tags=95%, list=77%, signal=400% |
| 1071 | GSE22886\_TCELL\_VS\_BCELL\_NAIVE\_UP |  | 15 | 0.18 | 0.68 | 0.859 | 1.000 | 1.000 | 75 | tags=13%, list=5%, signal=14% |
| 1072 | GSE17721\_0.5H\_VS\_8H\_PAM3CSK4\_BMDC\_DN |  | 21 | 0.16 | 0.68 | 0.854 | 1.000 | 1.000 | 165 | tags=19%, list=12%, signal=21% |
| 1073 | GSE33162\_HDAC3\_KO\_VS\_HDAC3\_KO\_MACROPHAGE\_UP |  | 22 | 0.15 | 0.68 | 0.860 | 1.000 | 1.000 | 655 | tags=55%, list=46%, signal=100% |
| 1074 | GSE21360\_NAIVE\_VS\_QUATERNARY\_MEMORY\_CD8\_TCELL\_DN |  | 22 | 0.16 | 0.68 | 0.826 | 1.000 | 1.000 | 608 | tags=55%, list=43%, signal=94% |
| 1075 | GSE17974\_CTRL\_VS\_ACT\_IL4\_AND\_ANTI\_IL12\_24H\_CD4\_TCELL\_DN |  | 25 | 0.15 | 0.68 | 0.875 | 1.000 | 1.000 | 847 | tags=80%, list=60%, signal=197% |
| 1076 | GSE37533\_PPARG2\_FOXP3\_VS\_FOXP3\_TRANSDUCED\_CD4\_TCELL\_DN |  | 25 | 0.15 | 0.68 | 0.868 | 1.000 | 1.000 | 83 | tags=12%, list=6%, signal=13% |
| 1077 | GSE29618\_PRE\_VS\_DAY7\_POST\_LAIV\_FLU\_VACCINE\_PDC\_DN |  | 21 | 0.16 | 0.68 | 0.847 | 1.000 | 1.000 | 1101 | tags=95%, list=78%, signal=427% |
| 1078 | GSE5589\_UNSTIM\_VS\_180MIN\_LPS\_AND\_IL10\_STIM\_MACROPHAGE\_UP |  | 26 | 0.14 | 0.68 | 0.874 | 1.000 | 1.000 | 95 | tags=12%, list=7%, signal=12% |
| 1079 | GSE8835\_HEALTHY\_VS\_CLL\_CD8\_TCELL\_UP |  | 19 | 0.16 | 0.68 | 0.847 | 1.000 | 1.000 | 561 | tags=53%, list=40%, signal=86% |
| 1080 | GSE29617\_CTRL\_VS\_DAY7\_TIV\_FLU\_VACCINE\_PBMC\_2008\_DN |  | 24 | 0.15 | 0.67 | 0.877 | 1.000 | 1.000 | 1098 | tags=96%, list=78%, signal=425% |
| 1081 | GSE3920\_UNTREATED\_VS\_IFNB\_TREATED\_ENDOTHELIAL\_CELL\_DN |  | 25 | 0.15 | 0.67 | 0.866 | 1.000 | 1.000 | 755 | tags=68%, list=54%, signal=144% |
| 1082 | GSE7219\_WT\_VS\_NIK\_NFKB2\_KO\_LPS\_AND\_ANTI\_CD40\_STIM\_DC\_DN |  | 17 | 0.17 | 0.67 | 0.867 | 1.000 | 1.000 | 377 | tags=35%, list=27%, signal=48% |
| 1083 | GSE14350\_IL2RB\_KO\_VS\_WT\_TREG\_UP |  | 22 | 0.15 | 0.67 | 0.865 | 1.000 | 1.000 | 735 | tags=68%, list=52%, signal=140% |
| 1084 | GSE37532\_TREG\_VS\_TCONV\_CD4\_TCELL\_FROM\_LN\_DN |  | 27 | 0.14 | 0.67 | 0.852 | 1.000 | 1.000 | 1134 | tags=96%, list=80%, signal=481% |
| 1085 | GSE3982\_EOSINOPHIL\_VS\_MAC\_DN |  | 21 | 0.15 | 0.67 | 0.872 | 1.000 | 1.000 | 501 | tags=48%, list=36%, signal=73% |
| 1086 | GSE29618\_LAIV\_VS\_TIV\_FLU\_VACCINE\_DAY7\_PDC\_UP |  | 19 | 0.16 | 0.67 | 0.855 | 1.000 | 1.000 | 1183 | tags=100%, list=84%, signal=611% |
| 1087 | GSE1460\_NAIVE\_CD4\_TCELL\_CORD\_BLOOD\_VS\_THYMIC\_STROMAL\_CELL\_DN |  | 17 | 0.17 | 0.67 | 0.842 | 1.000 | 1.000 | 298 | tags=29%, list=21%, signal=37% |
| 1088 | GSE2770\_IL12\_AND\_TGFB\_VS\_IL4\_TREATED\_ACT\_CD4\_TCELL\_2H\_DN |  | 20 | 0.16 | 0.67 | 0.855 | 1.000 | 1.000 | 714 | tags=65%, list=51%, signal=130% |
| 1089 | GSE9509\_LPS\_VS\_LPS\_AND\_IL10\_STIM\_IL10\_KO\_MACROPHAGE\_30MIN\_UP |  | 16 | 0.17 | 0.67 | 0.839 | 1.000 | 1.000 | 65 | tags=13%, list=5%, signal=13% |
| 1090 | GSE35825\_UNTREATED\_VS\_IFNA\_STIM\_MACROPHAGE\_UP |  | 23 | 0.15 | 0.67 | 0.854 | 1.000 | 1.000 | 79 | tags=13%, list=6%, signal=14% |
| 1091 | GSE43955\_1H\_VS\_60H\_ACT\_CD4\_TCELL\_DN |  | 23 | 0.15 | 0.67 | 0.871 | 1.000 | 1.000 | 26 | tags=9%, list=2%, signal=9% |
| 1092 | GSE2405\_HEAT\_KILLED\_VS\_LIVE\_A\_PHAGOCYTOPHILUM\_STIM\_NEUTROPHIL\_9H\_UP |  | 16 | 0.17 | 0.67 | 0.878 | 1.000 | 1.000 | 529 | tags=50%, list=37%, signal=79% |
| 1093 | GSE25677\_R848\_VS\_MPL\_AND\_R848\_STIM\_BCELL\_DN |  | 21 | 0.16 | 0.67 | 0.863 | 1.000 | 1.000 | 557 | tags=48%, list=39%, signal=78% |
| 1094 | GSE7218\_IGM\_VS\_IGG\_SIGNAL\_THGOUGH\_ANTIGEN\_BCELL\_UP |  | 18 | 0.16 | 0.66 | 0.863 | 1.000 | 1.000 | 797 | tags=78%, list=56%, signal=176% |
| 1095 | GSE27786\_CD8\_TCELL\_VS\_NKTCELL\_UP |  | 27 | 0.14 | 0.66 | 0.870 | 1.000 | 1.000 | 593 | tags=48%, list=42%, signal=81% |
| 1096 | GSE1432\_1H\_VS\_6H\_IFNG\_MICROGLIA\_UP |  | 17 | 0.17 | 0.66 | 0.845 | 1.000 | 1.000 | 202 | tags=24%, list=14%, signal=27% |
| 1097 | GSE40273\_XBP1\_KO\_VS\_WT\_TREG\_DN |  | 22 | 0.15 | 0.66 | 0.885 | 1.000 | 1.000 | 51 | tags=9%, list=4%, signal=9% |
| 1098 | GSE17580\_UNINFECTED\_VS\_S\_MANSONI\_INF\_TEFF\_DN |  | 19 | 0.16 | 0.66 | 0.873 | 1.000 | 1.000 | 412 | tags=37%, list=29%, signal=51% |
| 1099 | GSE24574\_BCL6\_HIGH\_TFH\_VS\_TCONV\_CD4\_TCELL\_UP |  | 18 | 0.16 | 0.66 | 0.879 | 1.000 | 1.000 | 454 | tags=44%, list=32%, signal=65% |
| 1100 | GSE5960\_TH1\_VS\_ANERGIC\_TH1\_UP |  | 15 | 0.17 | 0.66 | 0.886 | 1.000 | 1.000 | 1169 | tags=100%, list=83%, signal=577% |
| 1101 | GSE12003\_4D\_VS\_8D\_CULTURE\_MIR223\_KO\_BM\_PROGENITOR\_DN |  | 17 | 0.17 | 0.66 | 0.878 | 1.000 | 1.000 | 63 | tags=12%, list=4%, signal=12% |
| 1102 | GSE40666\_UNTREATED\_VS\_IFNA\_STIM\_CD8\_TCELL\_90MIN\_DN |  | 23 | 0.15 | 0.66 | 0.841 | 1.000 | 1.000 | 818 | tags=78%, list=58%, signal=183% |
| 1103 | GSE13484\_3H\_UNSTIM\_VS\_YF17D\_VACCINE\_STIM\_PBMC\_UP |  | 23 | 0.15 | 0.66 | 0.880 | 1.000 | 1.000 | 636 | tags=57%, list=45%, signal=101% |
| 1104 | GSE3920\_IFNB\_VS\_IFNG\_TREATED\_ENDOTHELIAL\_CELL\_DN |  | 22 | 0.15 | 0.66 | 0.862 | 1.000 | 1.000 | 646 | tags=59%, list=46%, signal=107% |
| 1105 | GSE19888\_CTRL\_VS\_A3R\_INHIBITOR\_TREATED\_MAST\_CELL\_UP |  | 20 | 0.15 | 0.66 | 0.887 | 1.000 | 1.000 | 55 | tags=10%, list=4%, signal=10% |
| 1106 | GSE35685\_CD34POS\_CD10NEG\_CD62LPOS\_VS\_CD34POS\_CD10POS\_BONE\_MARROW\_DN |  | 24 | 0.15 | 0.65 | 0.891 | 1.000 | 1.000 | 88 | tags=13%, list=6%, signal=13% |
| 1107 | GSE41867\_NAIVE\_VS\_EFFECTOR\_CD8\_TCELL\_UP |  | 27 | 0.14 | 0.65 | 0.887 | 1.000 | 1.000 | 834 | tags=74%, list=59%, signal=178% |
| 1108 | GSE41978\_ID2\_KO\_VS\_ID2\_KO\_AND\_BIM\_KO\_KLRG1\_LOW\_EFFECTOR\_CD8\_TCELL\_DN |  | 17 | 0.16 | 0.65 | 0.858 | 1.000 | 1.000 | 885 | tags=82%, list=63%, signal=218% |
| 1109 | GSE17721\_PAM3CSK4\_VS\_CPG\_24H\_BMDC\_DN |  | 18 | 0.16 | 0.65 | 0.884 | 1.000 | 1.000 | 208 | tags=22%, list=15%, signal=26% |
| 1110 | GSE21927\_SPLEEN\_C57BL6\_VS\_EL4\_TUMOR\_BALBC\_MONOCYTES\_DN |  | 19 | 0.16 | 0.65 | 0.873 | 1.000 | 1.000 | 75 | tags=11%, list=5%, signal=11% |
| 1111 | GSE43260\_BTLA\_POS\_VS\_NEG\_INTRATUMORAL\_CD8\_TCELL\_UP |  | 22 | 0.15 | 0.65 | 0.873 | 1.000 | 1.000 | 193 | tags=18%, list=14%, signal=21% |
| 1112 | GSE42021\_CD24HI\_VS\_CD24LOW\_TREG\_THYMUS\_DN |  | 29 | 0.13 | 0.65 | 0.890 | 1.000 | 1.000 | 71 | tags=10%, list=5%, signal=11% |
| 1113 | GSE19401\_UNSTIM\_VS\_RETINOIC\_ACID\_AND\_PAM2CSK4\_STIM\_FOLLICULAR\_DC\_UP |  | 15 | 0.17 | 0.65 | 0.900 | 1.000 | 1.000 | 206 | tags=20%, list=15%, signal=23% |
| 1114 | GSE16451\_IMMATURE\_VS\_MATURE\_NEURON\_CELL\_LINE\_WEST\_EQUINE\_ENC\_VIRUS\_DN |  | 35 | 0.13 | 0.65 | 0.892 | 1.000 | 1.000 | 786 | tags=69%, list=56%, signal=151% |
| 1115 | GSE36888\_UNTREATED\_VS\_IL2\_TREATED\_STAT5\_AB\_KNOCKIN\_TCELL\_17H\_DN |  | 17 | 0.16 | 0.65 | 0.878 | 1.000 | 1.000 | 550 | tags=47%, list=39%, signal=76% |
| 1116 | GSE18791\_CTRL\_VS\_NEWCASTLE\_VIRUS\_DC\_4H\_DN |  | 27 | 0.14 | 0.65 | 0.887 | 1.000 | 1.000 | 377 | tags=30%, list=27%, signal=40% |
| 1117 | GSE43955\_1H\_VS\_20H\_ACT\_CD4\_TCELL\_UP |  | 25 | 0.14 | 0.65 | 0.910 | 1.000 | 1.000 | 372 | tags=32%, list=26%, signal=43% |
| 1118 | GSE21546\_UNSTIM\_VS\_ANTI\_CD3\_STIM\_ELK1\_KO\_DP\_THYMOCYTES\_UP |  | 18 | 0.16 | 0.64 | 0.909 | 1.000 | 1.000 | 263 | tags=28%, list=19%, signal=34% |
| 1119 | GSE3982\_EOSINOPHIL\_VS\_NEUTROPHIL\_UP |  | 24 | 0.14 | 0.64 | 0.906 | 1.000 | 1.000 | 28 | tags=8%, list=2%, signal=8% |
| 1120 | GSE34006\_WT\_VS\_A2AR\_KO\_TREG\_UP |  | 17 | 0.16 | 0.64 | 0.892 | 1.000 | 1.000 | 172 | tags=18%, list=12%, signal=20% |
| 1121 | GSE2770\_IL12\_AND\_TGFB\_VS\_IL4\_TREATED\_ACT\_CD4\_TCELL\_6H\_UP |  | 15 | 0.17 | 0.64 | 0.872 | 1.000 | 1.000 | 96 | tags=13%, list=7%, signal=14% |
| 1122 | GSE36392\_TYPE\_2\_MYELOID\_VS\_EOSINOPHIL\_IL25\_TREATED\_LUNG\_UP |  | 18 | 0.16 | 0.64 | 0.879 | 1.000 | 1.000 | 229 | tags=22%, list=16%, signal=26% |
| 1123 | GSE13485\_CTRL\_VS\_DAY21\_YF17D\_VACCINE\_PBMC\_UP |  | 18 | 0.16 | 0.64 | 0.886 | 1.000 | 1.000 | 130 | tags=17%, list=9%, signal=18% |
| 1124 | GSE17974\_CTRL\_VS\_ACT\_IL4\_AND\_ANTI\_IL12\_48H\_CD4\_TCELL\_DN |  | 25 | 0.14 | 0.64 | 0.896 | 1.000 | 1.000 | 529 | tags=44%, list=37%, signal=69% |
| 1125 | GSE1925\_CTRL\_VS\_IFNG\_PRIMED\_MACROPHAGE\_3H\_IFNG\_STIM\_UP |  | 21 | 0.15 | 0.64 | 0.895 | 1.000 | 1.000 | 826 | tags=76%, list=59%, signal=181% |
| 1126 | GSE360\_CTRL\_VS\_T\_GONDII\_DC\_UP |  | 26 | 0.14 | 0.64 | 0.889 | 1.000 | 1.000 | 257 | tags=23%, list=18%, signal=28% |
| 1127 | GSE6674\_UNSTIM\_VS\_PL2\_3\_STIM\_BCELL\_DN |  | 18 | 0.16 | 0.64 | 0.906 | 1.000 | 1.000 | 139 | tags=17%, list=10%, signal=18% |
| 1128 | KAECH\_DAY15\_EFF\_VS\_MEMORY\_CD8\_TCELL\_DN |  | 16 | 0.16 | 0.64 | 0.896 | 1.000 | 1.000 | 280 | tags=25%, list=20%, signal=31% |
| 1129 | GSE16385\_MONOCYTE\_VS\_12H\_ROSIGLITAZONE\_IL4\_TREATED\_MACROPHAGE\_UP |  | 18 | 0.16 | 0.63 | 0.887 | 1.000 | 1.000 | 1003 | tags=89%, list=71%, signal=303% |
| 1130 | GSE43863\_NAIVE\_VS\_MEMORY\_TFH\_CD4\_TCELL\_D150\_LCMV\_DN |  | 28 | 0.13 | 0.63 | 0.918 | 1.000 | 1.000 | 19 | tags=7%, list=1%, signal=7% |
| 1131 | GSE21063\_WT\_VS\_NFATC1\_KO\_8H\_ANTI\_IGM\_STIM\_BCELL\_UP |  | 20 | 0.15 | 0.63 | 0.917 | 1.000 | 1.000 | 98 | tags=10%, list=7%, signal=11% |
| 1132 | GSE3203\_HEALTHY\_VS\_INFLUENZA\_INFECTED\_LN\_BCELL\_UP |  | 21 | 0.15 | 0.63 | 0.899 | 1.000 | 1.000 | 47 | tags=10%, list=3%, signal=10% |
| 1133 | GSE9988\_LOW\_LPS\_VS\_CTRL\_TREATED\_MONOCYTE\_DN |  | 19 | 0.15 | 0.63 | 0.907 | 1.000 | 1.000 | 611 | tags=53%, list=43%, signal=92% |
| 1134 | GSE22443\_IL2\_VS\_IL12\_TREATED\_ACT\_CD8\_TCELL\_DN |  | 16 | 0.16 | 0.63 | 0.880 | 1.000 | 1.000 | 1027 | tags=94%, list=73%, signal=341% |
| 1135 | GSE17721\_POLYIC\_VS\_PAM3CSK4\_16H\_BMDC\_UP |  | 20 | 0.15 | 0.63 | 0.906 | 1.000 | 1.000 | 63 | tags=10%, list=4%, signal=10% |
| 1136 | GSE27859\_DC\_VS\_CD11C\_INT\_F480\_INT\_DC\_DN |  | 18 | 0.16 | 0.63 | 0.875 | 1.000 | 1.000 | 70 | tags=11%, list=5%, signal=12% |
| 1137 | GSE22033\_UNTREATED\_VS\_ROSIGLITAZONE\_TREATED\_MEF\_UP |  | 18 | 0.15 | 0.63 | 0.902 | 1.000 | 1.000 | 997 | tags=89%, list=71%, signal=299% |
| 1138 | GSE22935\_24H\_VS\_48H\_MBOVIS\_BCG\_STIM\_MACROPHAGE\_UP |  | 16 | 0.17 | 0.62 | 0.914 | 1.000 | 1.000 | 233 | tags=25%, list=17%, signal=30% |
| 1139 | GSE27786\_LSK\_VS\_CD8\_TCELL\_UP |  | 22 | 0.14 | 0.62 | 0.917 | 1.000 | 1.000 | 578 | tags=50%, list=41%, signal=83% |
| 1140 | GSE45837\_WT\_VS\_GFI1\_KO\_PDC\_DN |  | 19 | 0.15 | 0.62 | 0.914 | 1.000 | 1.000 | 1097 | tags=95%, list=78%, signal=420% |
| 1141 | GSE3982\_EFF\_MEMORY\_CD4\_TCELL\_VS\_TH2\_DN |  | 24 | 0.14 | 0.62 | 0.919 | 1.000 | 1.000 | 890 | tags=83%, list=63%, signal=222% |
| 1142 | GSE6269\_HEALTHY\_VS\_STAPH\_AUREUS\_INF\_PBMC\_UP |  | 22 | 0.14 | 0.62 | 0.915 | 1.000 | 1.000 | 635 | tags=55%, list=45%, signal=98% |
| 1143 | GSE37533\_UNTREATED\_VS\_PIOGLIZATONE\_TREATED\_CD4\_TCELL\_FOXP3\_TRASDUCED\_CD4\_TCELL\_UP |  | 18 | 0.15 | 0.62 | 0.913 | 1.000 | 1.000 | 1195 | tags=100%, list=85%, signal=645% |
| 1144 | GSE46242\_CTRL\_VS\_EGR2\_DELETED\_TH1\_CD4\_TCELL\_UP |  | 17 | 0.16 | 0.62 | 0.918 | 1.000 | 1.000 | 386 | tags=35%, list=27%, signal=48% |
| 1145 | GSE40666\_UNTREATED\_VS\_IFNA\_STIM\_STAT4\_KO\_EFFECTOR\_CD8\_TCELL\_90MIN\_DN |  | 24 | 0.14 | 0.62 | 0.899 | 1.000 | 1.000 | 566 | tags=50%, list=40%, signal=82% |
| 1146 | GSE3982\_EOSINOPHIL\_VS\_EFF\_MEMORY\_CD4\_TCELL\_DN |  | 18 | 0.15 | 0.62 | 0.914 | 1.000 | 1.000 | 717 | tags=67%, list=51%, signal=134% |
| 1147 | GSE3982\_DC\_VS\_TH1\_DN |  | 16 | 0.16 | 0.62 | 0.883 | 1.000 | 1.000 | 84 | tags=13%, list=6%, signal=13% |
| 1148 | GSE28130\_ACTIVATED\_VS\_INDUCEED\_TREG\_DN |  | 19 | 0.15 | 0.62 | 0.921 | 1.000 | 1.000 | 57 | tags=11%, list=4%, signal=11% |
| 1149 | GSE12845\_PRE\_GC\_VS\_DARKZONE\_GC\_TONSIL\_BCELL\_UP |  | 15 | 0.16 | 0.62 | 0.923 | 1.000 | 1.000 | 2 | tags=7%, list=0%, signal=7% |
| 1150 | GSE11057\_PBMC\_VS\_MEM\_CD4\_TCELL\_DN |  | 22 | 0.14 | 0.62 | 0.906 | 1.000 | 1.000 | 126 | tags=14%, list=9%, signal=15% |
| 1151 | GSE35685\_CD34POS\_CD38NEG\_VS\_CD34POS\_CD10NEG\_CD62LPOS\_BONE\_MARROW\_UP |  | 30 | 0.13 | 0.62 | 0.905 | 1.000 | 1.000 | 93 | tags=10%, list=7%, signal=10% |
| 1152 | GSE1740\_MCSF\_VS\_MCSF\_AND\_IFNG\_DAY2\_DERIVED\_MACROPHAGE\_DN |  | 25 | 0.13 | 0.62 | 0.899 | 1.000 | 1.000 | 308 | tags=28%, list=22%, signal=35% |
| 1153 | GSE7831\_UNSTIM\_VS\_INFLUENZA\_STIM\_PDC\_1H\_DN |  | 19 | 0.15 | 0.62 | 0.915 | 1.000 | 1.000 | 1071 | tags=95%, list=76%, signal=388% |
| 1154 | GSE9239\_CTRL\_VS\_TNF\_INHIBITOR\_TREATED\_DC\_DN |  | 22 | 0.14 | 0.62 | 0.895 | 1.000 | 1.000 | 230 | tags=23%, list=16%, signal=27% |
| 1155 | GSE29949\_MICROGLIA\_BRAIN\_VS\_CD8\_POS\_DC\_SPLEEN\_DN |  | 20 | 0.14 | 0.61 | 0.925 | 1.000 | 1.000 | 355 | tags=30%, list=25%, signal=40% |
| 1156 | GSE13493\_CD4INTCD8POS\_VS\_CD8POS\_THYMOCYTE\_UP |  | 16 | 0.16 | 0.61 | 0.904 | 1.000 | 1.000 | 377 | tags=31%, list=27%, signal=42% |
| 1157 | GSE25088\_WT\_VS\_STAT6\_KO\_MACROPHAGE\_DN |  | 16 | 0.16 | 0.61 | 0.925 | 1.000 | 1.000 | 1 | tags=6%, list=0%, signal=6% |
| 1158 | GSE4748\_CTRL\_VS\_CYANOBACTERIUM\_LPSLIKE\_STIM\_DC\_3H\_DN |  | 21 | 0.14 | 0.61 | 0.907 | 1.000 | 1.000 | 1215 | tags=100%, list=86%, signal=709% |
| 1159 | GSE44732\_UNSTIM\_VS\_IL27\_STIM\_IMATURE\_DC\_UP |  | 22 | 0.14 | 0.61 | 0.905 | 1.000 | 1.000 | 528 | tags=45%, list=37%, signal=72% |
| 1160 | GSE11961\_PLASMA\_CELL\_DAY7\_VS\_GERMINAL\_CENTER\_BCELL\_DAY40\_DN |  | 15 | 0.16 | 0.61 | 0.936 | 1.000 | 1.000 | 203 | tags=20%, list=14%, signal=23% |
| 1161 | GSE37534\_UNTREATED\_VS\_GW1929\_TREATED\_CD4\_TCELL\_PPARG1\_AND\_FOXP3\_TRASDUCED\_DN |  | 27 | 0.13 | 0.61 | 0.909 | 1.000 | 1.000 | 658 | tags=56%, list=47%, signal=102% |
| 1162 | GSE41867\_NAIVE\_VS\_DAY15\_LCMV\_EFFECTOR\_CD8\_TCELL\_DN |  | 21 | 0.14 | 0.61 | 0.946 | 1.000 | 1.000 | 1104 | tags=95%, list=78%, signal=431% |
| 1163 | GSE24634\_IL4\_VS\_CTRL\_TREATED\_NAIVE\_CD4\_TCELL\_DAY7\_UP |  | 19 | 0.15 | 0.61 | 0.913 | 1.000 | 1.000 | 794 | tags=74%, list=56%, signal=166% |
| 1164 | GSE46606\_DAY1\_VS\_DAY3\_CD40L\_IL2\_IL5\_STIMULATED\_BCELL\_DN |  | 28 | 0.13 | 0.61 | 0.906 | 1.000 | 1.000 | 28 | tags=7%, list=2%, signal=7% |
| 1165 | GSE7509\_DC\_VS\_MONOCYTE\_WITH\_FCGRIIB\_STIM\_DN |  | 31 | 0.12 | 0.61 | 0.931 | 1.000 | 1.000 | 63 | tags=10%, list=4%, signal=10% |
| 1166 | GSE28237\_FOLLICULAR\_VS\_EARLY\_GC\_BCELL\_DN |  | 30 | 0.13 | 0.61 | 0.915 | 1.000 | 1.000 | 1068 | tags=93%, list=76%, signal=376% |
| 1167 | GSE23321\_CD8\_STEM\_CELL\_MEMORY\_VS\_NAIVE\_CD8\_TCELL\_UP |  | 18 | 0.15 | 0.61 | 0.916 | 1.000 | 1.000 | 1198 | tags=100%, list=85%, signal=654% |
| 1168 | GSE41176\_UNSTIM\_VS\_ANTI\_IGM\_STIM\_BCELL\_6H\_DN |  | 19 | 0.14 | 0.60 | 0.912 | 1.000 | 1.000 | 452 | tags=37%, list=32%, signal=53% |
| 1169 | GSE27786\_CD4\_TCELL\_VS\_ERYTHTROBLAST\_UP |  | 25 | 0.13 | 0.60 | 0.924 | 1.000 | 1.000 | 427 | tags=36%, list=30%, signal=51% |
| 1170 | GSE15750\_DAY6\_VS\_DAY10\_TRAF6KO\_EFF\_CD8\_TCELL\_UP |  | 17 | 0.15 | 0.60 | 0.907 | 1.000 | 1.000 | 1 | tags=6%, list=0%, signal=6% |
| 1171 | GSE2770\_TGFB\_AND\_IL4\_VS\_TGFB\_AND\_IL12\_TREATED\_ACT\_CD4\_TCELL\_6H\_DN |  | 23 | 0.14 | 0.60 | 0.929 | 1.000 | 1.000 | 379 | tags=30%, list=27%, signal=41% |
| 1172 | GSE360\_CTRL\_VS\_M\_TUBERCULOSIS\_MAC\_UP |  | 22 | 0.14 | 0.60 | 0.908 | 1.000 | 1.000 | 919 | tags=82%, list=65%, signal=231% |
| 1173 | GSE17721\_CTRL\_VS\_POLYIC\_2H\_BMDC\_DN |  | 20 | 0.14 | 0.60 | 0.929 | 1.000 | 1.000 | 264 | tags=25%, list=19%, signal=30% |
| 1174 | GSE12001\_MIR223\_KO\_VS\_WT\_NEUTROPHIL\_DN |  | 18 | 0.15 | 0.60 | 0.926 | 1.000 | 1.000 | 70 | tags=11%, list=5%, signal=12% |
| 1175 | GSE22919\_RESTING\_VS\_IL2\_IL12\_IL15\_STIM\_NK\_CELL\_UP |  | 26 | 0.13 | 0.60 | 0.921 | 1.000 | 1.000 | 563 | tags=46%, list=40%, signal=75% |
| 1176 | GSE32986\_UNSTIM\_VS\_GMCSF\_STIM\_DC\_DN |  | 33 | 0.12 | 0.60 | 0.919 | 1.000 | 1.000 | 258 | tags=21%, list=18%, signal=25% |
| 1177 | GSE12845\_IGD\_NEG\_BLOOD\_VS\_NAIVE\_TONSIL\_BCELL\_DN |  | 23 | 0.14 | 0.60 | 0.924 | 1.000 | 1.000 | 723 | tags=65%, list=51%, signal=132% |
| 1178 | GSE25123\_CTRL\_VS\_IL4\_AND\_ROSIGLITAZONE\_STIM\_MACROPHAGE\_UP |  | 19 | 0.14 | 0.60 | 0.928 | 1.000 | 1.000 | 330 | tags=26%, list=23%, signal=34% |
| 1179 | GSE13485\_DAY7\_VS\_DAY21\_YF17D\_VACCINE\_PBMC\_UP |  | 26 | 0.13 | 0.60 | 0.931 | 1.000 | 1.000 | 263 | tags=23%, list=19%, signal=28% |
| 1180 | GSE32423\_MEMORY\_VS\_NAIVE\_CD8\_TCELL\_DN |  | 18 | 0.14 | 0.60 | 0.937 | 1.000 | 1.000 | 295 | tags=28%, list=21%, signal=35% |
| 1181 | GSE15930\_NAIVE\_VS\_24H\_IN\_VITRO\_STIM\_IL12\_CD8\_TCELL\_DN |  | 21 | 0.14 | 0.60 | 0.934 | 1.000 | 1.000 | 1022 | tags=90%, list=72%, signal=323% |
| 1182 | GSE39382\_IL3\_VS\_IL3\_IL33\_TREATED\_MAST\_CELL\_UP |  | 21 | 0.14 | 0.60 | 0.916 | 1.000 | 1.000 | 256 | tags=24%, list=18%, signal=29% |
| 1183 | GSE16385\_ROSIGLITAZONE\_IL4\_VS\_IL4\_ALONE\_STIM\_MACROPHAGE\_12H\_UP |  | 22 | 0.14 | 0.60 | 0.960 | 1.000 | 1.000 | 175 | tags=18%, list=12%, signal=20% |
| 1184 | GSE32423\_IL7\_VS\_IL7\_IL4\_MEMORY\_CD8\_TCELL\_UP |  | 23 | 0.13 | 0.60 | 0.936 | 1.000 | 1.000 | 199 | tags=17%, list=14%, signal=20% |
| 1185 | GSE411\_UNSTIM\_VS\_100MIN\_IL6\_STIM\_SOCS3\_KO\_MACROPHAGE\_UP |  | 23 | 0.13 | 0.60 | 0.912 | 1.000 | 1.000 | 1145 | tags=96%, list=81%, signal=499% |
| 1186 | GSE7764\_NKCELL\_VS\_SPLENOCYTE\_DN |  | 18 | 0.15 | 0.60 | 0.926 | 1.000 | 1.000 | 1 | tags=6%, list=0%, signal=5% |
| 1187 | GSE26669\_CD4\_VS\_CD8\_TCELL\_IN\_MLR\_DN |  | 20 | 0.14 | 0.60 | 0.933 | 1.000 | 1.000 | 344 | tags=30%, list=24%, signal=39% |
| 1188 | GSE17721\_0.5H\_VS\_8H\_GARDIQUIMOD\_BMDC\_UP |  | 21 | 0.14 | 0.59 | 0.934 | 1.000 | 1.000 | 1036 | tags=90%, list=73%, signal=335% |
| 1189 | GSE22935\_WT\_VS\_MYD88\_KO\_MACROPHAGE\_12H\_MBOVIS\_BCG\_STIM\_UP |  | 30 | 0.12 | 0.59 | 0.933 | 1.000 | 1.000 | 74 | tags=10%, list=5%, signal=10% |
| 1190 | GSE46606\_IRF4\_KO\_VS\_WT\_UNSTIM\_BCELL\_UP |  | 19 | 0.14 | 0.59 | 0.928 | 1.000 | 1.000 | 502 | tags=42%, list=36%, signal=64% |
| 1191 | GSE360\_CTRL\_VS\_B\_MALAYI\_HIGH\_DOSE\_MAC\_DN |  | 23 | 0.13 | 0.59 | 0.929 | 1.000 | 1.000 | 511 | tags=43%, list=36%, signal=67% |
| 1192 | GSE3920\_UNTREATED\_VS\_IFNA\_TREATED\_ENDOTHELIAL\_CELL\_UP |  | 17 | 0.15 | 0.59 | 0.926 | 1.000 | 1.000 | 382 | tags=35%, list=27%, signal=48% |
| 1193 | GSE30083\_SP1\_VS\_SP3\_THYMOCYTE\_UP |  | 17 | 0.15 | 0.59 | 0.925 | 1.000 | 1.000 | 165 | tags=18%, list=12%, signal=20% |
| 1194 | GSE16266\_LPS\_VS\_HEATSHOCK\_AND\_LPS\_STIM\_MEF\_UP |  | 17 | 0.15 | 0.59 | 0.931 | 1.000 | 1.000 | 1 | tags=6%, list=0%, signal=6% |
| 1195 | GSE36888\_UNTREATED\_VS\_IL2\_TREATED\_STAT5\_AB\_KNOCKIN\_TCELL\_2H\_DN |  | 18 | 0.15 | 0.59 | 0.927 | 1.000 | 1.000 | 66 | tags=11%, list=5%, signal=12% |
| 1196 | GSE32423\_MEMORY\_VS\_NAIVE\_CD8\_TCELL\_IL7\_IL4\_UP |  | 15 | 0.15 | 0.59 | 0.934 | 1.000 | 1.000 | 1194 | tags=100%, list=85%, signal=643% |
| 1197 | GSE19888\_ADENOSINE\_A3R\_INH\_VS\_ACT\_WITH\_INHIBITOR\_PRETREATMENT\_IN\_MAST\_CELL\_UP |  | 16 | 0.15 | 0.59 | 0.919 | 1.000 | 1.000 | 258 | tags=25%, list=18%, signal=30% |
| 1198 | GSE30971\_CTRL\_VS\_LPS\_STIM\_MACROPHAGE\_WBP7\_HET\_4H\_UP |  | 23 | 0.13 | 0.59 | 0.942 | 1.000 | 1.000 | 612 | tags=52%, list=43%, signal=91% |
| 1199 | GSE15215\_CD2\_POS\_VS\_NEG\_PDC\_DN |  | 26 | 0.13 | 0.59 | 0.942 | 1.000 | 1.000 | 500 | tags=42%, list=35%, signal=64% |
| 1200 | GSE14000\_UNSTIM\_VS\_4H\_LPS\_DC\_DN |  | 26 | 0.13 | 0.59 | 0.941 | 1.000 | 1.000 | 315 | tags=27%, list=22%, signal=34% |
| 1201 | GSE360\_L\_MAJOR\_VS\_B\_MALAYI\_LOW\_DOSE\_MAC\_UP |  | 17 | 0.15 | 0.59 | 0.934 | 1.000 | 1.000 | 366 | tags=29%, list=26%, signal=39% |
| 1202 | GSE36891\_POLYIC\_TLR3\_VS\_PAM\_TLR2\_STIM\_PERITONEAL\_MACROPHAGE\_DN |  | 20 | 0.14 | 0.59 | 0.942 | 1.000 | 1.000 | 658 | tags=60%, list=47%, signal=111% |
| 1203 | GSE43955\_TH0\_VS\_TGFB\_IL6\_TH17\_ACT\_CD4\_TCELL\_20H\_UP |  | 21 | 0.13 | 0.58 | 0.949 | 1.000 | 1.000 | 131 | tags=14%, list=9%, signal=16% |
| 1204 | GSE7568\_IL4\_TGFB\_DEXAMETHASONE\_VS\_IL4\_TGFB\_TREATED\_MACROPHAGE\_DN |  | 18 | 0.14 | 0.58 | 0.933 | 1.000 | 1.000 | 184 | tags=17%, list=13%, signal=19% |
| 1205 | GSE16522\_MEMORY\_VS\_NAIVE\_ANTI\_CD3CD28\_STIM\_CD8\_TCELL\_UP |  | 20 | 0.14 | 0.58 | 0.930 | 1.000 | 1.000 | 300 | tags=25%, list=21%, signal=31% |
| 1206 | GSE26343\_WT\_VS\_NFAT5\_KO\_MACROPHAGE\_UP |  | 19 | 0.14 | 0.58 | 0.935 | 1.000 | 1.000 | 729 | tags=68%, list=52%, signal=140% |
| 1207 | GSE29618\_PRE\_VS\_DAY7\_FLU\_VACCINE\_BCELL\_DN |  | 17 | 0.14 | 0.58 | 0.946 | 1.000 | 1.000 | 1 | tags=6%, list=0%, signal=6% |
| 1208 | GSE40666\_NAIVE\_VS\_EFFECTOR\_CD8\_TCELL\_WITH\_IFNA\_STIM\_90MIN\_UP |  | 18 | 0.14 | 0.58 | 0.937 | 1.000 | 1.000 | 1213 | tags=100%, list=86%, signal=704% |
| 1209 | GSE22886\_NAIVE\_CD4\_TCELL\_VS\_12H\_ACT\_TH2\_DN |  | 20 | 0.13 | 0.58 | 0.926 | 1.000 | 1.000 | 871 | tags=80%, list=62%, signal=206% |
| 1210 | GSE2826\_WT\_VS\_BTK\_KO\_BCELL\_UP |  | 20 | 0.13 | 0.58 | 0.957 | 1.000 | 1.000 | 292 | tags=25%, list=21%, signal=31% |
| 1211 | GSE12845\_IGD\_NEG\_BLOOD\_VS\_DARKZONE\_GC\_TONSIL\_BCELL\_DN |  | 20 | 0.14 | 0.57 | 0.960 | 1.000 | 1.000 | 159 | tags=15%, list=11%, signal=17% |
| 1212 | GSE22601\_DOUBLE\_NEGATIVE\_VS\_IMMATURE\_CD4\_SP\_THYMOCYTE\_UP |  | 21 | 0.13 | 0.57 | 0.944 | 1.000 | 1.000 | 53 | tags=10%, list=4%, signal=10% |
| 1213 | GSE10325\_MYELOID\_VS\_LUPUS\_MYELOID\_DN |  | 27 | 0.12 | 0.57 | 0.945 | 1.000 | 1.000 | 1240 | tags=100%, list=88%, signal=809% |
| 1214 | GSE17974\_CTRL\_VS\_ACT\_IL4\_AND\_ANTI\_IL12\_0.5H\_CD4\_TCELL\_DN |  | 22 | 0.13 | 0.57 | 0.960 | 1.000 | 1.000 | 575 | tags=45%, list=41%, signal=76% |
| 1215 | GSE29618\_PRE\_VS\_DAY7\_POST\_LAIV\_FLU\_VACCINE\_BCELL\_UP |  | 18 | 0.14 | 0.57 | 0.946 | 1.000 | 1.000 | 160 | tags=17%, list=11%, signal=19% |
| 1216 | GSE22443\_NAIVE\_VS\_ACT\_AND\_IL12\_TREATED\_CD8\_TCELL\_DN |  | 21 | 0.14 | 0.57 | 0.945 | 1.000 | 1.000 | 416 | tags=33%, list=29%, signal=47% |
| 1217 | GSE32034\_LY6C\_HIGH\_VS\_LOW\_MONOCYTE\_DN |  | 21 | 0.13 | 0.57 | 0.951 | 1.000 | 1.000 | 456 | tags=38%, list=32%, signal=55% |
| 1218 | GSE1791\_CTRL\_VS\_NEUROMEDINU\_IN\_T\_CELL\_LINE\_3H\_DN |  | 16 | 0.14 | 0.57 | 0.942 | 1.000 | 1.000 | 419 | tags=38%, list=30%, signal=53% |
| 1219 | GSE36476\_CTRL\_VS\_TSST\_ACT\_16H\_MEMORY\_CD4\_TCELL\_OLD\_DN |  | 30 | 0.11 | 0.57 | 0.946 | 1.000 | 1.000 | 512 | tags=40%, list=36%, signal=61% |
| 1220 | GSE9037\_CTRL\_VS\_LPS\_1H\_STIM\_BMDM\_UP |  | 23 | 0.13 | 0.57 | 0.963 | 1.000 | 1.000 | 482 | tags=39%, list=34%, signal=58% |
| 1221 | GSE21927\_UNTREATED\_VS\_GMCSF\_IL6\_TREATED\_BONE\_MARROW\_UP |  | 18 | 0.14 | 0.57 | 0.938 | 1.000 | 1.000 | 80 | tags=11%, list=6%, signal=12% |
| 1222 | GSE3982\_EOSINOPHIL\_VS\_DC\_DN |  | 26 | 0.12 | 0.57 | 0.947 | 1.000 | 1.000 | 512 | tags=42%, list=36%, signal=65% |
| 1223 | GSE8685\_IL2\_STARVED\_VS\_IL2\_ACT\_IL2\_STARVED\_CD4\_TCELL\_DN |  | 20 | 0.13 | 0.57 | 0.951 | 1.000 | 1.000 | 791 | tags=75%, list=56%, signal=168% |
| 1224 | GSE17721\_CTRL\_VS\_POLYIC\_0.5H\_BMDC\_DN |  | 17 | 0.14 | 0.56 | 0.948 | 1.000 | 1.000 | 465 | tags=41%, list=33%, signal=61% |
| 1225 | GSE17186\_BLOOD\_VS\_CORD\_BLOOD\_NAIVE\_BCELL\_DN |  | 24 | 0.13 | 0.56 | 0.954 | 1.000 | 1.000 | 772 | tags=67%, list=55%, signal=145% |
| 1226 | GSE15930\_NAIVE\_VS\_24H\_IN\_VITRO\_STIM\_CD8\_TCELL\_DN |  | 22 | 0.13 | 0.56 | 0.960 | 1.000 | 1.000 | 1 | tags=5%, list=0%, signal=4% |
| 1227 | GSE15930\_NAIVE\_VS\_72H\_IN\_VITRO\_STIM\_CD8\_TCELL\_DN |  | 21 | 0.13 | 0.56 | 0.966 | 1.000 | 1.000 | 1 | tags=5%, list=0%, signal=5% |
| 1228 | GSE13762\_CTRL\_VS\_125\_VITAMIND\_DAY12\_DC\_UP |  | 16 | 0.14 | 0.55 | 0.954 | 1.000 | 1.000 | 226 | tags=19%, list=16%, signal=22% |
| 1229 | GSE17186\_NAIVE\_VS\_CD21HIGH\_TRANSITIONAL\_BCELL\_CORD\_BLOOD\_UP |  | 19 | 0.13 | 0.55 | 0.955 | 1.000 | 1.000 | 572 | tags=47%, list=41%, signal=79% |
| 1230 | GSE2770\_TGFB\_AND\_IL4\_VS\_IL4\_TREATED\_ACT\_CD4\_TCELL\_2H\_UP |  | 16 | 0.14 | 0.55 | 0.939 | 1.000 | 1.000 | 595 | tags=56%, list=42%, signal=96% |
| 1231 | GSE22886\_NAIVE\_CD4\_TCELL\_VS\_MEMORY\_TCELL\_DN |  | 25 | 0.12 | 0.55 | 0.958 | 1.000 | 1.000 | 1247 | tags=100%, list=88%, signal=845% |
| 1232 | GSE13547\_WT\_VS\_ZFX\_KO\_BCELL\_DN |  | 21 | 0.13 | 0.55 | 0.959 | 1.000 | 1.000 | 1 | tags=5%, list=0%, signal=5% |
| 1233 | GSE25147\_UNSTIM\_VS\_HELIOBACTER\_PYLORI\_LPS\_STIM\_MKN45\_CELL\_UP |  | 16 | 0.14 | 0.55 | 0.959 | 1.000 | 1.000 | 722 | tags=63%, list=51%, signal=127% |
| 1234 | GSE6259\_BCELL\_VS\_CD4\_TCELL\_DN |  | 29 | 0.11 | 0.55 | 0.967 | 1.000 | 1.000 | 220 | tags=17%, list=16%, signal=20% |
| 1235 | GSE37532\_WT\_VS\_PPARG\_KO\_VISCERAL\_ADIPOSE\_TISSUE\_TCONV\_DN |  | 31 | 0.11 | 0.55 | 0.970 | 1.000 | 1.000 | 816 | tags=71%, list=58%, signal=165% |
| 1236 | GSE27786\_BCELL\_VS\_NKTCELL\_UP |  | 15 | 0.14 | 0.55 | 0.970 | 1.000 | 1.000 | 1209 | tags=100%, list=86%, signal=691% |
| 1237 | GSE2770\_UNTREATED\_VS\_TGFB\_AND\_IL4\_TREATED\_ACT\_CD4\_TCELL\_2H\_DN |  | 19 | 0.13 | 0.55 | 0.972 | 1.000 | 1.000 | 241 | tags=21%, list=17%, signal=25% |
| 1238 | GSE17721\_LPS\_VS\_PAM3CSK4\_16H\_BMDC\_DN |  | 21 | 0.13 | 0.55 | 0.962 | 1.000 | 1.000 | 583 | tags=48%, list=41%, signal=80% |
| 1239 | GSE1791\_CTRL\_VS\_NEUROMEDINU\_IN\_T\_CELL\_LINE\_0.8H\_UP |  | 21 | 0.13 | 0.55 | 0.964 | 1.000 | 1.000 | 490 | tags=38%, list=35%, signal=57% |
| 1240 | GSE39864\_WT\_VS\_GATA3\_KO\_TREG\_DN |  | 26 | 0.12 | 0.54 | 0.950 | 1.000 | 1.000 | 75 | tags=8%, list=5%, signal=8% |
| 1241 | GSE17721\_CTRL\_VS\_CPG\_6H\_BMDC\_DN |  | 19 | 0.13 | 0.54 | 0.958 | 1.000 | 1.000 | 227 | tags=21%, list=16%, signal=25% |
| 1242 | GSE3691\_CONVENTIONAL\_VS\_PLASMACYTOID\_DC\_SPLEEN\_UP |  | 23 | 0.12 | 0.54 | 0.950 | 1.000 | 1.000 | 1243 | tags=100%, list=88%, signal=826% |
| 1243 | GSE36476\_CTRL\_VS\_TSST\_ACT\_72H\_MEMORY\_CD4\_TCELL\_YOUNG\_DN |  | 20 | 0.13 | 0.54 | 0.963 | 1.000 | 1.000 | 1 | tags=5%, list=0%, signal=5% |
| 1244 | GSE21927\_SPLEEN\_VS\_4T1\_TUMOR\_MONOCYTE\_BALBC\_DN |  | 21 | 0.12 | 0.53 | 0.969 | 1.000 | 1.000 | 583 | tags=48%, list=41%, signal=80% |
| 1245 | GSE9037\_CTRL\_VS\_LPS\_1H\_STIM\_IRAK4\_KO\_BMDM\_DN |  | 21 | 0.12 | 0.53 | 0.967 | 1.000 | 1.000 | 1 | tags=5%, list=0%, signal=5% |
| 1246 | GSE17721\_PAM3CSK4\_VS\_GADIQUIMOD\_0.5H\_BMDC\_UP |  | 23 | 0.12 | 0.53 | 0.969 | 1.000 | 1.000 | 57 | tags=9%, list=4%, signal=9% |
| 1247 | GSE13522\_CTRL\_VS\_T\_CRUZI\_BRAZIL\_STRAIN\_INF\_SKIN\_UP |  | 17 | 0.13 | 0.53 | 0.966 | 1.000 | 1.000 | 275 | tags=24%, list=19%, signal=29% |
| 1248 | GSE5589\_LPS\_VS\_LPS\_AND\_IL6\_STIM\_MACROPHAGE\_45MIN\_DN |  | 26 | 0.11 | 0.53 | 0.958 | 1.000 | 1.000 | 861 | tags=77%, list=61%, signal=194% |
| 1249 | GSE40274\_CTRL\_VS\_LEF1\_TRANSDUCED\_ACTIVATED\_CD4\_TCELL\_DN |  | 18 | 0.13 | 0.53 | 0.966 | 1.000 | 1.000 | 1230 | tags=100%, list=87%, signal=770% |
| 1250 | GSE7460\_FOXP3\_MUT\_VS\_HET\_ACT\_TCONV\_DN |  | 17 | 0.13 | 0.53 | 0.963 | 1.000 | 1.000 | 567 | tags=47%, list=40%, signal=78% |
| 1251 | GSE40493\_BCL6\_KO\_VS\_WT\_TREG\_UP |  | 21 | 0.12 | 0.52 | 0.978 | 1.000 | 1.000 | 1240 | tags=100%, list=88%, signal=813% |
| 1252 | GSE23925\_LIGHT\_ZONE\_VS\_NAIVE\_BCELL\_DN |  | 20 | 0.13 | 0.52 | 0.971 | 1.000 | 1.000 | 889 | tags=80%, list=63%, signal=213% |
| 1253 | GSE20198\_IL12\_VS\_IL12\_IL18\_TREATED\_ACT\_CD4\_TCELL\_DN |  | 22 | 0.12 | 0.52 | 0.968 | 1.000 | 1.000 | 1085 | tags=95%, list=77%, signal=407% |
| 1254 | GSE24574\_BCL6\_HIGH\_VS\_LOW\_TFH\_CD4\_TCELL\_DN |  | 23 | 0.12 | 0.52 | 0.959 | 1.000 | 1.000 | 1 | tags=4%, list=0%, signal=4% |
| 1255 | GSE8621\_UNSTIM\_VS\_LPS\_PRIMED\_UNSTIM\_MACROPHAGE\_DN |  | 29 | 0.11 | 0.51 | 0.979 | 1.000 | 1.000 | 743 | tags=62%, list=53%, signal=128% |
| 1256 | GSE43955\_TH0\_VS\_TGFB\_IL6\_TH17\_ACT\_CD4\_TCELL\_20H\_DN |  | 21 | 0.12 | 0.51 | 0.962 | 1.000 | 1.000 | 454 | tags=33%, list=32%, signal=48% |
| 1257 | GSE16385\_MONOCYTE\_VS\_12H\_IL4\_TREATED\_MACROPHAGE\_DN |  | 15 | 0.13 | 0.51 | 0.987 | 1.000 | 1.000 | 1094 | tags=93%, list=78%, signal=411% |
| 1258 | GSE29949\_MICROGLIA\_BRAIN\_VS\_CD8\_NEG\_DC\_SPLEEN\_UP |  | 21 | 0.12 | 0.51 | 0.968 | 1.000 | 1.000 | 525 | tags=43%, list=37%, signal=67% |
| 1259 | GSE36826\_NORMAL\_VS\_STAPH\_AUREUS\_INF\_IL1R\_KO\_SKIN\_UP |  | 15 | 0.13 | 0.51 | 0.982 | 1.000 | 1.000 | 15 | tags=7%, list=1%, signal=7% |
| 1260 | GSE21033\_CTRL\_VS\_POLYIC\_STIM\_DC\_24H\_DN |  | 20 | 0.12 | 0.51 | 0.982 | 1.000 | 1.000 | 985 | tags=85%, list=70%, signal=278% |
| 1261 | GSE23505\_UNTREATED\_VS\_4DAY\_IL6\_IL1\_TGFB\_TREATED\_CD4\_TCELL\_UP |  | 20 | 0.12 | 0.51 | 0.970 | 1.000 | 1.000 | 306 | tags=25%, list=22%, signal=31% |
| 1262 | GSE17301\_CTRL\_VS\_48H\_ACD3\_ACD28\_STIM\_CD8\_TCELL\_UP |  | 18 | 0.12 | 0.50 | 0.974 | 1.000 | 1.000 | 83 | tags=11%, list=6%, signal=12% |
| 1263 | GSE2405\_0H\_VS\_12H\_A\_PHAGOCYTOPHILUM\_STIM\_NEUTROPHIL\_DN |  | 15 | 0.13 | 0.50 | 0.973 | 1.000 | 1.000 | 11 | tags=7%, list=1%, signal=7% |
| 1264 | GSE1925\_CTRL\_VS\_24H\_IFNG\_STIM\_MACROPHAGE\_UP |  | 21 | 0.12 | 0.50 | 0.978 | 1.000 | 1.000 | 1246 | tags=100%, list=88%, signal=842% |
| 1265 | GSE6674\_PL2\_3\_VS\_ANTI\_IGM\_AND\_CPG\_STIM\_BCELL\_UP |  | 23 | 0.11 | 0.50 | 0.986 | 1.000 | 1.000 | 888 | tags=78%, list=63%, signal=208% |
| 1266 | GSE360\_L\_DONOVANI\_VS\_B\_MALAYI\_LOW\_DOSE\_MAC\_DN |  | 19 | 0.12 | 0.50 | 0.987 | 1.000 | 1.000 | 1044 | tags=89%, list=74%, signal=339% |
| 1267 | GSE4984\_GALECTIN1\_VS\_VEHICLE\_CTRL\_TREATED\_DC\_DN |  | 15 | 0.13 | 0.50 | 0.975 | 1.000 | 1.000 | 11 | tags=7%, list=1%, signal=7% |
| 1268 | GSE19401\_PAM2CSK4\_VS\_RETINOIC\_ACID\_AND\_PAM2CSK4\_STIM\_FOLLICULAR\_DC\_DN |  | 18 | 0.12 | 0.50 | 0.975 | 1.000 | 1.000 | 707 | tags=61%, list=50%, signal=121% |
| 1269 | GSE17721\_PAM3CSK4\_VS\_CPG\_0.5H\_BMDC\_UP |  | 23 | 0.11 | 0.50 | 0.980 | 1.000 | 1.000 | 1254 | tags=100%, list=89%, signal=884% |
| 1270 | GSE13522\_CTRL\_VS\_T\_CRUZI\_Y\_STRAIN\_INF\_SKIN\_IFNG\_KO\_UP |  | 21 | 0.11 | 0.49 | 0.983 | 1.000 | 1.000 | 1251 | tags=100%, list=89%, signal=869% |
| 1271 | GSE19888\_NO\_PRETREAT\_VS\_ADENOSINE\_A3R\_INHIBITOR\_PRETREATED\_MAST\_CELL\_TCELL\_MEMBRANES\_ACT\_DN |  | 18 | 0.12 | 0.49 | 0.983 | 1.000 | 1.000 | 819 | tags=72%, list=58%, signal=170% |
| 1272 | GSE360\_L\_DONOVANI\_VS\_L\_MAJOR\_MAC\_UP |  | 20 | 0.12 | 0.49 | 0.976 | 1.000 | 1.000 | 494 | tags=40%, list=35%, signal=61% |
| 1273 | GSE33292\_DN3\_THYMOCYTE\_VS\_TCELL\_LYMPHOMA\_FROM\_TCF1\_KO\_UP |  | 25 | 0.11 | 0.49 | 0.983 | 1.000 | 1.000 | 602 | tags=48%, list=43%, signal=82% |
| 1274 | GSE29949\_MICROGLIA\_BRAIN\_VS\_MONOCYTE\_BONE\_MARROW\_DN |  | 25 | 0.10 | 0.49 | 0.985 | 1.000 | 1.000 | 980 | tags=84%, list=69%, signal=270% |
| 1275 | GSE32255\_WT\_UNSTIM\_VS\_JMJD2D\_KNOCKDOWN\_4H\_LPS\_STIM\_DC\_UP |  | 22 | 0.11 | 0.49 | 0.983 | 1.000 | 1.000 | 387 | tags=32%, list=27%, signal=43% |
| 1276 | GSE17974\_0.5H\_VS\_72H\_IL4\_AND\_ANTI\_IL12\_ACT\_CD4\_TCELL\_DN |  | 17 | 0.12 | 0.49 | 0.979 | 1.000 | 1.000 | 1122 | tags=94%, list=80%, signal=454% |
| 1277 | GSE25088\_CTRL\_VS\_ROSIGLITAZONE\_STIM\_STAT6\_KO\_MACROPHAGE\_UP |  | 20 | 0.11 | 0.49 | 0.990 | 1.000 | 1.000 | 353 | tags=30%, list=25%, signal=39% |
| 1278 | GSE3982\_NKCELL\_VS\_TH2\_DN |  | 20 | 0.11 | 0.48 | 0.974 | 1.000 | 1.000 | 558 | tags=45%, list=40%, signal=73% |
| 1279 | GSE17721\_LPS\_VS\_POLYIC\_2H\_BMDC\_DN |  | 25 | 0.10 | 0.48 | 0.988 | 1.000 | 1.000 | 263 | tags=20%, list=19%, signal=24% |
| 1280 | GSE1460\_INTRATHYMIC\_T\_PROGENITOR\_VS\_NAIVE\_CD4\_TCELL\_ADULT\_BLOOD\_UP |  | 23 | 0.11 | 0.48 | 0.978 | 1.000 | 1.000 | 1 | tags=4%, list=0%, signal=4% |
| 1281 | GSE19941\_UNSTIM\_VS\_LPS\_STIM\_IL10\_KO\_NFKBP50\_KO\_MACROPHAGE\_DN |  | 15 | 0.13 | 0.48 | 0.988 | 1.000 | 1.000 | 11 | tags=7%, list=1%, signal=7% |
| 1282 | GSE18791\_UNSTIM\_VS\_NEWCATSLE\_VIRUS\_DC\_18H\_DN |  | 16 | 0.12 | 0.48 | 0.986 | 1.000 | 1.000 | 124 | tags=13%, list=9%, signal=14% |
| 1283 | GSE36476\_CTRL\_VS\_TSST\_ACT\_40H\_MEMORY\_CD4\_TCELL\_YOUNG\_DN |  | 26 | 0.10 | 0.48 | 0.993 | 1.000 | 1.000 | 1 | tags=4%, list=0%, signal=4% |
| 1284 | GSE10239\_KLRG1INT\_VS\_KLRG1HIGH\_EFF\_CD8\_TCELL\_DN |  | 20 | 0.11 | 0.48 | 0.988 | 0.999 | 1.000 | 484 | tags=40%, list=34%, signal=60% |
| 1285 | GSE37301\_HEMATOPOIETIC\_STEM\_CELL\_VS\_LYMPHOID\_PRIMED\_MPP\_UP |  | 20 | 0.11 | 0.48 | 0.976 | 0.998 | 1.000 | 87 | tags=10%, list=6%, signal=11% |
| 1286 | GSE17721\_CPG\_VS\_GARDIQUIMOD\_0.5H\_BMDC\_UP |  | 22 | 0.11 | 0.48 | 0.989 | 0.998 | 1.000 | 1080 | tags=91%, list=77%, signal=381% |
| 1287 | GSE41867\_LCMV\_ARMSTRONG\_VS\_CLONE13\_DAY6\_EFFECTOR\_CD8\_TCELL\_UP |  | 20 | 0.11 | 0.47 | 0.976 | 0.998 | 1.000 | 247 | tags=20%, list=18%, signal=24% |
| 1288 | GSE19888\_ADENOSINE\_A3R\_INH\_VS\_ACT\_IN\_MAST\_CELL\_UP |  | 15 | 0.12 | 0.47 | 0.986 | 0.997 | 1.000 | 422 | tags=33%, list=30%, signal=47% |
| 1289 | GSE42021\_TREG\_PLN\_VS\_CD24LO\_TREG\_THYMUS\_DN |  | 22 | 0.11 | 0.47 | 0.983 | 0.997 | 1.000 | 1158 | tags=95%, list=82%, signal=524% |
| 1290 | GSE15330\_WT\_VS\_IKAROS\_KO\_MEGAKARYOCYTE\_ERYTHROID\_PROGENITOR\_UP |  | 20 | 0.11 | 0.47 | 0.985 | 0.997 | 1.000 | 1255 | tags=100%, list=89%, signal=892% |
| 1291 | GSE17721\_CTRL\_VS\_LPS\_12H\_BMDC\_UP |  | 17 | 0.12 | 0.47 | 0.995 | 0.996 | 1.000 | 432 | tags=35%, list=31%, signal=50% |
| 1292 | GSE17721\_0.5H\_VS\_12H\_LPS\_BMDC\_DN |  | 17 | 0.11 | 0.46 | 0.984 | 0.998 | 1.000 | 216 | tags=18%, list=15%, signal=21% |
| 1293 | GSE4590\_LARGE\_PRE\_BCELL\_VS\_VPREB\_POS\_LARGE\_PRE\_BCELL\_UP |  | 18 | 0.11 | 0.44 | 0.993 | 0.999 | 1.000 | 964 | tags=83%, list=68%, signal=260% |
| 1294 | GSE1460\_INTRATHYMIC\_T\_PROGENITOR\_VS\_CD4\_THYMOCYTE\_UP |  | 21 | 0.10 | 0.43 | 0.988 | 1.000 | 1.000 | 98 | tags=10%, list=7%, signal=10% |
| 1295 | GSE24634\_TEFF\_VS\_TCONV\_DAY5\_IN\_CULTURE\_UP |  | 23 | 0.09 | 0.41 | 1.000 | 1.000 | 1.000 | 5 | tags=4%, list=0%, signal=4% |
| 1296 | GSE4984\_GALECTIN1\_VS\_LPS\_STIM\_DC\_DN |  | 23 | 0.09 | 0.41 | 0.995 | 1.000 | 1.000 | 83 | tags=9%, list=6%, signal=9% |
| 1297 | GSE40666\_NAIVE\_VS\_EFFECTOR\_CD8\_TCELL\_WITH\_IFNA\_STIM\_90MIN\_DN |  | 23 | 0.09 | 0.39 | 0.995 | 1.000 | 1.000 | 552 | tags=43%, list=39%, signal=70% |
| 1298 | GSE19401\_PAM2CSK4\_VS\_RETINOIC\_ACID\_STIM\_FOLLICULAR\_DC\_DN |  | 20 | 0.10 | 0.39 | 0.998 | 1.000 | 1.000 | 375 | tags=30%, list=27%, signal=40% |
| 1299 | GSE2770\_UNTREATED\_VS\_IL4\_TREATED\_ACT\_CD4\_TCELL\_48H\_UP |  | 21 | 0.09 | 0.38 | 0.998 | 1.000 | 1.000 | 16 | tags=5%, list=1%, signal=5% |
| 1300 | GSE40273\_EOS\_KO\_VS\_WT\_TREG\_DN |  | 20 | 0.09 | 0.38 | 0.998 | 1.000 | 1.000 | 20 | tags=5%, list=1%, signal=5% |
| 1301 | GSE7568\_IL4\_VS\_IL4\_AND\_TGFB\_TREATED\_MACROPHAGE\_24H\_DN |  | 21 | 0.08 | 0.35 | 1.000 | 1.000 | 1.000 | 455 | tags=33%, list=32%, signal=48% |
| 1302 | GSE17721\_CTRL\_VS\_GARDIQUIMOD\_0.5H\_BMDC\_DN |  | 16 | 0.09 | 0.34 | 1.000 | 1.000 | 1.000 | 652 | tags=50%, list=46%, signal=92% |
Table: Gene sets enriched in phenotype **WT (3 samples) **[plain text format]****
